# Unveiling microbial preservation under hyperacidic and oxidizing conditions in the Oligocene Rio Tinto deposit

**DOI:** 10.1038/s41598-021-00730-8

**Published:** 2021-11-02

**Authors:** David C. Fernández-Remolar, Daniel Carrizo, Mourad Harir, Ting Huang, Ricardo Amils, Philippe Schmitt-Kopplin, Laura Sánchez-García, David Gomez-Ortiz, Per Malmberg

**Affiliations:** 1 CEA, CNRS, IBS, Metalloproteins Unit, Université Grenoble Alpes, 38000 Grenoble, France; 2 Centro de Astrobiología (INTA-CSIC), Madrid, Spain; 3 Research Unit Analytical Biogeochemistry, Helmholtz Zentrum München, Neuherberg, Germany; 4 State Key Laboratory of Lunar and Planetary Sciences, Macau University of Science and Technology, Macau, China; 5 Centro de Biología Molecular Severo Ochoa, Universidad Autónoma de Madrid, Madrid, Spain; 6 Chair of Analytical Food Chemistry, Technical University Munich, 85354 Freising-Weihenstephan, Germany; 7 ESCET-Área de Geología, Universidad Rey Juan Carlos, 28933 Móstoles, Madrid, Spain; 8 Chemistry and Chemical Engineering, Chalmers University of Technology, Kemivägen 10, 412 96 Gothenburg, Sweden; 9 Present Address: State Key Laboratory of Lunar and Planetary Sciences, Macau University of Science and Technology, Macau, 999078 PR China; 10 Present Address: CNSA Macau Center for Space Exploration and Science, Macau, 999078 PR China

**Keywords:** Planetary science, Astrobiology

## Abstract

The preservation of biosignatures on Mars is largely associated with extensive deposits of clays formed under mild early Noachian conditions (> 3.9 Ga). They were followed by widespread precipitation of acidic sulfates considered adverse for biomolecule preservation. In this paper, an exhaustive mass spectrometry investigation of ferric subsurface materials in the Rio Tinto gossan deposit (~ 25 Ma) provides evidence of well-preserved molecular biosignatures under oxidative and acidic conditions. Time of flight secondary ion mass spectrometry (ToF–SIMS) analysis shows a direct association between physical-templating biological structures and molecular biosignatures. This relation implies that the quality of molecular preservation is exceptional and provides information on microbial life formerly operating in the shallow regions of the Rio Tinto subsurface. Consequently, low-pH oxidative environments on Mars could also record molecular information about ancient life in the same way as the Noachian clay-rich deposits.

## Introduction

The surface of Mars is widely acknowledged to be inhospitable for life because of the direct and indirect damaging effects of solar radiation^1^. Although there are a few bacterial forms that could potentially thrive under such extreme conditions^2^, their occurrence would be so low that the chances of detecting them would be negligible. Given these conditions, the subsurface areas of Mars have been claimed to host a potential biosphere as a consequence of its planetary evolution^3,4^. While the subsurface areas are considered a primary target for searching for extant or extinct life on Mars^5^, this is not the case for surface or subsurface materials affected or derived from the hydrochemical activity of the strongly oxidizing and acidic solutions, which mostly occurred during the Hesperian (< 3.7 Ga)^6^. Although different authors report neutral to alkaline conditions for underground Mars^7,8^, the upper part of the planet interior has certainly been affected by the circulation and downwelling of low-pH and oxidizing solutions^9–11^. Under such conditions, interactions between the oxidizing and acidic aggressive solutions and the Martian crust should have enhanced mineralization from ion dissolution and the release of byproducts at the surface, such as different Al-bearing clays whose composition depends on the weathering degree^12^. Among other salts, different Fe-bearing compounds, such as sulfates and oxides, could subsequently precipitate underground^13^ by oversaturation, which would lead to mineralization by ferruginous materials infilling the substrate faults and cracks or cementing the porosity. During mineralization, different microbial forms thriving in the subsurface could eventually be entombed inside a saline mineral matrix by simple oversaturation and precipitation of the incoming solutions. The mineralization process could also be promoted by the microbial activity itself through direct or indirect mechanisms that can trigger or even increase mineral precipitation on the cell surface^14,15^.

Iron oxides have been claimed to preserve molecular biosignatures with implications for detecting traces of life on Mars^16^. However, acidic and oxidizing environments have currently been ruled out as ideal conditions for the preservation of molecular biosignatures. This is also the case for Martian materials that have resulted from alteration or precipitation due to the activity of such acidic and oxidizing fluids^17,18^. The reason lies in the fact that these solutions inherit a high oxidizing capacity that can be released through different biotic or abiotic pathways. If life arose on Mars, it could have been a very active agent for recycling inorganic and organic compounds by using inorganic electron acceptors (e.g., Fe^3+^ and SO_4_^2−^) that are abundant in acidic fluids^19^. Although the solar X-ray and UV irradiation rates occurring in the Hesperian are unknown, different oxidants that would surely have been produced by incident radiation on the atmosphere and hydrosphere^7,20^ would have oxidized biomolecules and organic compounds without the participation of microbes. As the preservation potential of molecular biosignatures under acidic conditions is considered very low, the planetary community has ruled out the list of potential targets to search for molecular evidence of extinct life on Mars^17^. Therefore, deposits formed under mild conditions (neutral pH, low oxidizing to reducing conditions) have become first-class targets to find traces of living organisms on early Mars^21^.

On Earth, records of ancient life activity can occur in the form of physical structures replicated by mineral precipitation^22^. Indeed, as the final diagenetic byproducts of acidic deposits are goethite (FeOOH) and hematite (Fe_2_O_3_), the preservation potential of mineralized structures is extremely high. The diagenetic processes affecting acidic materials consist of a combination of loss of sulfate ions and dehydration under oxic conditions that are controlled by the inflow of meteoric solutions after exhumation of materials from the initial acidic conditions^23^. Very recently, a diverse association of lipids has been detected in modern to recent materials formed under acidic and oxidizing conditions^24^, which suggests that organics can be preserved in the first stage of diagenesis. Indeed, the preservation potential of acidic deposits for organic compounds has been wrongly dismissed, as different organic compounds^25^, including evidence of peptide chains^26^, have been found in the 2-Ma upper terrace of Rio Tinto.

In this paper, long-term preservation of complex molecular biosignatures in ancient Rio Tinto subsurface ferruginous deposits (~ 25 Ma) is targeted. This task was accomplished by analyzing samples through state-of-the-art time of flight secondary ion spectrometry (ToF–SIMS) with Fourier transform ion cyclotron resonance mass spectrometry (FTICR-MS) and gas chromatograph mass spectrometry (G-MS). The identification of large molecular species suggesting that the acidic ferruginous materials have a high preservation potential agrees with the finding of peptide chains^26^ in the oldest Rio Tinto terrace, which also requires exceptional preservation conditions for its persistence. In this regard, preservation is favored in the Rio Tinto acidic deposits despite high water availability for mineral transformation. As this was not the case for sediments formed under low-pH conditions on Mars with limited water^23^, high preservation should be expected in those Hesperian materials. In this context, the extensive ferruginous deposits formed in acidic and oxidizing Hesperian environments should be considered first-class targets to look for ancient traces of life if any biosphere emerged at some time on the red planet.

### Geobiological settings

**Figure 1:**
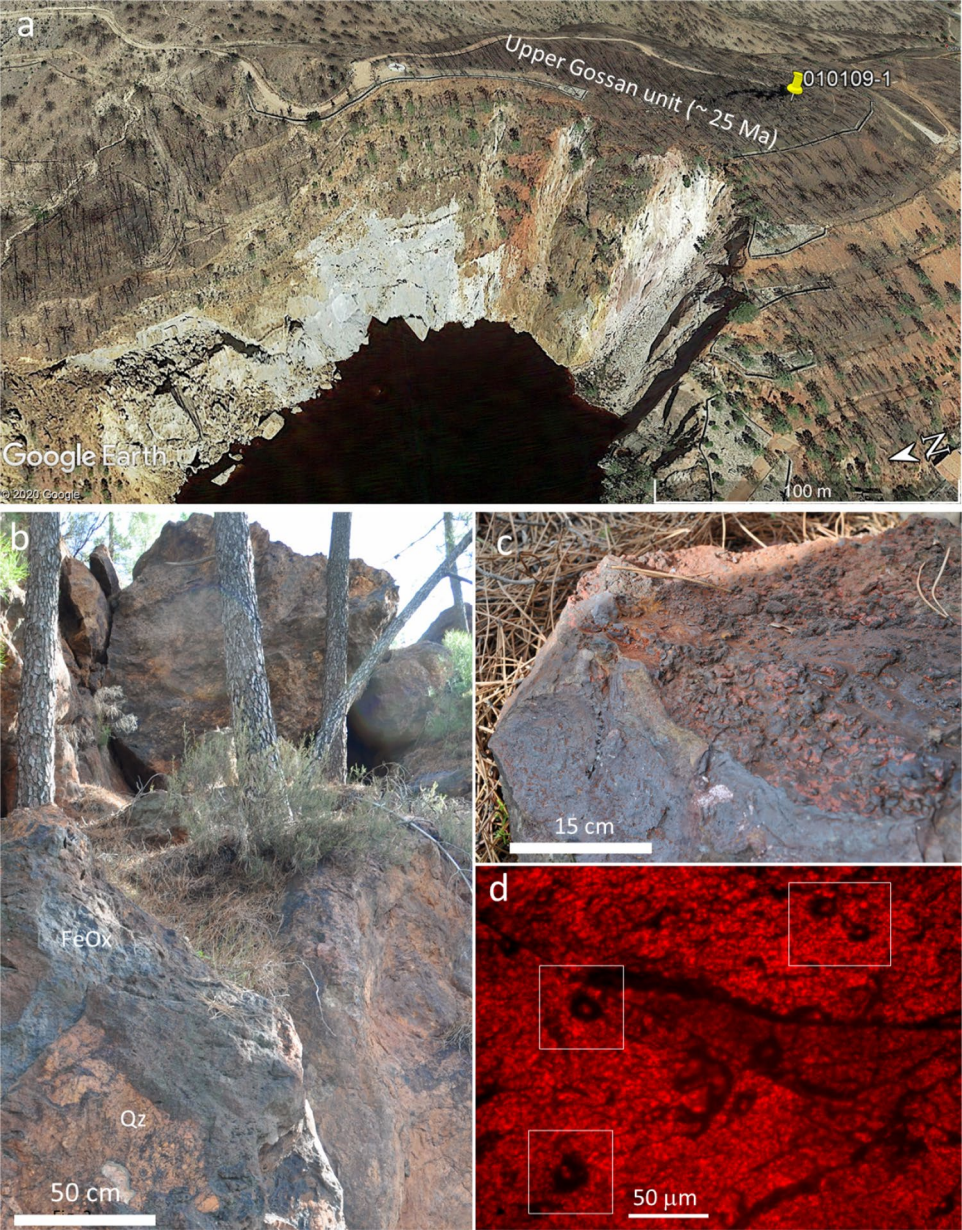
Occurrence of the Upper Gossan deposits in the Peña de Hierro area. (**a**) Google Earth satellite image of the Upper Gossan unit topping the Peña de Hierro where the sample 010,109–1 was collected. (**b**) Outcrop photograph of the lower part of the Upper Gossan unit consisting of iron oxide deposits (FeOx) infilling cracks and voids left by the alteration of sulfide materials, which are limited by quartz-rich materials (Qz). (**c**) Sample 010,109- was collected in the gossan material cropping out at a higher location in the Upper Gossan; it occurs as a lenticular body with internal massive fabric and topped with cm-sized popcorn and millimeter-thick filamentous structures. (**d**) Thin section of sample 010,109–1 around TA3 (see Fig. 7) showing a laminated fabric not recognized through observation by the naked eye; this sample contains different 30-micron ovoid elements of likely fungal origin; because they correspond with the preservation of filamentous structures, the ovoid sections suggest that they have the same orientation.

Gossan deposits occur in the Peña de Hierro area (Fig. 1), which is considered one of the sources of the acidic waters of Rio Tinto^12^. Indirect rock dating using geomagnetic techniques^27^ suggests that the gossan materials in this location were formed through the acidic weathering of Carboniferous sulfide orebodies^28^ during the late Oligocene^27^. Such alteration coincides with a thermal climatic event^29^ that likely enhanced the acidic weathering of the orebodies. This was followed by a drop in the water table in response to subsequent climatic evolution during the Messinian saline crisis starting by 6 Ma^27^.

The gossan originated from a complex direct and indirect chemolithotroph attack on pyrite (FeS_2_) orebodies^30^ through the following reactions:

FeS_2_ (s) + H_2_O (l) + 3.5·O_2_ (aq) → Fe^2+^ (aq) + 2·SO_4_^2−^ (aq) + 2·H^+^ (aq) (direct biooxidation of sulfur).

Fe^2+^ (aq) + 1/4·O_2_ (aq) + H^+^ (aq) → Fe^3+^ (aq) + 1/2·H_2_O (l) (biooxidation of released Fe^2+^).

where O_2_ is used as an electron acceptor for the oxidation of FeS_2_ and free Fe^2+^.

The ferric cation resulting from the microbial oxidation of Fe^2+^ becomes a secondary agent for pyrite oxidation through a subsequent abiotic process, where the sulfide anion is oxidized to sulfate^30^ to form Fe^2+^ again:

FeS_2_ (s) + 14·Fe^3+^ (aq) + 8·H_2_O (l) → 15·Fe^2+^ (aq) + 2·SO_4_^2−^ (aq) + 16·H^+^ (aq).

The solutions resulting from sulfide oxidation are eventually exposed to oversaturation, producing mainly acidic sulfates such as jarosite (e.g., H_3_O^+^Fe_3_(OH)_6_(SO_4_)_2_) and schwertmannite (Fe_8_O_8_(OH)_6_(SO_4_))^23^:

3·Fe^3+^ (aq) + M^+^ (aq) + 2·SO_4_^2+^ (aq) + 6·H_2_O (l) ↔ MFe_3_(OH)_6_(SO_4_)_2_ (s) + 6·H^+^ (aq).

where M = Na, K or H_3_O, and.

8·Fe^3+^ (aq) + SO_4_^2−^ (aq) + 14·H_2_O (l) ↔ Fe_8_O_8_(OH)_6_(SO_4_) (s) + 22·H^+^ (aq).

**Figure 2:**
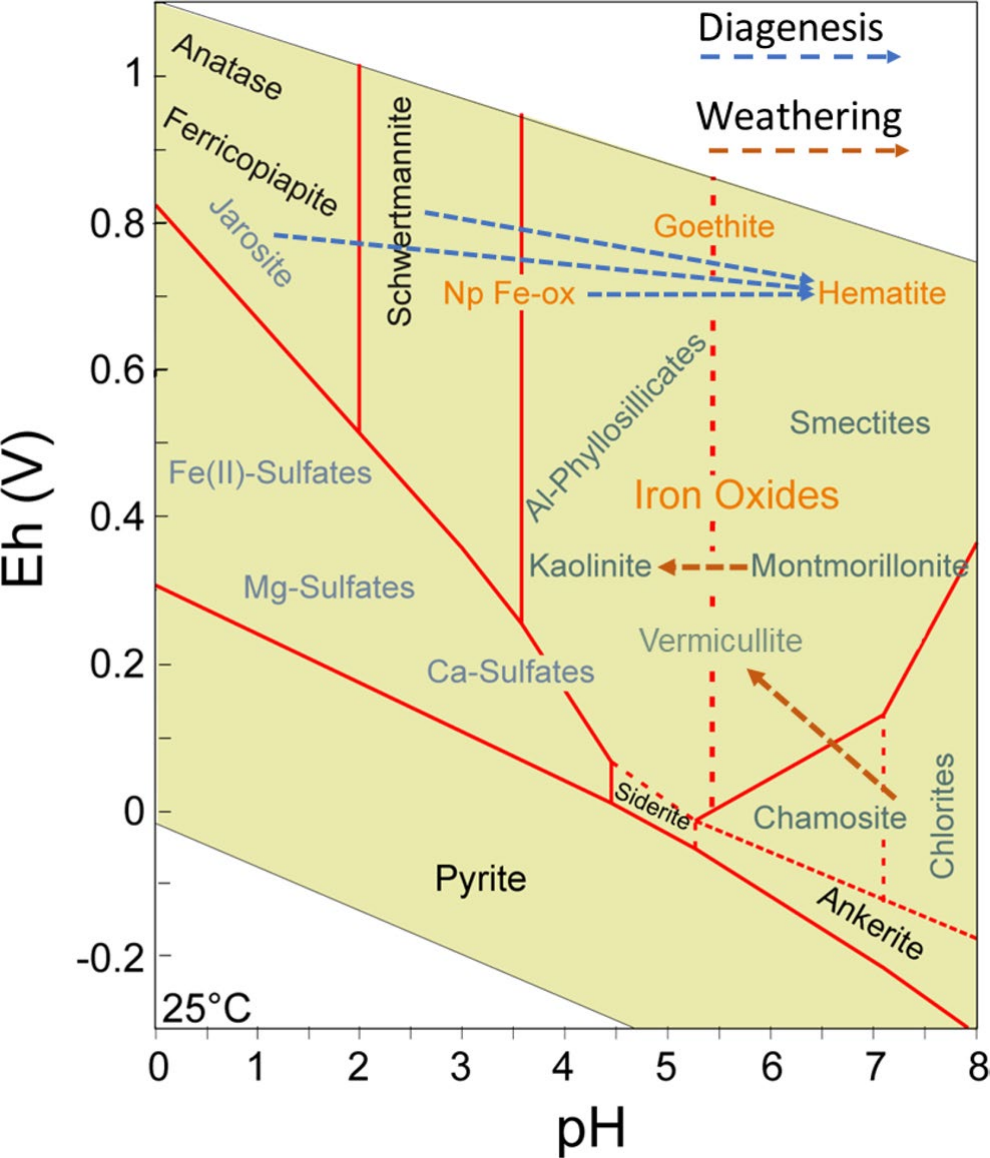
pH-Eh mineral stability diagram of different iron-bearing mineral phases and various clays. The blue arrows trace several diagenetic pathways starting in different Fe-bearing protoliths (e.g., sulfates and nanophase oxides), passing by an intermediate phase such as goethite (FeOOH), and ending in hematite (Fe_2_O_3_). The diagram shows that mineral maturation toward iron oxides requires hydrolysis and dehydration, which are controlled by changes in pH and Eh. The red arrows trace weathering pathways in the clays forming sedimentary deposits at the surface and subsurface of Rio Tinto.

The iron oxides that are the main mineral components of gossan and other underground ferric deposits in Peña del Hierro^13^ come from the diagenesis of primary acidic sulfates^31^ (Fig. 2) when they are exposed to meteoric solutions with a circumneutral pH (~ 6–7). The process involves a combination of hydrolysis and dehydration that results in removing the sulfate groups to produce oxyhydroxides such as goethite (FeOOH) after dissolution-reprecipitation^32^. In addition, hematite (Fe_2_O_3_) can be directly formed from acidic sulfates by dehydration, which is accelerated by temperature^31^. Furthermore, hematite can also be formed by dehydration of nanocrystalline goethite^32^ or nanophase iron oxides^23^ whose composition is unclear. The diagenesis of materials formed under acidic conditions (Fig. 2) is much more complex than a simple transformation of ferric sulfate to oxides. This diagenesis can involve different oxidation steps affecting primary ferrous sulfates (e.g., melanterite, FeSO_4_·7H_2_O) that precipitate in deeper areas under lower oxidizing conditions or through the recrystallization and hydrolysis of different sulfate phases by slight changes in the pH of the acidic solutions^10,32^. In the Peña del Hierro gossan, the abundance of hematite over other iron oxide species such as goethite^13,23^ suggests that the protolith was mainly composed of ferric sulfates that evolved to Fe_2_O_3_ during early diagenesis.

Such a complex diagenesis that involves an interplay among dramatic pH and redox changes, hydrolysis and dehydration should have prevented the preservation of molecular compounds with a biological origin. However, the detection of abundant and diverse biomolecules in the Rio Tinto gossan shows that different preservation mechanisms have played essential roles in the fossilization of organic compounds that were produced by microbial activity in the subsurface of Peña de Hierro.

## Results

To determine the preservation potential of ferruginous deposits that formed under acidic and oxidizing conditions, sample 010,109–1 (Fig. 1b–d) collected from the top of the Upper Gossan unit in Peña de Hierro (~ 25 Ma)^13,22,33^ was analyzed using different spectral techniques. Such analysis consisted of molecular mapping and characterization by means of three spectral techniques: FTICR-MS, GC–MS and ToF–SIMS.

### FTICR MS molecular fingerprinting

**Figure 3:**
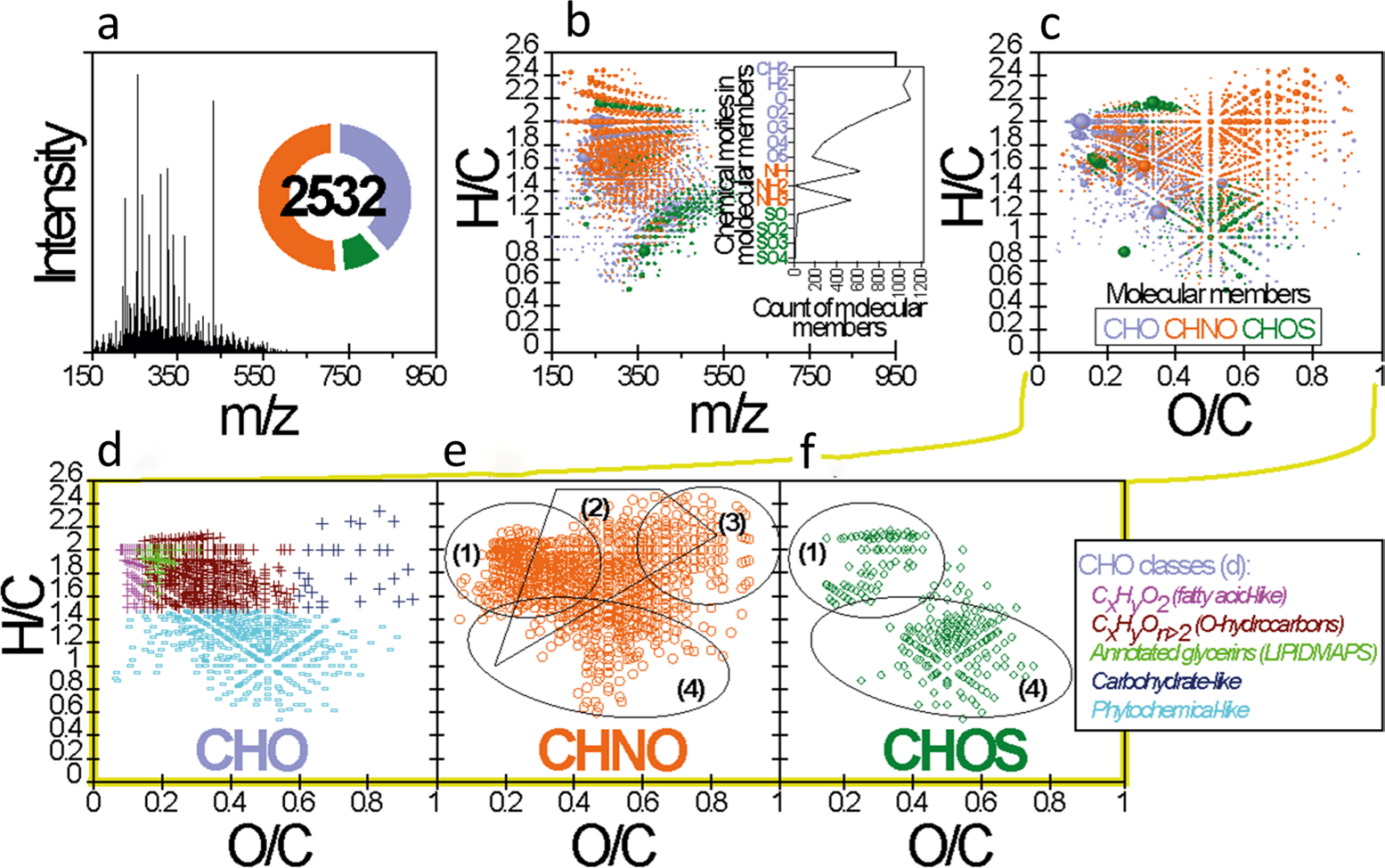
Main results obtained from the ESI(-)-FTICR-MS analysis. (**a**) Mass spectrum (150–950 Da) of the Upper Gossan sample 010,109–1. Inset number in (**a**) represents the total number of assigned molecular members (i.e., CHO, CHNO and CHOS). (**b**) and (**c**) H/C ratio versus mass-to-charge (*m/z*) plot and van Krevelen diagram of all assigned molecular members, respectively (color-coded dots represent molecular members CHO, blue; CHNO, orange; and CHOS, green, while circular areas indicate relative mass peak intensity). Inset in (**b**) represents the frequency histogram of the exact mass differences corresponding to CH_2_, H_2_, O_n(1≤n≤5)_, NH_n(1≤n≤3)_, and SO_n(1≤n≤4)_ (error range 0.1 ppm). (**d**), (**e**) and (**f**) represent compound classes included in CHO, CHNO and CHOS-molecular members, respectively. Highlighted areas in (**d**), (**e**) and (**f**) visualize the following compound classes: (1) Saturated/unsaturated compounds, (2) Amino acids-/peptide-like compounds, (3) Carbohydrate-like compounds, and (4) highly unsaturated and aromatic compounds.

Ultrahigh-resolution Fourier transform ion cyclotron mass spectrometry was used to examine the extracted Upper Gossan fraction characteristic of the original preserved organic biogeochemical signature. Enabling highly accurate mass measurement and mass resolution, FTICR MS of terrestrial dissolved organic matter (TDOM) provides several thousand mass peaks for individual samples, of which several were assigned to C, H, N, O, and S compositional space^34–36^. The FTICR mass spectrum of the extracted Upper Gossan fraction is shown in Fig. 3a. All mass signals detected correspond to singly charged ions and were assigned and classified as O, S, N-functionalized CHO, CHNO and CHOS molecular compositions (Fig. 3b–f). In fact, these molecular series are visualized in a van Krevelen plot (Fig. 3c), which is common to illustrate the molecular space of most complex natural samples^34,35,37^. The advantage of this plot is that it displays the assigned molecular species present in a sample rather than the computed average values. As shown in Fig. 3c, the van Krevelen plot of the Upper Gossan extract exhibits regular smooth and extensive distributions over large ranges of unsaturation (high H/C ratio), down to high degrees of oxygenation (high O/C ratio). Condensed compounds were relevant (Fig. 3c), had double bond equivalents (DBEs) higher than 3 with at least one or more phenyl rings, and were more abundant than saturated and unsaturated compounds with no phenyl ring (DBE ≤ 3) (Fig. S1).

Moreover, the extracted gossan fraction revealed significant chemodiversity mainly attributed to CHO (39.0%), CHNO (51.0%), and CHOS (10.0%) molecular compositions (Fig. 3d–f). While CHO-bearing molecules have shown the presence of saturated/unsaturated compounds (e.g., fatty acid-like, glycerins and oxygenated hydrocarbons CHO_n>2_), carbohydrate-like and carboxylic-rich alicyclic molecules (CRAM), including phytochemicals such as terpenes, flavonoids, lignins, polyphenol compounds and derivatives, as significant tracers of terrestrial organic matter (Fig. 3d), CHNO-bearing molecules have shown enrichment in oxygenated compounds, peptide-like compounds and aliphatics (Fig. 3e). Fewer CHNO-bearing molecules are localized in the CRAM region (Fig. 3e). Such enrichment in CHNO-bearing molecules suggests more preserved bioavailable organic matter and its derivatives, which are known as drivers of microbial community structure and fundamental metabolic strategies^38–40^. Conversely, CHOS-bearing molecules display a narrow range of hydrogen saturation H/C (0.55–2.2) evocative of reduced CHOS organic compounds and more oxygenated molecular species bounded by approximate O/C (0.25–0.75) and H/C (0.6–1.4) values characteristic of CRAM (Fig. 3f). The appearance of CRAM and derivative compounds in the lignin-like and polyphenol-like regions on the van Krevelen plot could possibly arise from vegetation (i.e., vascular/higher plants and/or algal polyphenols). Indeed, the formation of organics by transitional organic matter involving monosaccharides, amino acids or fatty acids produces long-term humic organics via abiotic and/or humification reactions^41^. In parallel, the generation of insoluble, nonhydrolyzable and nonbiodegradable organics by various organisms may also be involved in the production and preservation processes of organic matter^41,42^. Overall, this large molecular diversity found in the Upper Gossan extract within and across the assigned molecular compositions is an example of the diverse biogeochemical availability of organic compound signatures, which is an acknowledged aspect of progressive organic matter preservation in the environment.

### GC–MS molecular screening

The analysis of the total lipid extract (i.e., including nonpolar, polar and acidic fractions) revealed the presence of diverse lipid families (i.e., *normal* and branched alkanes, including isoprenoids; alkenes; branched alkanols; steroids and hopanoids; and *normal*, branched, and monounsaturated fatty acids) (Table S1). The nonpolar fraction was mostly composed of straight chain or *normal* alkanes (*n*-alkanes) with 11 to 31 carbons that showed a bimodal distribution with maxima at C_18_-C_21_, C_27_ and C_29_. In conjunction with the *n*-alkanes, the isoprenoids pristane and phytane were also found (Table S1). The acidic fraction was composed of *n-*fatty acids with 11 to 30 carbons, monounsaturated fatty acids with 16, 18 and 20 carbons, branched fatty acids of *iso*/*anteiso* configurations (i.e., methyl groups at ultimate or penultimate positions) with 13 to 17 carbons (from *i-/a*-C_13:0_ to *i/a*-C_17:0_), and other minor compounds (Table S1). The *n*-fatty acid series showed a dominance of even-over-odd carbons, with maximum peaks at C_24_, C_26_ and C_28_. Meanwhile, the polar fraction was primarily composed of *n*-alkanols with 11 to 24 carbons and even-over-odd preference, glycerol compounds suggesting the occurrence of glycolipids, a lupane derivative (lupan-3-ol, acetate), and a series of sterols, including cholesterol β-sitosterol, stigmasterol, and an ergostanol derivate (Table S1).

The observed lipid distribution patterns provide information about possible sources of organic matter in this sample, which are mainly related to bacteria, algae/phytoplankton and plants. The latter source is inferred from the detection in the sample of odd and high molecular weight (HMW) *n*-alkanes such as C_25_ for macrophytes and C_27_, C_29_, and C_31_ for terrestrial plants^43,44^. In contrast, the detection of low molecular weight (LMW) *n*-alkanes (< C_22_) is associated with either bacterial (mostly even LMW *n*-alkanes) or phytoplanktonic (odd LMW *n*-alkanes) sources^45^. Pristane and phytane have as a primary source the degradation of chlorophyll pigments. Both isoprenoids mainly originate from phytol, the esterifying alcohol of phototrophic chlorophylls^46^, whose degradation gives rise to pristane or phytane in the presence or absence of oxygen, respectively^47^.

Vegetal and microbial biomarkers are also found in the acidic and polar fractions, where LMW *n*-fatty acids and *n*-alkanols (i.e., < C_20_), as well as the hopanoid lupan-3-ol and acetate, are most likely associated with bacterial sources^48^, whereas HMW *n*-fatty acids and *n*-alkanols, as well as phytosterols (i.e., β-sitosterol or stigmasterol) can be related instead to macrophytes or land plants^43,49^. Sterols are ubiquitous constituents in all eukaryotic organisms, where stigmastanol or β-sitosterol is produced almost exclusively by higher plants^50^, whereas cholesterol is primarily produced by animals^51^. The presence of ergosterol can be related to fungal species^52^.

Certain monounsaturated fatty acids detected in the sample have been associated with biomass from cyanobacteria or microalgae (i.e., 16:1 ω7, 18:1 ω9)^53^. Furthermore, *iso-*/*anteiso-* pairs of fatty acids (mostly 15:0 and 17:0) are typically associated with bacterial sources^54^ and are particularly abundant in sulfate-reducing bacteria^55^.

### ToF–SIMS molecular mapping

**Figure 4:**
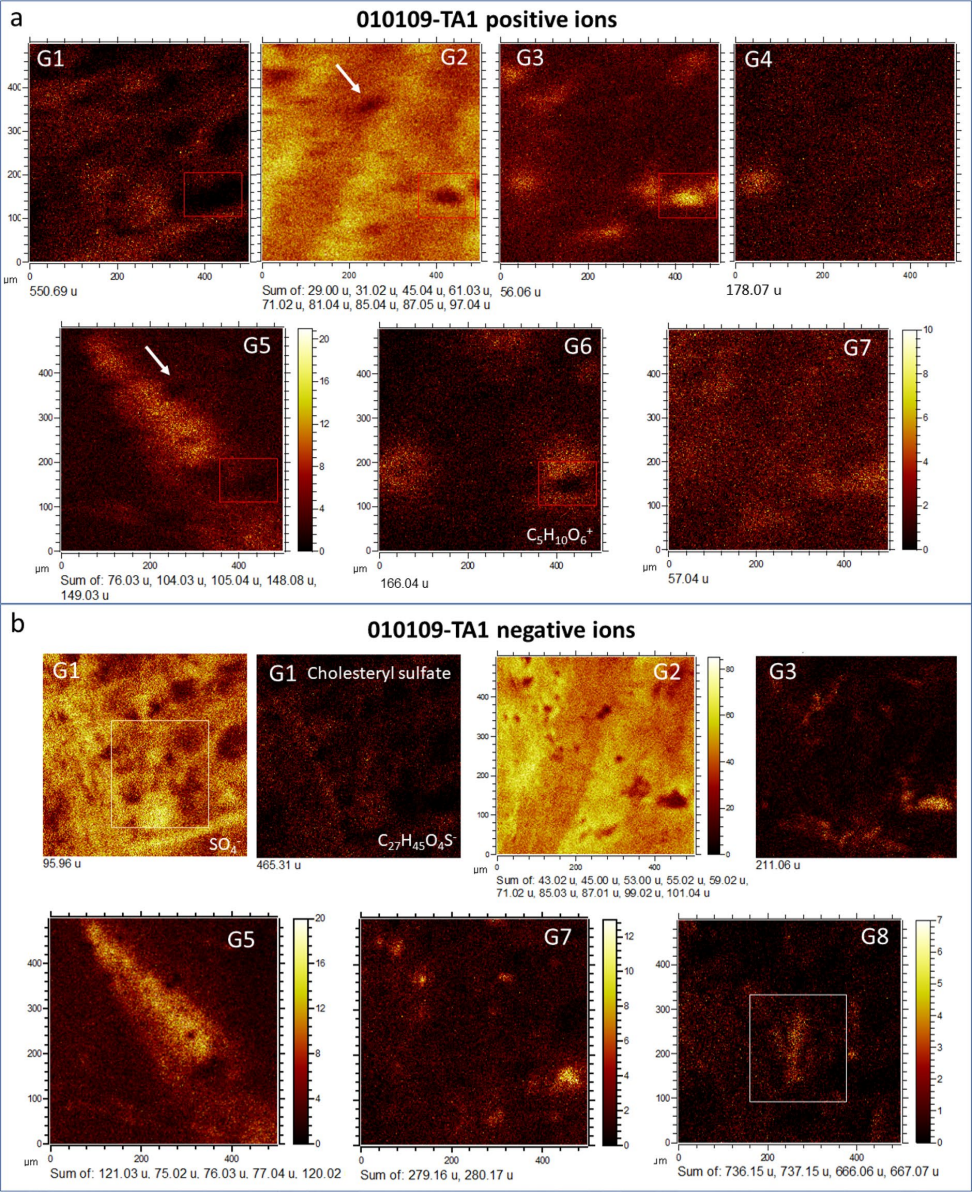
Occurrence of different biological microstructures in Target Area 1 (TA1) characterized by the imaging capabilities of ToF–SIMS as morphological Groups 1 to 8 through their molecular compositions. (**a**) Groups 1 to 8 outlined by the distribution of (**a**) positive and (**b**) negative fragments. The red rectangle traces a nodular microstructure in G3 occurring as an empty and complementary cast in G2, G5 and G6. The white rectangle encloses branched filamentous structures in G8 that complement part of the filamentous structures in G1 (see Fig. 5) suggesting that the fungal filamentous structure show compositional variations. The white arrow points at a void (see Supplementary Fig. 2) that is affecting some microstructures in G2 and G5. As discussed in the main text, the composition and structure of groups G1 to G8 suggest that the groups could come from the preservation of different biological structures such as filamentous fungi (G1 and G3), or plant fragments (G5 and G6).

**Figure 5:**
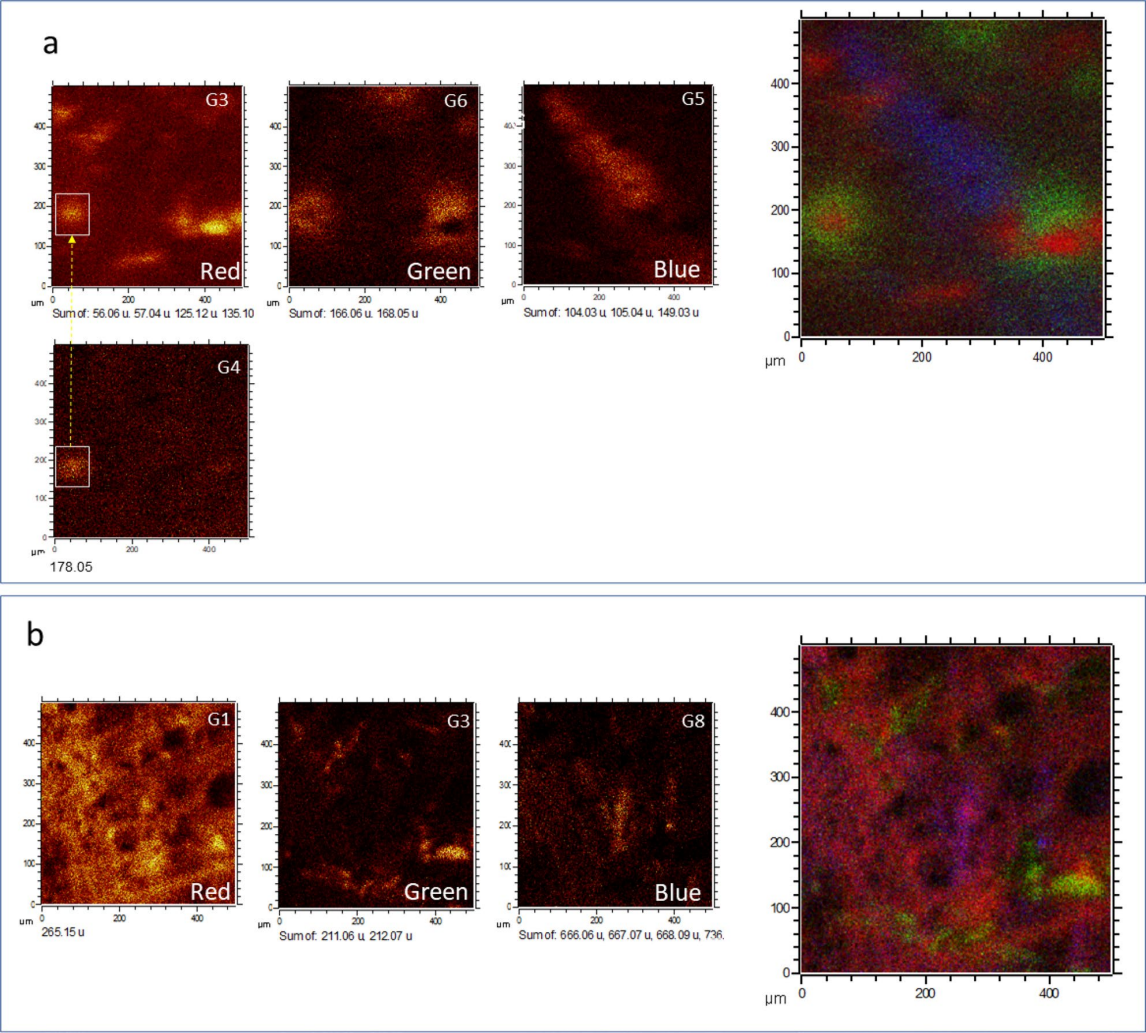
Couple of ToF–SIMS red/green/blue (RGB) overlays for (**a**) G3, G6 and G5 and (**b**) G1, G3, and G8 resulting in two different microstructure associations. The first RGB association (**a**) could correspond to compositional variations in plants (G5, G6, and G3) or to plant fragments (G5, and G6) affected by microbial activity (G3). Interestingly, G4 appears as a compositional variation in the G3 structure. The second RGB association (**b**) could include three different groups, namely, G1, G3 and G8 that could correspond to a microbial structure such as a biofilm in which the biomass is dominated by microbial forms biomineralizing sulfate. G3 structures interlock empty complementary areas in G1, suggesting that these areas could be the remains of different biological entities, while G8 branched microstructures merge with some filamentous units in G1, suggesting compositional variations in some specific zones of the filamentous mass of G1.

ToF–SIMS molecular imaging has allowed the characterization of different groups of molecules that are preferentially associated with distinctive morphologies, the sizes of which are generally smaller than 500 μm (see Table 1 where groups and morphologies are associated with compounds). Such morphologic groups show different fabrics and textural features when the target areas (TAs) 1 to 3 (TA1, TA2, and TA3) are analyzed (Figs. 3, 4, and 5; Figs. S2–S5). The distribution of ions with biomolecular origin follows the fabric and textural pattern outlined by the main inorganic ions of acidic oxysalts and oxyhydroxides (Figs. S2 to S3), including Fe^+^ (*m/z* 55.93), Al^+^ (*m/z* 26.98), K^+^ (*m/z* 38.96), Na^+^ (*m/z* 22.99), SO_2_^-^/SO_3_^-^ (*m/z* 63.96/79.96), and SiO_2_^-^ (*m/z* 63.96), while PO_2_^-^/PO_3_^-^ (*m/z* 62.96/78.96), and NO_2_^-^/NO_3_^-^ (*m/z* 45.99/61.99) anions occur as microstructures smaller than 50 microns (Fig. S4).

**Table 1:** Identification of the morphological groups through the ToF–SIMS molecular distribution found in the Upper Gossan sample of Peña de Hierro.

Group	Microstructure	Potential compounds		Target area (TA)
		m/z positive ions	m/z negative ions	
Group 1	Loosely to densely packed networks of < 50 micron thick filaments with strong mineralization shown by positive and negative sulfur-bearing ions	Lineal hydrocarbons fragmented and ionized from long lipids which form adducts with NH_4_^+^ likely sphingolipids	Fatty acids coming from lipidic structures like ceramides	TA1, TA2, TA3
		Salt composition of mineralized structures	SOx-bearing adducts of cholesterol-like sterols and lipids coming from the mineralized surface of fungal structures	
Group 2	Linear elements with an average thickness of > 80 microns disassociated from the other microstructures	Likely terpenoid fragments sourced in plants		TA1
Group 3	Ovoidal microstructures averaging 60 microns (TA1 and TA2); following larger microstructures and the mineral matrix in TA3	Fragments of amino acids and peptides. The occurrence of some ions in the TA3 matrix while others follow the biological microstructures suggest different a origin for them		TA1, TA2, TA3
Group 4	Two coupled elongated nodular microstructures in which the elongation axes connect both nodule centers (TA1); occurring as an empty cast in TA3	Unknown origin	n/a	TA1, TA3
Group 5	600 micron-long elongated triangle-like morphology crosscutting the main structure fabric of Groups 1 and 2; occurring as an empty cast in TA3	Plant terpenoids like piperonal derivatives		TA1, TA3
Group 6	Three 120-micron subcircular morphologies show an empty central void; occurring as an empty cast in TA3	Vanillic acid derivatives	n/a	TA1, TA3
Group 7	10- to 50-micron micronodules following the main pattern appearing in Groups 3 and 4	Plant terpenoids		TA1
Group 8	Structures with short branches with an average length of 200 microns that are transverse to the fabric of Group 1	n/a	Tannins, or some polysaccharides	TA1
Group 9	Densely packed network of 40-micron-long filament-like microstructures, which appear in TA2 along the sharp boundary that contacts Group 1; fits the primary microstructure found in TA3, while in the TA1 show a more homogeneous occurrence	Glycerolipids and glycerophospholipids with possible occurrence of glycerophosphocholines. Fatty acids fragmented from the lipidic structure. Abundant in TA2 and TA3, and secondary in TA1		TA2, TA3

**Figure 6:**
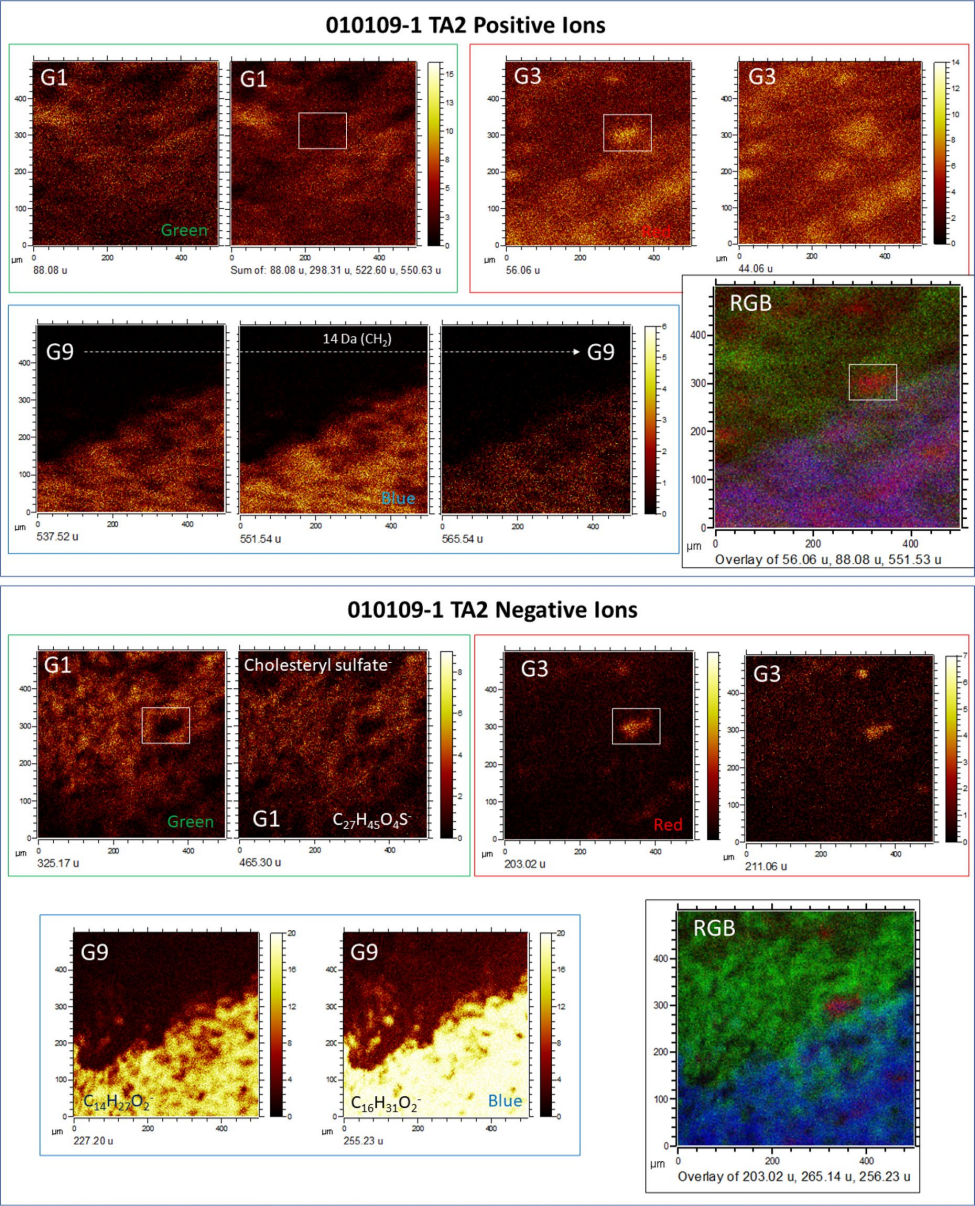
Characterization of possible microbial structures preserved in Target Area 2 (TA2) by using the ToF–SIMS molecular mapping capabilities. The image analysis follows the same morphological groups G1 to G8 adding one new group G9. Two different main structures defined by G1 and G9 are limited by a sharp boundary between them. The G1 microstructure is characterized by a strong intensity of different N- and S-bearing compounds that outline a diffuse filament network, whose primary composition would be large lipids such as ceramides. However, G9 shows a more massive filamentous mass that is traced by a high intensity in acylglycerides and FA fragments. G3 occurs as an independent group of microstructures that interlock both G1 and G9 microstructures. The RGB overlay shows two main microbial structures (G1 and G9) in which empty cavities would be occupied by a third microbial unit defined by G3. Interestingly, the G1 microbial structure is strongly mineralized by sulfate while G3 and G9 microstructures are mineralized by ferruginous materials, suggesting that they were formed in two separate mineralization episodes. The microbial structure and composition of G1 and G9 suggest that these groups could correspond to mineralized biofilms.

**Figure 7:**
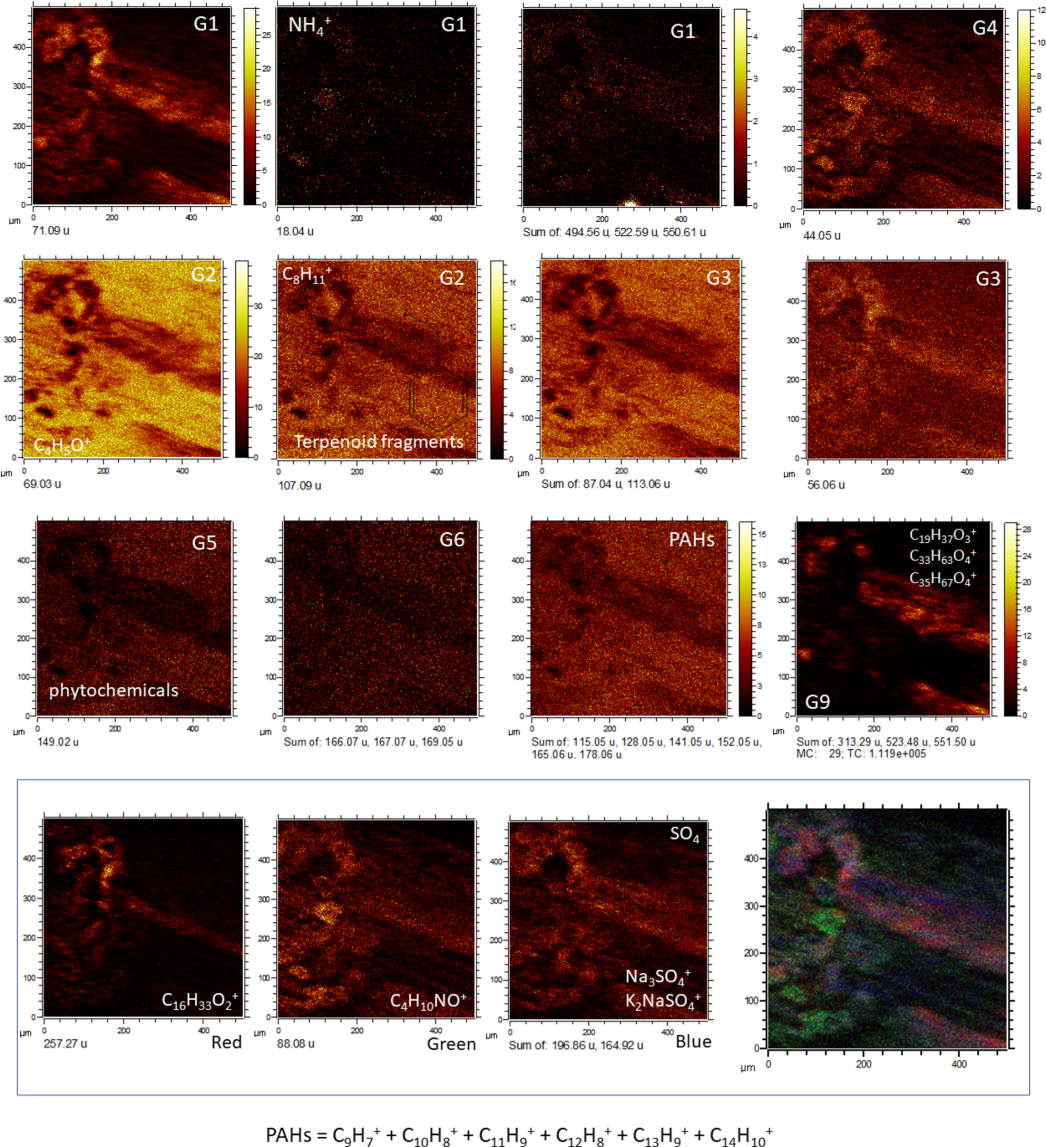
Identification of biological microstructures using ToF–SIMS molecular imaging in Target Area 3 (TA3). Groups G1, G3, G4, and G9 outline different oval elements (< 80 microns) that are associated with parallel straight structures longer than 500 microns. Interestingly, groups G2, G5, and G6, which have been associated with phytochemicals as well as PAHs, occur in the mineral matrix that encloses the biological structure. Such a distribution suggests that the microbial structure had been transported or was thriving in a water mass that was enriched in refractory organic compounds such as PAHs and some phytochemicals. The RGB overlay shows that the biological structure has compositional changes that represent sulfate biomineralization (Na_3_SO_4_^+^, and K_2_NaSO_4_^+^), as well as sphingolipid (C_4_H_10_NO^+^) and acylglyceride fragments (C_16_H_33_O_2_^+^). The PAH intensity image was obtained by adding the intensities of different PAHs such as C_9_H_7_^+^, C_10_H_8_^+^, C_11_H_9_^+^, C_12_H_8_^+^, C_13_H_9_^+^, and C_14_H_10_^+^ (see Supplementary Table 3).

In sample 010,109–1, TA fabric and texture fit the distribution of inorganic and organic ions well (Figs. 4, 5, 6, and 7; Figs. S2–S4), which mainly correspond to filamentous networks and isolated elements associated with organic compounds of biological origin (e.g., see Figs. 4, 5, 6, and 7). In some cases, ToF–SIMS ion mapping is the only technique for recognizing the microstructures of samples when this is not possible using different microscopic techniques (e.g., light or scanning electron microscopes). Furthermore, the TOF–SIMS ion-induced secondary electron images also provided additional information on the microstructure of the sample (Figs. S2–S4). By combining all the data sources retrieved via ToF–SIMS, the following morphological groups could be identified (Figs. 4, 5, 6, and 7).

*Group 1* This group is characterized by networks of < 50-micron-thick filaments (Figs. 4, 5, 6, and 7) showing strong mineralization by the enrichment in positive and negative sulfur-bearing ions such as Na_3_SO_4_^+^, K_2_NaSO_3_^+^, HS^-^, SO_2_^-^, and SO_3_^-^ at 164.92, 196.86, 32.98, 63.96, and 79.96 (Figs. S2–S5), respectively, as well as different cations such as Fe^+^ (55.94), K^+^ (38.97), Na^+^ (22.99) and Al^+^ (26.98) (Figs. S2 to S5). TA1 filamentous structures are thicker and looser (Fig. 4) than filament networks found in TA2, which are thinner, overlap more tightly, and show 20-micron nodular ends (Fig. 6). In TA3, the distribution of positive and negative ions outlines an elliptic microstructure that is perpendicular to straight laminar projections (Fig. 7). Group 1 shows maximum intensities in different molecular groups, including a series of N-bearing compounds (e.g., *m/z* peaks at 268.30 and 282.31 corresponding to C_18_H_38_N^+^ and C_19_H_40_N^+^) and NH_4_^+^-bearing positive adducts found at 296.33, 494.56, 522.59 and 550.62 fitting C_20_H_42_N^+^, C_34_H_72_N^+^, C_36_H_76_N^+^, and C_38_H_80_N^+^, respectively. This group is also followed by a series of negative ions identified as fatty acids and/or ester carboxylates (e.g., *m/z* 197.15, 199.17, 227.20, or 255.23, corresponding to C_12_H_21_O_2_^-^, C_12_H_23_O_2_^-^, C_14_H_27_O_2_^-^, or C_16_H_31_O_2_^-^, respectively) and those bearing SO_3_^-^ and SO_4_^-^ adducts such as C_8_H_7_SO_3_^-^ (*m/z* 183.01), C_12_H_25_SO_4_^-^ (*m/z* 265.15), C_14_H_29_SO_4_^-^ (*m/z* 293.17), C_19_H_31_SO_3_^-^ (339.19) and C_27_H_45_SO_4_^-^ (465.30). As discussed in the ToF–SIMS spectral characterization, the fragmentation pattern of positive and negative ions in Group 1 suggests that the group results from sulfate mineralization on a lipidic surface composed of sphingolipids (Fig. S6; Table S2).

***Group 2*** A couplet of parallel linear elements (Figs. 4a) with an average thickness of > 80 microns is not associated with any structure defined by inorganic ions (Figs. S2–S3). In negative ions, the average morphology varies in thicker microstructures with internal triangle-like patterns and external irregular boundaries (Fig. 4b; Fig. S7). In some cases, the internal microstructure defined by the negative ions shows a more complex design that partially resembles the Group 1 pattern in TA1 (e.g., *m/z*^-^ peak at 58.01; see Fig. S7a). Furthermore, in TA3, the anions defining Group 1 occur in the mineral matrix as a negative cast of the main structure (Fig. 7). The group shows a maximum in positive peaks at *m/z* 31.02, 47.01, 61.03, 69.03, 85.03, and 87.04 corresponding to cations CH_3_O^+^, CH_3_O_2_^+^, C_2_H_5_O_2_^+^, C_4_H_5_O^+^, C_4_H_5_O_2_^+^, and C_4_H_7_O_2_^+^, respectively. At the same time, different organic anions have been found to be associated with peaks at *m/z* 43.02, 53.00, 55.02, 58.01, 59.02, 67.02, 69.03, 71.02, 83.01, 85.03, 87.01, 99.01, and 101.03 (Fig. S7b), which reflect anions C_2_H_3_O^-^, C_3_HO^-^, C_2_H_2_O_2_^-^, C_2_H_3_O_2_^-^, C_4_H_3_O^-^, C_4_H_5_O^-^, C_3_H_3_O_2_^-^, C_4_H_3_O_2_^-^, C_4_H_5_O_2_^-^, C_3_H_3_O_3_^-^, C_4_H_3_O_3_^-^, and C_4_H_5_O_3_^-^, respectively.

*Group 3* This group is a set of different ovoid microstructures averaging 60 microns. In TA1, the positive ions define nodular microelements that follow a similar orientation (Figs. 4 and 5). In contrast, the negative ions outline a structure following a square-like shape limited by < 40-micron filamentous elements (Figs. 4 and 5). In TA2, the positive and negative ions outline nodulous rows that are closely parallel to the matrix fabric (Fig. 6) and are characterized by a sharp boundary separating two subzones with different microtextures (Fig. S3). In TA3, positive peaks show the same pattern as observed in Group 2, which has a maximum in the rock matrix, leaving the main microtexture as an empty cast (Fig. 7). The area is defined by *m/z* peaks at 28.03, 42.04, 43.06, 44.05, 56.06, 84.08, 86.07, 86.10, 110.08, 111.10, 112.09, 122.08, 123.09, 124.09, 125.11, 126.11, 135.09, 136.09, 137.11, 138.11, 308.31, and 310.33, which match CH_2_N^+^, C_2_H_4_N^+^, C_3_H_6_^+^, C_2_H_6_N^+^, C_4_H_8_^+^/C_3_H_6_N^+^, C_5_H_10_N^+^, C_3_H_8_N_3_^+^, C_5_H_12_N^+^, C_6_H_10_N_2_^+^, C_6_H_11_N_2_^+^, C_5_H_10_N_3_^+^, C_6_H_8_N_3_^+^, C_7_H_11_N_2_^+^, C_6_H_10_N_3_^+^, C_7_H_13_N_2_^+^, C_7_H_14_N_2_^+^, C_8_H_11_N_2_^+^, C_7_H_10_N_3_^+^, C_8_H_13_N_2_^+^, C_8_H_14_N_2_^+^, C_20_H_38_NO^+^, and C_20_H_40_NO^+^, respectively. Furthermore, a set of different negative ions tracing the microstructure in Group 3 are found at *m/z* 91.02, 92.02, 93.04, 94.03, 117.03, 211.06, and 212.07, which match well with molecular fragments C_4_HN_3_^-^, C_4_H_2_N_3_^-^, C_5_H_5_N_2_^-^, C_4_H_4_N_3_^-^, C_4_H_5_O_4_^-^, C_13_H_9_NO_2_^-^, and C_13_H_10_NO_2_^-^, respectively.

*Group 4* This group is characterized by positive ions at 30.04, 178.07, 179.09, 180.05, 209.08, 211.10, and 219.18, which fit the fragments CH_4_N^+^, C_9_H_8_NO_3_^+^, C_8_H_9_N_3_O_2_^+^, C_12_H_6_NO^+^, C_14_H_11_NO^+^, C_13_H_11_N_2_O^+^, and C_11_H_25_NO_3_^+^, respectively. TA1 is composed of two coupled elongated nodular microstructures in which the elongation axes connect both nodule centers (Fig. 4). One of the micronodules fits well with the distribution of a couple of micronodules found in Group 3. At the same time, the occurrence of the second micronodule complements the microelements of Groups 3 and 6 (Figs. 4 and 5). This group corresponds to Group 3 of TA2 (Fig. 6), suggesting that it results from spatial variations in the molecular composition of organic N-bearing compounds with the same structure preserved (Fig. 5). In TA3, the group shows a pattern similar to that observed in Groups 2 and 3, which outline the main microstructure preserved as an empty cast (Fig. 7).

*Group 5* This group is characterized by a 600 micron-long elongated triangle-like morphology crosscutting the main structure fabric of Groups 1 and 2 (Figs. 4 and 5). While the molecular fragments in TA2 are not associated with any specific morphology, in TA3, they follow the same pattern as observed in Groups 2 to 4, which outline the main microstructure as an empty cast. In fact, Group 5 is characterized by positive ions at *m/z* 76.03, 77.04, 104.03, 105.03, 149.02, and 150.03 (C_6_H_4_^+^, C_6_H_5_^+^, C_7_H_4_O^+^, C_7_H_5_O^+^, C_8_H_5_O_3_^+^, and C_8_H_6_O_3_^+^) and negative ions at 73.03, 77.04, 105.04, 120.02, and 121.03 (C_3_H_5_O_2_^-^, C_6_H_5_^-^, C_7_H_5_O^-^, C_7_H_4_O_2_^-^, and C_7_H_5_O_2_^-^, respectively). Such a distribution of fragments is consistent with the occurrence of piperonal derivatives that are common in phytochemicals^56^.

*Group 6* Three 120-micron subcircular morphologies show an empty central void. Such subcircular microstructures are equidistant from each other at a distance of ~ 350 microns in TA1 (Fig. 4). The inner empty area of each edge is filled by ions of Groups 3 and 4 (Fig. 5). While there is no morphology associated with this group in TA2, the area TA3 follows the same pattern as observed in Groups 2 to 5 (Fig. 7). In fact, Group 6 is defined only by three positive ions at *m/z* 166.04 and 168.04, matching C_5_H_10_O_6_^+^ and C_8_H_8_O_4_^+^, which could correspond to the fragmentation of vanillic acid (C_8_H_8_O_4_) derivatives^57^.

*Group 7* The occurrence of 10- to 50-micron micronodules follows the main pattern appearing in Groups 3 and 4 (Fig. 4). This group is found in only TA1 through the positive ion at 57.04 (C_3_H_5_O^+^), as well as by negative fragments at 279.16 and 280.16 (C_16_H_23_O_4_^-^, and C_16_H_24_O_4_^-^). The positive and negative ion fragmentation pattern suggests that the source compound is a terpenoid-like structure occurring in different plant tissues, such as dioic acids found in the cuticular wax or terpene-like fungal metabolites^58^ (e.g., pestaloporonin derivatives).

*Group 8* In this group, microstructures characterized by a few negative ions arranged in two sets (at *m/z* 666.06, 667.07, 668.07, 736.14, and 737.15) occur only in TA1 (Fig. 4). Such fragments outline structures with short branches with an average length of 200 microns that are transverse to the fabric of Group 1 (Fig. 5). Thus, the anion mass fits different fragments with high oxygen moieties (i.e., C_27_H_22_O_20_^-^, C_27_H_23_O_20_^-^, C_27_H_24_O_20_^-^, C_32_H_32_O_20_^-^, and C_32_H_33_O_20_^-^) that could belong to biomolecules related to tannins (e.g., PubChem CID 131,834,675) or other polyphenols produced by fungi^59^.

*Group 9* This group is defined by many positive ions with masses greater than 200 Da such as 239.25, 281.07, 313.30, 367.32, 495.47, 523.50, 537.52, 565.53, 579.56, 593.58, and 649.63, which are assigned to fragmented glycerolipids^60^ such as C_16_H_31_O^+^, C_17_H_13_O_4_^+^, C_19_H_37_O_3_^+^, C_23_H_43_O_3_^+^, C_31_H_59_O_4_^+^, C_33_H_63_O_4_^+^, C_35_H_67_O_4_^+^, C_37_H_71_O_4_^+^, C_38_H_73_O_4_^+^, and C_42_H_81_O_4_^+^ and *m/z* anions such as 227.19, 241.22, 253.21, 255.22, and 269.23, which match well with fatty acid fragments or carboxylate esters such as C_14_H_27_O_2_^-^, C_15_H_29_O_2_^-^, C_16_H_29_O_2_^-^, C_16_H_31_O_2_^-^, and C_17_H_33_O_2_^-^, respectively. The group occurs as a densely packed network of 40-micron-long filament-like microstructures, which appear in TA2 along the sharp boundary that contacts Group 1 (Fig. 6; Fig. S3). The distribution of positive and negative ions defining Group 9 fits the primary microstructure found in TA3 (Fig. 7), while in the TA1 matrix, the positive ions of this group show a more homogeneous occurrence.

### ToF–SIMS spectral analysis: compound classification

The spectral analysis of the sample 010,109–1 TAs provides the identification of different biomolecular fragments and organic compounds. Although the spectral fragmentation patterns are very intricate as a result of secondary ionization between the C-bearing compounds and the Fe- and S-bearing mineral matrix, it is possible to identify diverse groups of organic compounds, which are characterized in detail in the Supplementary Information. In short, the ToF–SIMS spectral analysis identifies the following molecular groups.

**Figure 8:**
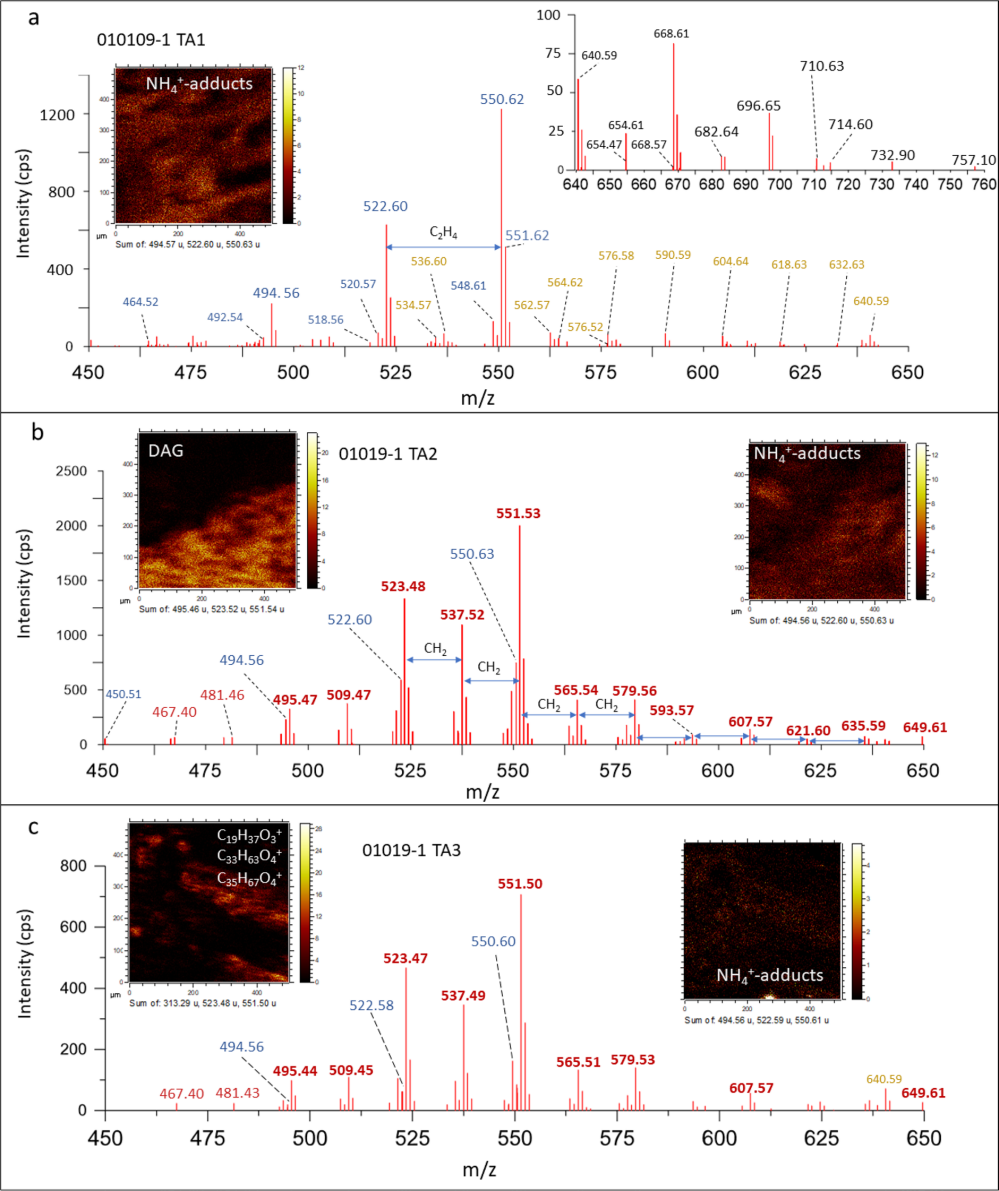
Detection of different biolipids by the fragmentation pattern in the spectrum range of *m/z* 450 to 650 Da in TA1 (**a**), TA2 (**b**), and TA3 (**c**). The peak distribution shows the occurrence of two different sets of fragments that include even versus odd masses corresponding to acylglycerides (red), NH_4_^+^-adducts (blue), and sphingolipid fragments (orange) (see Supplementary Tables 5 and 6). The ToF–SIMS molecular images show a high affinity between sphingolipid fragments and NH_4_^+^-adducts (**a**), suggesting that both are sourced from the same lipidic structures. Furthermore, the peak intensities of the different cations also support a change in the molecular composition for each target area. While TA1 (**a**) shows high intensity for NH_4_^+^-adducts (1200 cps at 550 Da), it is doubled by the acylglyceride intensity in TA2 (e.g., > 2000 cps at 551.53). TA3 (**c**) acylglyceride intensity is three times lower than that in TA2 (**c**). Such intensity and composition changes strongly suggest that TA2 (**b**) is the target area recording the highest biomass, which is dominated by acylglycerides, while sphingolipids would be the main compounds in TA1 (**a**) in terms of biomass. TA3 (**c**) appears as an intermediate area in terms of acylglycerides and sphingolipids which are mainly concentrated in the microbial structures (Fig. 7).

**Figure 9:**
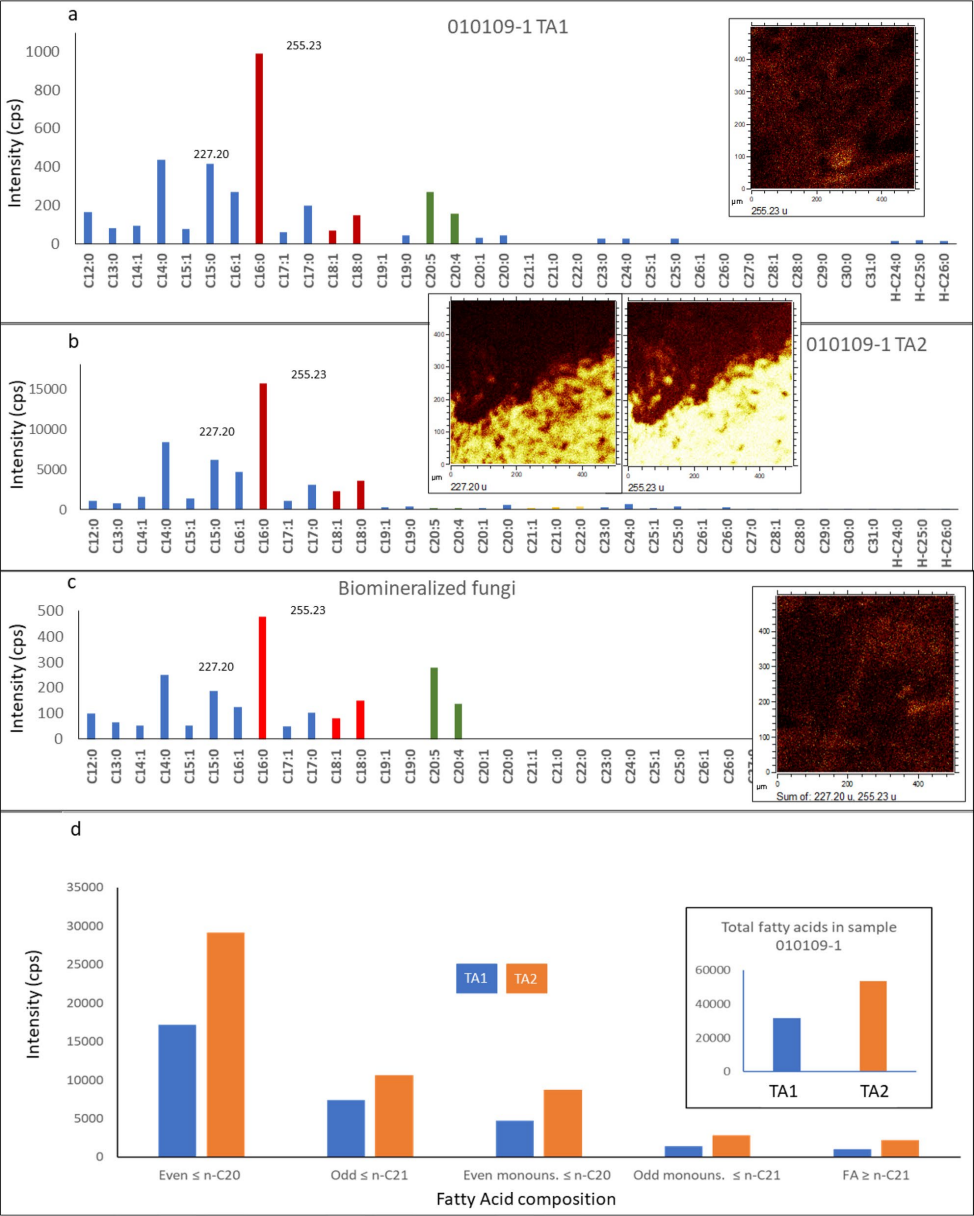
Occurrence and composition of fatty acids (FAs) through ToF–SIMS analysis in sample 010,109–1. The FA compositions found in TA1 (**a**) and TA2 (**b**) are very similar to the FA composition that was obtained in mineralized fungi (**c**) collected from microbial structures in Pyrenees mine galleries in which the biomass was dominated by fungal hyphae as reported by Fernandez-Remolar et al. (67). Interestingly, the highest FA intensity is found in TA2 rather than TA1 and the Pyrenean sample, which both show a similar intensity in FAs. Such a distribution strongly suggests different biological origins for TA2 versus TA1 and the mineralized fungi^66^. Indeed, PUFAs are shown as distinctive compounds in both TA1 and the Pyrenean sample. Furthermore, the account of the concentration of even versus odd FAs in TA1 and TA2 supports a microbial main source for FAs.

1. *n-Alkanes and other straight hydrocarbons with low degrees of unsaturation* The occurrence of saturated and monounsaturated straight chain hydrocarbons (i.e., *n*-alkanes and alkenes) is supported by the detection of low mass peaks (< 120 Da). The peak intensity and distribution of the two sets of ions suggest that alkenes are the dominant forms among the straight hydrocarbons. This feature implies the presence of abundant large lipid compounds in the Upper Gossan unit^61^ that should be composed of saturated and monounsaturated chains. The TA cation fragmentation pattern also occurs as straight hydrocarbons that form NH_4_^+^-bearing adducts^62^ (Fig. S6). The fragment composition and distribution strongly suggest that the N-bearing compounds and adducts are produced by the ionization of a matrix enriched in different sphingolipids, which are associated with filamentous fungal structures^45,63,64^ (Fig. 8).
2. *Branched hydrocarbons and isoprenoids* The occurrence of high-intensity peaks at 43.06 and 57.07 is also consistent with the preservation of different branched hydrocarbons, including isoprenoids, that are detected using GC–MS (Table S1).
3. *Polycyclic aromatic hydrocarbons (PAHs)* A set of TA1 and TA3 peaks suggests the occurrence of PAHs^65,66^ (Table S3). The PAH cation distribution is homogeneous in the mineral matrix (Fig. 7), suggesting that it was produced during organic mineralization in the Upper Gossan unit. Such a process resulted from the combination of fungal and microbial degradation of cellulose, lignin, pectin, and other polysaccharides^67,68^, as well as other sources such as woodland fires^69^.
4. *Fatty acids* The mass spectra of negative fragments obtained through ToF–SIMS record a diverse set of fatty acids (FAs) ranging from C_11_ to C_30_ (Fig. 9). These FAs include saturated, unsaturated, and hydroxylated compounds (Table S4), the distribution of which follows the TA fabric (Figs. 4, 6, and 7; Figs. S2 to S4). A rough estimation of FA abundance shows a higher concentration of fatty acids in TA2 than in TA1, agreeing with a higher concentration in glycerolipids^70^ (Fig. 6). The anion intensity distribution shows that the concentration of FA chains with fewer than 22 carbon atoms is higher than that of > *n*-C_21:0_ FAs (Fig. 9). The total sum of the [M–H]^-^ anion intensity shows that in TA1 and TA2, the saturated and monounsaturated ≤ *n*-C_20_ FAs have a higher intensity than the > *n*-C_20_ FAs, while the total intensity even FAs exceeds that of odd FAs in ≤ *n*-C_20_ chains (Fig. 9). Such a distribution pattern is also observed in the spectral data obtained from mineralized fungi that are used as a reference for FA composition^71^.
  The mineralized fungal reference (Fig. 9) shows the occurrence of ≤ *n*-C_18_ saturated and monounsaturated chains, while saturated and monounsaturated FAs longer than 18 carbon atoms have negligible concentrations. In the ferruginized fungi^71^, the ≤ *n*-C_20_ even fraction exceeds approximately three times the total intensity of ≤ *n*-C_20_ odd FAs, as is also observed in TA1 and TA2 (Fig. 9). The ToF-SIMS spectral data also show the occurrence of polyunsaturated fatty acids (PUFAs), such as C_20:5_ and C_20:4_, corresponding to eicosapentaenoic and arachidonic acids (Fig. 9; Table S4). The TA1 and TA2 fatty acid distributions suggest that the record of organic compounds in the Upper Gossan unit has resulted in intermixing from heterogeneous biological sources, including microbial communities and higher plants^72,73^.
  However, both even and odd FAs found in sample 010109-1 are associated with fungal and nonfungal microbial structures (see Fig. 4, 5, 6, and 7), which suggests that in the Rio Tinto subsurface, FAs are not mainly sourced from higher plants, although higher plants can be a secondary source. The occurrence of C_20:5_ and C_20:4_ PUFAs has been reported in different fungi^72,74^, as has been found in different higher plants. This is also the case for the long-chain hydroxylated FAs (≥ C_24_) considered molecular markers of vascular plants derived from suberin^75,76^. Alternatively, they are also found in fungi^77^ that are produced through the enzymatic oxidation of alcohols (e.g., lipoxygenase) and other FAs^78^.

5. *Glycerolipids and glycerophospholipids* A diverse set of odd cations occurring in a wide range of masses > 300 Da agrees with the fragmentation pattern of different glycerolipids, including monoacylglycerides (MAGs), diacylglicerids (DAGs), and glycerophospholipids (GPLs) (Figs. S8, S9, S10, and S11; Table S5)^60,70^. The detection of diglyceride structures is additionally supported by the occurrence of different carbonyl-bearing fragments^79^ (Table S5). Furthermore, different peaks found in TA1 to TA3 match cations that are well known as fragments of head groups for choline and phosphatidylcholine^79^. The presence of glycerophospholipids is supported by different peaks that occur at higher intensity in TA2^79^.
6. *Sphingolipids (ceramides)* The presence of different even peaks in TA1, TA2 and TA3 (Figs. 4, 5, 6, and 7) can be ascribed to fragmented sphingolipid backbone chains^80^ such as C_20_H_40_NO^+^. These chains can be produced through two main dehydration pathways^80^, namely, [M + H—H_2_O]^+^ and [M + H—2H_2_O]^+^, on sphingenine (C_18_H_37_NO_2_), sphinganine (C_18_H_39_NO_2_), aminomethyl-nonadecane-triol (C_20_H_41_NO_3_), and phytosphingosine (C_20_H_43_NO_3_) (Table S6)^81^. Furthermore, TA1, TA2 and TA3 show dozens of high even peaks (> 500 Da) that fit the fractionation of different ceramides well. The detection of diverse N-bearing cations with 1 to 6 oxygen atoms suggests the presence of different types of sphingolipids with chain lengths greater than C_38_. The mass distribution of positive ions above 530 Da agrees with the preservation of diverse ceramides, including cerebrosides and sphingomyelins^80^. Although the identification of different ceramide fragments with the same chain length but the number of oxygens varying between 1 and 6 is consistent with different dehydration pathways in the ionization process^80^, these fragments could also originate from the loss of ceramide hydroxyls during early mineralization. Furthermore, the decrease in oxygen and carbon numbers could also result from fragmentation of the glycosidic head in cerebrosides during the ionization process^80,82^ (Fig. S12). However, microbial degradation cannot be excluded as a cause for the loss of glycosidic groups.
7. *Peptide fragments and amino acids* TA1, TA2 and TA3 have positive and negative peaks (< 150 Da) that match well with a diverse series of N-bearing fragments (Table S7)^83^. They could correspond to fragments of multiple amino acids (e.g., C_3_H_6_N^+^, C_5_H_10_N^+^, and C_5_H_12_N^+^) and specific fragments for some amino acids, including lysine (C_5_H_10_N^+^), asparagine (C_3_H_7_N_2_O^+^), and aspartic acid (C_4_H_5_O_4_^-^)^84,85^. The potential preservation of protein fragments and amino acids in the oldest deposits of the acidic Rio Tinto basin, such as the Upper Gossan unit, is suggested by the detection of peptidic chains in the 2.1-Ma Rio Tinto terrace^26^.
8. *Steroids and hopanoids* Sample 010,109–1 shows *m/z* peaks that are characteristic of different cyclic triterpenoids, such as steroids. However, some of the diagnostic cations either have weak peaks or occur as shoulders on the sides of larger peaks, which prevents characterizing compounds at the level that has been achieved by GC–MS (Table S1). Diagnostic fragment masses for steroids^86^ are found (Table S8) as weak peaks at 149.13 (C_11_H_17_^+^), 191.18 (C_14_H_23_^+^), 203.18 (C_15_H_23_^+^), and 257.22 (C_19_H_29_^+^). Masses assigned to ions such as 367.37, 383.34, and 402.37, which fit C_27_H_43_^+^, C_27_H_43_O^+^, and C_27_H_48_NO^+^, respectively, suggest the presence of C_27_ steroids (e.g., C_27_H_44_O) and cholesterol derivatives. Cholesterol and likely cholesteryl sulfate (C_27_H_46_O and C_27_H_46_SO_4_) are identified through their main diagnostic peaks (Table S8). The occurrence of such SO_4_-bearing lipids is additionally supported by a set of diverse negative ions (Table S8). The higher peak intensity of cholesteryl sulfate than of cholesterol suggests that sulfate-bearing lipids are more abundant in TA1, while cholesterol is relatively more abundant in TA2. Several additional weak peaks could correspond to cholestanol, dehydrocholesterol, stigmasterol, campesterol, or ergosterol ions (Table S8) that are detected by GC–MS analysis (Table S1). The detection of different peaks as fragments of different sterols shows that eukaryotic organisms such as fungi and plants were the main contributors to the gossan biolipid inventory. However, the most abundant sterol in terms of intensity, which is cholesteryl sulfate, suggests that the mineralization process driven by enriched sulfate solution produced secondary organic complexes composed of lipid structures bonded to inorganic compounds.
9. *Polyphenols, lignin derivatives and other phytochemicals* The ion mass distribution of some distinctive cation and anion peaks defining Groups 5 and 6 (Figs. 4, 5, 6, and 7) suggests the occurrence of compounds derived from phytochemicals such as terpenoids and polyphenols (Table S9). This collection of ions resembles a series of incomplete fragmentation of degraded oligomeric units that are differentiated by the number of oxygen atoms and the methyl groups. The occurrence of different sets of ions partly matches the fragmentation series of vanillin (C_9_H_10_O_3_) or cinnamic acid (C_10_H_12_O_3_) derivatives^87–89^ that result from the decomposition of lignin by different microbial agents. The peak distribution in both sets of positive and negative ions could also be interpreted as a few preserved phytochemical terpenoids from aromatic plants, such as derivatives of heliotropin or piperonal (C_8_H_6_O_3_). These phytochemicals are currently metabolized and produced by soil bacteria^56^. In this instance, the distribution of the two sets of different ions could represent variation in the phytochemical composition of a plant residue.

**Figure 10:**
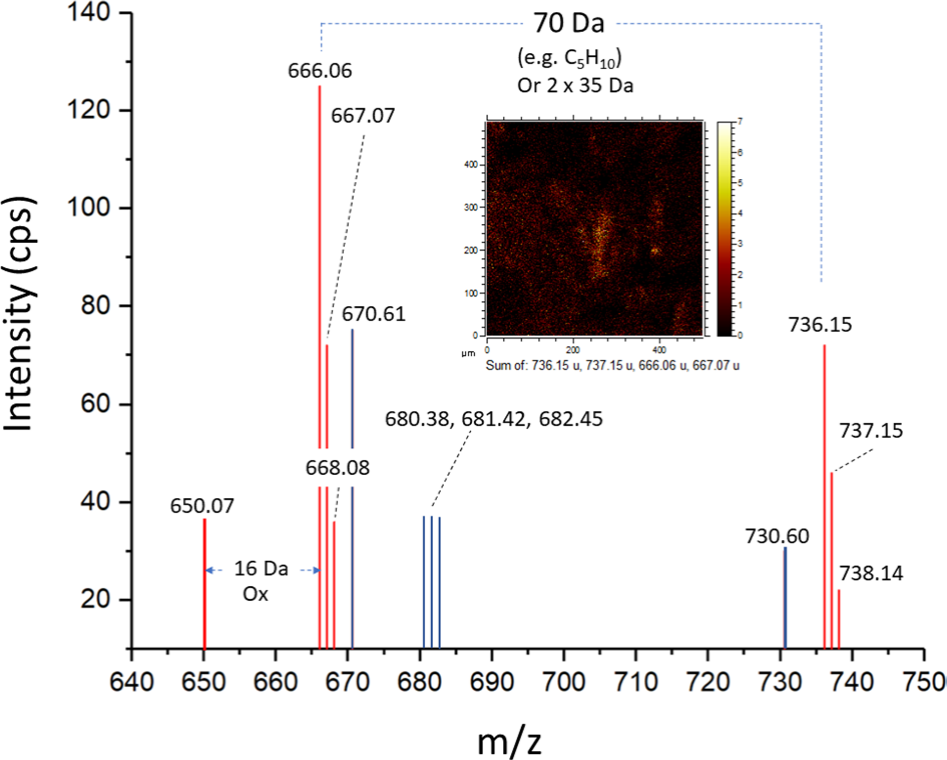
Fragmentation pattern of unknown compounds defining G8. The distribution of fragment masses suggests the presence of biological compounds with high contents of oxygen and/or nitrogen, which correspond with polysaccharides and polyketides.

Furthermore, the spectral analysis provides a set of negative ions of unknown origin occurring at *m/z* 650.07, 666.06, 668.08, 736.15, 737.15, and 738.14 that occur in only TA1 as Group 8 (Figs. 4, 5, 6, 7, 8, 9, and 10). This suggests the presence of compounds with a high number of O and/or N, which is compatible with the preservation of larger fragments of polysaccharides or polyketides.

## Discussion

The analysis of the Upper Gossan sample through the different spectral approaches provides complementary evidence of the high degree of preservation of the acidic and oxic ancient deposits. The analysis of high-resolution FTICR-MS results yields a data source to recognize the occurrence of different major groups of biomolecules, including peptide-like and oxygen-rich compounds (Fig. 3), which are signs of a well-preserved molecular association^90^. The ToF–SIMS imaging analysis also suggests that the molecular association results from the combination of microbial activity and the presence of phytochemicals. Such results are accompanied by high chemodiversity and abundant molecular information, which is constrained by the extractive technique (Fig. S13) to masses lower than < 600 Da. However, the ToF–SIMS analysis shows that the molecular weights of the compounds in the Upper Gossan sample 010,109–1 are much higher than 1,000 Da, suggesting that the preservation of the biological materials was remarkably high.

The GC–MS analysis of solvent-extractable lipids from the gossan sample provides many compounds, including a very diverse set of FAs, hydrocarbons, isoprenoids, alkanols, hopanoids and steroids (Table S1). As previously described in the GC–MS results, FAs consist of an association of very different compounds, including straight chains with even-over-odd dominance (from 11:0 to 30:0), branched FAs (including *iso-*/*anteiso-* pairs from 13:0 to 25:0), monounsaturated FAs (16:1 ω7, 18:1 ω9, 18:1 ω10, and 20:1 ω8) and minor HMW hydroxyl FAs (24:0, 25:0, and 26:0). The triterpenoid structures have different cholesterol derivatives that concur with other triterpenoid structures, such as β-sitosterol, stigmasterol, ergostanol, lanostanol, and lupanol (Table GC–MS). Such a triterpenoid association likely resulted from a very heterogeneous source that included different eukaryotic forms, such as plantae, animalia, and fungi^51,52,91^. Therefore, the biological information recovered by the GC–MS analysis of the gossan sample strongly supports organic production from multiple sources that include aquatic (i.e., macrophytes) and land plants, bacteria, algae/phytoplankton and fungi.

Both FTICR-MS and GC–MS results are highlighted by the ToF–SIMS imaging capabilities, which have allowed the characterization of microbial communities through the distribution of distinctive biomolecular fragments. Such distributions distinguish different imaging groups (from 1 to 9), whose morphologies suggest that they correspond to parts of living organisms, microbial communities and/or biofilms^92^. These morphologies are observed in the molecular imaging of the different target areas that captures the occurrence of several biological structures. In fact, the G1 group in TA1 (Fig. 4) shows thick filamentous structures with a high content of N-bearing positive fragments that can be attributed to sphingolipids. The ToF–SIMS spectral analysis of TA1 also shows high enrichments in different sets of positive ions that have been assigned to plain ceramides and glucosylceramides (e.g., cerebrosides and sphingomyelins). As such, an association of biomolecules is currently found in microbial fungi^82,93^ that occur as filament clusters (hyphae) displaying a magnitude comparable to that of the filamentous structures defined by N-bearing fragments as G1 (Fig. 4). The filamentous structures are also well defined by high intensities of different inorganic SO_n (2 ≤ n ≤ 4)_ anions and organic sulfate adducts, which strongly suggests hyphae biomineralization, as commonly occurs in some modern Rio Tinto fungi^14^. The detection of high contents of sulfate adducts likely results from the binding between SO_4_^2^ and HSO_4_^-^ anions with a positively charged fungal hyphae surface, which is partly formed by lipids. In fact, the occurrence of cholesteryl sulfate defined by a large peak (I > 400 to 1050 cps) at *m/z* 465.30 in the different target areas (Figs. 4, 5, and 6) strongly suggests that the sulfate organic adducts result from the ionization of the biomineralized fungal surface by sulfate anions. Organic sulfate-bearing adducts are not found in biological structures other than the filamentous fungal structures, suggesting that the sulfate biomineralization is highly specific to some fungal microorganisms^14^.

As discussed above, the different biological structures defined by morphological groups 1 to 9 are emphasized by the highly distinctive molecular composition distribution. The red/green/blue (RGB) overlay of the different morphological groups (Figs. 5, 6, and 7) shows that most of the biological structures interlock with each other to form larger biological assemblages. Although the origin of some of the preserved structures is unknown, the morphology and composition of most of them strongly suggest that they correspond to an association of different fungal structures (e.g., G1, G3 and G7) that are associated with parts of other living forms, such as plants represented in both Groups 5 and 6 (Figs. 4 and 5). As shown in the results, G1, G3 and G7 are formed by N-bearing fragments and sulfur-bearing adducts sourced in sphingolipids and biomineralized bodies by sulfate (Fig. 2; Table S2), which corresponds to the preservation of fungal communities^14,80,93^, while G5 and G6 are defined by different ions fitting the fragmentation pattern of some phytochemicals, such as vanillic acid and piperonal^56,57^. In terms of biomass, the main fungal structure corresponds to G1, which is characterized by dozens of fragments (Table S2) that usually have a high intensity as well. This feature is very common in the different N-bearing *m/z* cations that are characterized as sphingolipid fragments and secondary anions (e.g., G8) that could have been produced by the fragmentation of large polysaccharides forming fungal biofilms^94^.

The occurrence of exceptionally preserved biofilms with two different microbial forms is observed in TA2 (Figs. 6 and 8; Figs. S10 and S11), where two different biological structures are found. They are distinguished by the molecular composition that defines G1, which is enriched in ceramides and sulfur adducts, versus G9, which is characterized by a high abundance of diacylglycerides, through a sharp boundary between the two groups (Fig. 6). G3 interlocks G1 (Fig. 6) in the same way as in TA1, which suggests that there are compositional changes in the fungal biofilm. Furthermore, in TA3, ToF–SIMS image analysis shows additional biological structures whose morphology and molecular composition suggest a fungal origin but also contain glycerolipids in addition to sphingolipids and SO_4_-mineralized compounds (Fig. 7). As discussed above, the fungal structures defined in TA1 and TA2 show high concentrations of sphingolipids and SO_4_-mineralized compounds but depletion in glycerolipids (Figs. 4 and 6), which suggests that the biological structure found in TA3 corresponds to a different biological origin. The phytochemicals (e.g., vanillic acid and piperonal derivatives) that characterize some biological structures in TA1, such as G5 and G6 (Fig. 4), occur in TA3 within the mineral matrix in which the biological structures are embedded (Figs. 1 and 7). Such a distribution suggests that they were transported in the acidic solution after being released from plant fragments in more proximal and/or surficial areas where the plants were growing. They come together with a set of different PAHs (Fig. 7; Table S3), which are recalcitrant compounds resulting from the photochemical process and are common in continental waters. This agrees with the parallel arrangement of the fungal structures (Fig. 1), which suggests that they were oriented along an acidic flow with a high concentration in ferric oxysulfate polymers^95^.

Indeed, the ToF–SIMS image analysis provides enough evidence in the form of chemical compositions to gain insight into the mineralization processes that led to the preservation of biological structures and species. This preservation was accomplished by means of different mineralizing episodes that started with sulfate biomineralization by the activity of some fungal and bacterial communities^14^. Sulfate biomineralization was followed by ferric-bearing mineralization, whose composition could have been mediated through different phases, including schwertmannite, hydronium jarosite, and nanophase goethite^23^. The mineralization in this case was a passive process in which biological structures were coated and infilled with ferric-bearing compounds whose composition was controlled by the solution hydrochemistry. The rock maturation ended in partial replacement of the primary phases by goethite and hematite (Fig. 2). Such limited maturation is evidenced by high concentrations of sulfate and organic carbon, which were determined by ToF–SIMS analysis (Figs. 4, 5, 6, and 7). In this context, the precipitation of the ferric and sulfate phases played an essential role in the preservation of the structure and composition of the biological elements in three ways: (1) building rigid mineralized structures resistant to sediment load or tectonic stress and chemical attack^96,97^, (2) limiting oxygen availability and thus inhibiting the degradation of biological compounds by a high concentration of cations^98^ that are very harmful for microbial life^99^, and (3) a fast decrease in water activity during mineral precipitation^100,101^.

## Conclusions

The spectral and ToF–SIMS image analyses of the Upper Gossan sample show that hyperacidic and oxic conditions leading to ferric deposits have remarkable preservation potential in terms of structure and composition. Such a preservation potential is also supported by the occurrence of peptidic chains in 6- to 2.2-Ma Rio Tinto terrace deposits^26^. The same low-pH and oxidizing conditions have been hypothesized for some regions on Mars during the Noachian-Hesperian transition^6,7^. In this period, different mineral phases, such as jarosite, schwertmannite, nanophase goethite, and other unknown Fe^2+^/Fe^3+^-bearing sulfate or oxide phases^9,102,103^, could have played essential roles in the mineralization of microbial remains if they were ever produced on Mars. The precipitation of ferric phases has been reported to follow fast saturation^7^, which is observed in the Rio Tinto Mars analog. The saturation of acidic solutions on Mars would have increased the concentrations of different cations, including toxic metals^98,99^, and decreased the water activity^100^, both of which greatly inhibit microbial activity. Indeed, the water activity decreases with temperature^104^, so the net preservation of biomolecular compounds would be greatly favored at the low temperatures on Mars. The deposit maturation to goethite and hematite would confer on them a higher resistance to deformation and weathering. High concentrations of ferric ions in rock and solutions^105^ would also have provided protection against UV radiation once the microbial remains were eventually produced on Mars.

Although preservation in clay-rich deposits is currently associated with organic preservation^21^, the preservation potential can be greatly reduced when clay deposits are exposed to different maturation and alteration processes that can degrade organic compounds in later stages of rock diagenesis on Mars, such as those associated with enhanced weathering (Fig. S14), which likely occurred during the Noachian to Hesperian transition. The solutions involved in clay formation would have not had high concentrations of cations that could eventually mineralize the biological structures and protect them against UV radiation^105^ and lithostatic compression^97^. When mineralization does not occur, biomolecular compounds can be broken into smaller compounds by lithostatic compression. Indeed, such mild environments are associated with unfavorable mineralizing conditions, which implies low potential for preserving structures. Assuming that Mars was exposed to acidic episodes, there is a high possibility that if life emerged on the planet, its molecular preservation might have followed the same mechanisms to record traces of ancient life on the surface and in the interior of the planet. In fact, the search for life on Mars could be expanded to large and extensive acidic deposits formed billions of years ago.

## Material and methods

### Sample collection

A field campaign was conducted for surface sampling of the upper part of the Cenozoic gossan^13^ topping the Peña de Hierro Carboniferous basement (Fig. 1a). This unit corresponds with the oldest alteration episode that formed the Upper Gossan materials through sulfide weathering by a water table that remained relatively stable during the late Oligocene-early Miocene^27^.

### Sample 010109–1

was collected in Peña de Hierro (GPS coordinates 37.724918°, 6.553717°) from the uppermost part of the iron oxide deposits corresponding to the Upper Gossan unit aged late Oligocene^27^. The collected materials consist of massive iron oxide strata-like deposits forming ~ 1-m-thick lenses that are associated with the central and highest part of the gossan (Fig. 1a). Such materials currently vary downward or laterally to brecciated or tuffaceous oxide deposits (Fig. 1b–c), showing that the gossan formation involves a complex interplay between surface and subsurface processes. In this regard, such massive deposits are currently delimited at the top by efflorescent pseudomorphs and coated filamentous structures (Fig. 1c) that could result from a shallowing evolution from subsurface to surface conditions. The sample was collected directly from an outcrop showing few signs of alteration, and a geological hammer was used to prevent contamination that can be produced through drilling, which employs water as a coolant. The sample was carefully collected using nitrile gloves, covered by aluminum foil and introduced into a sterilized sampling bag to prevent contamination. Then, the sample was split into three parts; subsequently, each subsample surface was sterilized by igniting mass spectrometry-grade ethanol (sigma). Then, the three subsamples were delivered for spectrometry analysis. Observations under an optical microscope and a scanning electron microscope (SEM) of thin sections of the massive unit provided evidence for the occurrence of abundant biological fabrics that were templated by coating and mineralization (Fig. 1c). Furthermore, the sample observations under the different microscope techniques also evaluated the origin of the biological traces that were found in the rock matrix (Figs. S14 and S15). Interestingly, scanning electron and energy dispersive spectroscopy (SEM–EDS) analysis suggested very slight, if any, alteration of the mineral matrix of the sample. Indeed, the lack of different cations, such as Ca, Mg and K, and the low content of Si (Figs. S15 and S16) indicate that no alteration occurred^12^. In this regard, Fernandez-Remolar et al.^106^ reported the formation of silica laminations. Such laminations might have formed if the gossan material had been exposed to subsequent alteration associated with the occurrence of secondary biomolecules that were younger than the rock. Indeed, the SEM–EDS images show the presence of colomorphic goethite (Figs. S15 and S16) covered by laminated hematite, which is the result of long sedimentary maturation of the primary iron oxysulfates and/or oxyhydroxides^13,23^. Furthermore, the EDS analysis implies that goethite is associated with traces of S (see spectrum 3 in both Figs. S15 and S16) that likely came from a primary mineral composition of sulfates. Interestingly, SEM–EDS also targeted goethite with microstructures (Fig. S16) that were very similar to the filamentous microstructures shown in Figs. 4 and 5. The SEM–EDS image shows that the filamentous goethite is enclosed inside a hematite-rich lamina (Fig. S16), which indicates an old age and supports a primary origin for the preserved biological structures. Sample preparation. Sample analysis was performed using different state-of-the-art analytical techniques, including time of flight secondary ion mass spectrometry (ToF–SIMS) and Fourier transform ion cyclotron resonance mass spectrometry (FTICR-MS)^26,36^. These methods were supported by the more conventional technique of gas chromatography coupled with mass spectrometry (GC–MS), all of which have access to molecular databases with many compounds. In this regard, GC–MS is a technique that provides essential information to confirm compound identification. The preparation for the sample analyses using these three distinctive spectrometry techniques followed two different processes: simple thin sectioning and organic extraction from powdered samples.

Thin sections of sample 010109–1 were prepared for mineral and textural analysis under an optical microscope and a Scanning Electron Microscope (SEM) coupled with an Electron Dispersive Spectroscopy (EDS) probe, and molecular surface analysis by ToF-SIMS^107^. They were polished an extra time by using a 0.3 μm alumina paste to reduce surface imperfections that produce frequent interference during surface analysis. On the other hand, as FTICR-MS and GC–MS analyses of compounds require organic extraction, sample 010,109–1 was initially crushed under sterile conditions to prevent contamination.

### Microscope analysis

The inner structure of sample thin sections was observed under an upright trinocular microscope Motic BA400 coupled to a MoticamPro 282a camera. Optical observations were complemented with backscattered electron imagery using a Jeol JSM-5600 LV Scanning Electron Microscope (SEM). In addition, semiquantitative chemical microanalysis was performed with a Cambridge INCAx SIGHT EDS (Electron Dispersive Spectroscopy) to associate microstructure and mineral substrate with elemental composition.

### GC–MS and FTICR-MS analyses of organic extracts

Before lipid extraction, the sample was externally cleaned, and the outer layer was removed to avoid cross contamination with allochthonous material from the surface. In the first step, an external layer approximately 0.5 cm thick was removed with a solvent-cleaned (methanol and dichloromethane) saw and discarded. Subsequently, the remaining inner sample was externally cleaned with a solution of approximately 100 ml of hydroxide potassium (6% in Milli-Q water) while being gently shaken for 15 min in a glass beaker. After rinsing with Milli-Q water, the sample was then cleaned with nitric acid (6%) under the same conditions (shaking for 15 min). Finally, the sample was rinsed with Milli-Q water and ethanol (> 99.5% purity) and then dried in an oven (45 °C). Once dried, the sample was ground with a solvent-cleaned mortar and pestle for lipid analysis. Approximately 100 g of sample 010,109–1 was prepared for organic-solvent extraction for GC–MS analysis (Fig. S13). The extraction was performed with a mixture of dichloromethane/methanol (DCM/MeOH, 3:1, v/v) for 24 h with a Soxhlet apparatus. Internal standards (tetracosane-D_50_, myristic acid-D_27_, and 2-hexadecanol) were added prior to the extraction. The total lipid extract was concentrated by rotary evaporation to approximately 2 ml. After this process, activated copper was added, and the sample was allowed to stand overnight for elemental sulfur removal. The extract was separated into three fractions of different polarities (nonpolar, polar, and acidic). In the first step, a Bond-elute column (bond phase NH_2_, 500 mg, 40 µm particle size) was used to separate the neutral and acidic fractions by eluting first with 15 ml DCM/2-propanol (2:1, v/v) and subsequently with 15 ml diethyl ether with 2% acetic acid. In the second step, the neutral fraction was further separated into nonpolar and polar fractions by using an Al_2_O_3_ column (activated, neutral, 0.05–0.15 mm particle size) and eluting with 5 ml of hexane/DCM (9:1, v/v) and with 4 ml of DCM/methanol (1:1, v/v), respectively. The acidic fraction was derivatized with BF_3_ in methanol, and the polar fraction was derivatized with N,O-bis(trimethylsilyl)trifluoroacetamide (BSTFA). Together with the sample, a blank was extracted (i.e., an empty cellulose thimble), concentrated, fractionated and analyzed in parallel as a negative control. All glassware and sorbents were heated in a furnace at 550 °C for 24 h. All solvents were provided by Sigma Aldrich (Spain) and were of analytical grade (> 99.5% purity).

GC–MS analysis was performed to characterize the different nonpolar, acidic, and polar fractions. This analysis used a 6850 GC system and a 5975 VL MSD with a triple axis detector (Agilent Technologies). The recovery of spectral data was obtained using full scan mode (*m/z* 50 to 650) that operated with electron ionization at 70 eV. The analytes were injected (1 µl) into an HP-5MS column (30 m × 0.25 mm i.d. × 0.25 µm film thickness) with He as a carrier gas (1.1 ml min^-1^). For the nonpolar fraction, the oven temperature was programmed from 50 to 130 °C at 20 °C min^−1^ and then to 300 °C at 6 °C min^-1^ (held for 20 min). For the acidic fraction, the oven temperature was programmed from 70 to 130 °C at 20 °C min^−1^ and then to 300 °C at 10 °C min^−1^ (held for 10 min). For the polar fraction, the oven temperature program was the same as that for the acidic fraction, except that the oven was held for 15 min at 300 °C. The injector temperature was 290 °C, the transfer line was 300 °C, and the MS source was 240 °C. The identification of compounds was based on the comparison of retention time and mass spectra with the National Institute of Standards and Technology (NIST) mass spectral database and reference materials: *n*-alkanes (C_10_ to C_40_), fatty acid methyl esters (FAMEs; C_8_ to C_24_), *n*-alkanols (C_10_, C_14_, C_18_, and C_20_), and branched isoprenoids (2,6,10-trimethyl-docosane, crocetane, pristane, phytane, squalane, and squalene). All chemicals and standards were supplied by Sigma Aldrich and Chiron. The GC–MS data were acquired and processed by Agilent MSD ChemStation software, which performed molecular identification though NIST library matches of peak spectra.

To obtain the molecular information for sample 010,109–1, a solid-phase extraction was performed on the supernatant as described elsewhere^36,37^, and the extract was used for FTICR-MS analysis. This analysis was conducted with a SolariX FTICR mass spectrometer (Bruker Daltonik GmbH, Germany) equipped with a 12 T superconducting magnet (Magnex Scientific Inc., GB) and an APOLLO II electrospray ionization source (Bruker Daltonik GmbH) in negative ionization mode. The injection was performed using a microliter pump at a liquid flow rate of 120 ml h^-1^. Both sheath gas and curtain gas consisted of nitrogen. A source heater temperature of 200 °C was maintained to ensure rapid solvent evaporation in the ionized droplets. The FTICR mass spectrometer was precalibrated using known arginine clusters. The mass spectrum was zero filled to a processing size of four megawords using 750 scans within a mass range of *m/z* 147.4 to 1000 and internal calibration with an appropriate reference mass list, reaching accuracy values lower than 100 ppb. A blank sample was run before sample injection and did not show any clusters of contamination. Data treatment was performed using Compass Data Analysis 4.0 (Bruker Daltonics, Bremen, Germany). The exact masses of the detected *m/z* ions were defined, and their molecular formulas were calculated using a software tool written in-house^109^. The final formulas were branched into groups containing CHO, CHNO and CHOS molecular compositions, which were used to reconstruct the group-selective mass spectrum.

### ToF–SIMS analysis

ToF–SIMS is an analytic surface technique that characterizes the distribution of positive and negative ions in a sample. For this reason, it can provide essential information about the preservation of organic compounds, establishing a direct association between the mineral matrix and the occurrence of molecular biosignatures in the sample^108^. ToF–SIMS has a high detection level for different compounds, including contaminants that can potentially be attached to the thin section surface. To prevent this problem, the thin sections of sample 010109–1 were cleaned by surface sputtering with a 100 nA 500 eV oxygen ion beam for 3 s. The molecular surface analyses were performed by using a ToF–SIMS IV (ION-TOF, Münster, Germany) system under a pressure of 5·10^–9^ mbar. The thin section was sputtered with a pulsed bismuth liquid metal ion source (Bi_3_^2+^) at 50 keV. The ion beam was operated with a 20 ns pulse width and 0.3 pA pulsed ion current for a dosage lower than 5·10^9^ ions·cm^-2^, below the threshold level of the static SIMS conditions (1·10^11^ ions·cm^−2^). The released secondary ions were detected with a reflectron time-of-flight analyzer, a multichannel plate (MCP), and a time-to-digital converter (TDC). Charge neutralization was achieved with a low energy (20 eV) electron flood gun. Secondary ion spectra were obtained from a randomly rasterized square surface area with a 500-μm side within the thin section surface. Secondary ions were extracted with a 2 kV accelerating voltage and were postaccelerated to 10 keV kinetic energy just before hitting the detector.

Furthermore, the observations of microfabric and microtextural features of the inspected areas for each sample were accomplished by the ToF–SIMS ability to collect ion-induced secondary electron images. This was the first step that was taken during the ToF–SIMS analytic process that searched for differences in fabric and textural properties along the polished surface of the sample. As a result, different target areas showing characteristic fabrics and textures were selected in sample 010,109–1 (Figs. 2, 3, 4, 5, 6, and 7; Figs. S2 to S3). The characterization of the fabric and texture through the imaging capabilities of ToF–SIMS was essential to recognize the association among fabric/texture, mineralized microstructures, and distribution of preserved biological compounds.

Mass spectral acquisition and image analysis, including the overload of red, green and blue (RGB) images for molecular mapping, were performed with ION-TOF Surface Lab software (version 6.8). The analysis of the molecular fragmentation pattern was completed through the Chemical online tool^109^ and the open source mass spectrometry tool Mmass^110^. Chemspider, METLIN and LIPID MAPS Structure databases^111–113^ were used as sources of information to identify molecular fragments and compounds.

## Supplementary Information

Supplementary Information.
