# Publisher Correction: Unveiling microbial preservation under hyperacidic and oxidizing conditions in the Oligocene Rio Tinto deposit

**DOI:** 10.1038/s41598-021-03255-2

**Published:** 2021-12-21

**Authors:** David C. Fernández‑Remolar, Daniel Carrizo, Mourad Harir, Ting Huang, Ricardo Amils, Philippe Schmitt‑Kopplin, Laura Sánchez‑García, David Gomez‑Ortiz, Per Malmberg

**Affiliations:** 1 CEA, CNRS, IBS, Metalloproteins Unit, Université Grenoble Alpes, 38000 Grenoble, France; 2 Centro de Astrobiología (INTA-CSIC), Madrid, Spain; 3 Research Unit Analytical Biogeochemistry, Helmholtz Zentrum München, Neuherberg, Germany; 4 State Key Laboratory of Lunar and Planetary Sciences, Macau University of Science and Technology, Macau, China; 5 Centro de Biología Molecular Severo Ochoa, Universidad Autónoma de Madrid, Madrid, Spain; 6 Chair of Analytical Food Chemistry, Technical University Munich, 85354 Freising‑Weihenstephan, Germany; 7 ESCET‑Área de Geología, Universidad Rey Juan Carlos, 28933 Móstoles, Madrid Spain; 8 Chemistry and Chemical Engineering, Chalmers University of Technology, Kemivägen 10, 412 96 Gothenburg, Sweden; 9 Present Address: State Key Laboratory of Lunar and Planetary Sciences, Macau University of Science and Technology, Macau, 999078 PR China; 10 Present Address: CNSA Macau Center for Space Exploration and Science, Macau, 999078 PR China

Correction to: *Scientific Reports* 10.1038/s41598-021-00730-8, published online 02 November 2021

The original version of this Article contained errors.

Firstly, citation numbers after reference 11 (with the exception of citations to References 7 and 10) were inaccurate by two.

Additionally, in the Introduction,

“Although different authors report neutral to alkaline conditions for underground Mars^7,8^, the upper part of the planet interior has certainly been affected by the circulation and downwelling of low-pH and oxidizing solutions^8–10^.”

now reads:

“Although different authors report neutral to alkaline conditions for underground Mars^7,8^, the upper part of the planet interior has certainly been affected by the circulation and downwelling of low-pH and oxidizing solutions^9–11^.”

Furthermore, in the Materials and methods section, the subheading ‘Sample 010,109–1’ now reads ‘Sample 010109-1’, and

“Thin sections of sample 010,109–1 were prepared for mineral and textural analysis under an optical microscope and a Scanning Electron Microscope (SEM) coupled with an Electron Dispersive Spectroscopy (EDS) probe, and molecular surface analysis by ToF-SIMS^105^.”

now reads:

“Thin sections of sample 010109-1 were prepared for mineral and textural analysis under an optical microscope and a Scanning Electron Microscope (SEM) coupled with an Electron Dispersive Spectroscopy (EDS) probe, and molecular surface analysis by ToF-SIMS^107^.”

Finally, in the Materials and methods section, under the subheading ‘ToF–SIMS analysis’, exponents were incorrectly captured as citations, and

“To prevent this problem, the thin sections of samples 010,109–1 and BH8-24c were cleaned by surface sputtering with a 100 nA 500 eV oxygen ion beam for 3 s.”

now reads:

“To prevent this problem, the thin sections of sample 010109–1 were cleaned by surface sputtering with a 100 nA 500 eV oxygen ion beam for 3 s.”

The original Article has been corrected.

