# Supplementary material for: Unveiling microbial preservation under hyperacidic and oxidizing conditions in the Oligocene Rio Tinto deposit
Source: Sci Rep. 2021 Nov 2;11:21543. doi: 10.1038/s41598-021-00730-8 (PMC8563943; doi:10.1038/s41598-021-00730-8)
Supplement: Supplementary file 1 — Supplementary Information. [file 41598_2021_730_MOESM1_ESM.pdf]

Supplementary Materials for

Unveiling microbial preservation under hyperacidic and oxidizing conditions in the Oligocene Rio Tinto deposit

David C Fernández-Remolar[1,a,b*], Daniel Carrizo[2], Mourad Harir[3], Ting Huang[4], Ricardo Amils[5], Philippe Schmitt-Kopplin[3,6], Laura Sánchez-García[2], David Gomez-Ortiz[7], Per Malmberg[8]

[1]Univ Grenoble Alpes, CEA, CNRS, IBS, Metalloproteins Unit, F-38000 Grenoble, France. [2]Centro de Astrobiología (INTA-CSIC), Madrid, Spain. [3]Research Unit Analytical Biogeochemistry, Department of Environmental Sciences, Helmholtz Zentrum München, Neuherberg, Germany. [4]State Key Laboratory of Lunar and Planetary Sciences, Macau University of Science and Technology, Macau, China. [5]Centro de Biología Molecular Severo Ochoa, Universidad Autónoma de Madrid, Madrid, Spain. [6]Chair of Analytical Food Chemistry, Technical University Munich, D-85354 Freising-Weihenstephan, Germany. [7]ESCET-Área de Geología, Universidad Rey Juan Carlos, 28933 Móstoles, Madrid, Spain. [8]Chemistry and Chemical Engineering, Chalmers University of Technology, Kemivägen 10, SE-412 96 Gothenburg, Sweden.

Present address: [a]State Key Laboratory of Lunar and Planetary Sciences, Macau University of Science and Technology, Macau 999078, PR China. [b]CNSA Macau Center for Space Exploration and Science, Macau 999078, PR China.

Corresponding author. Tel.: + 853 88972065
[*]E-mail address: dcfremolar@must.edu.mo

This PDF includes:

Detailed version of section "ToF-SIMS spectral analysis: compound classification"

Captions for Figs. S1 to S14

Captions for Tables S1 to S9

Figs. S1 to S14

Tables S1 to S9

ToF-SIMS spectral analysis: compound classification.

The spectral analysis of the sample 010109-1 TAs has provided the identification of different biomolecular fragments and organic compounds. Although the spectra fragmentation patterns are very intricate resulting from secondary ionization between the C-bearing compounds and the Fe- and S-bearing mineral matrix, it has been possible to identify diverse groups of organic compounds and which have been characterized in the upcoming sections 1 to 9.

1. *n*-Alkanes and other straight hydrocarbons with low unsaturation degree

The occurrence of saturated and monounsaturated straight chain hydrocarbons (i.e. *n*-alkanes and alkenes) is supported by the detection of lower mass peaks (< 120 Da) in two different sets. The first set shows strong peaks (I ~ 300 – 70,000 cps) at 43.05, 57.07, 71.09, 85.10, and 99.12 that fits well to positive ions like $C_3H_7^+$, $C_4H_9^+$, $C_5H_{11}^+$, $C_6H_{13}^+$, and $C_7H_{15}^+$ which correspond to a fragmentation of n-alkanes. The second set is characterized by large peaks (I ~ 200 – 100,000 cps) at *m/z* 27.02, 41.04, 55.05, 69.07, 83.09, 97.10 and 111.12 that match n-alkene positive fragments like $C_2H_3^+$, $C_3H_5^+$, $C_4H_7^+$, $C_5H_9^+$, $C_6H_9^+$, $C_7H_{11}^+$, and $C_8H_{15}^+$.The peak intensity and distribution of the two sets of ions suggest that, while *n*-alkanes are common, alkenes are the dominant forms among the straight hydrocarbons. Such distribution supports the presence of a large abundance of large lipid compounds in the Upper Gossan Unit[58], in which straight hydrocarbon backbones should be composed of saturated and monounsaturated chains. The distribution of hydrocarbon fragment like $C_3H_7^+$ and $C_4H_9^+$ follow the glycerolipid and fatty acid one (Fig. S8), suggesting that they are the main sources of biomass in gossan.

The TAs cation fragmentation pattern also occurs as straight hydrocarbons that have formed NH_4^+-bearing adducts[59]. They fit different *m/z* adducts resulting from the combination of the ammonium cation NH_4^+ with aliphatic monounsaturated hydrocarbons like $C_{13:1}$, $C_{14:1}$, $C_{16:1}$, $C_{18:1}$, $C_{19:1}$, $C_{20:1}$, $C_{21:1}$, $C_{22:1}$, $C_{23:1}$, $C_{25:1}$, $C_{26:1}$, $C_{27:1}$, $C_{28:1}$, $C_{34:1}$, $C_{36:1}$, $C_{37:1}$, and $C_{38:1}$ (e.g., peaks at *m/z* 284.33, 494.57, 522.59, and 550.63) (Fig. S6). The total intensity from the adducts ≥ $C_{34:1}$ monounsaturated hydrocarbon is particularly high in TA1 and defines well the filamentous-like pattern characterizing the Group 1 (Figs. 4, and 5), while it is much lower in TA2 and TA3 (Fig. S6). The fragment composition and distribution strongly suggest that the N-bearing compounds and adducts are produced by the ionization of a matrix enriched in different sphingolipids, which are associated with filamentous fungal structures[45,63,64] (Fig. 8).

2. Branched hydrocarbons and isoprenoids

The occurrence of high-intensity peaks at 43.06 and 57.07 is also consistent with the preservation of different branched hydrocarbons, including isoprenoids, that have been detected using GC-MS (Table S1). In fact, the relatively high-intensity peaks in TA1 and TA2 at 225.23 and 239.25 could correspond to positive fragments $C_{16}H_{33}^+$ and $C_{17}H_{35}^+$ that result from the fragmentation of isoprene and/or phytane through the release of the isopropyl and isobutyl fragments.

3. Polycyclic aromatic hydrocarbons (PAHs)

A set of TA1 and TA3 peaks suggests the occurrence of PAHs. They correspond to *m/z* peaks found at 115.05, 128.06, 141.06, 152.05, 165.06, 178.06, 215.07, 276.08, and 289.10. Such series of peaks fit well to cations as $C_9H_7^+$, $C_{10}H_8^+$, $C_{11}H_9^+$, $C_{12}H_8^+$, $C_{13}H_9^+$, $C_{14}H_{10}^+$, $C_{17}H_{11}^+$, $C_{22}H_{12}^+$, and $C_{23}H_{13}^+$, respectively[65,66] (Table S3). The number of TA2 peaks assigned to PAHs is lower than in TA1 and TA3 as only *m/z* masses at 115.05 ($C_9H_7^+$), 128.06 ($C_{10}H_8^+$), 141.06 ($C_{11}H_9^+$), 165.07 ($C_{13}H_9^+$), and 239.08 ($C_{19}H_{11}^+$) have been found to match the PAHs major fragments[66]. The identified PAHs can be assigned to some compounds like indene (C_9H_8), naphthalene ($C_{10}H_8$), acenaphthylene ($C_{12}H_8$), fluorene ($C_{13}H_{10}$), phenanthrene/anthracene ($C_{14}H_{10}$), and methylpyrene ($C_{17}H_{12}$).

The PAHs cation distribution occurs homogeneously in the mineral matrix (Fig. 7). It suggests that PAHs were produced during the organic mineralization in the Upper Gossan unit. Such a process resulted from the combination of the fungal and microbial degradation of cellulose, lignin, pectin, and other polysaccharides[67,68], as well as other sources like woodland fires[69]. This is clearly observed in TA3 where the biological structure is strongly depleted in PAHs, while the matrix shows a high concentration in these compounds (Fig. 7). Such distribution suggests the preservation of biological structures by the mineralization of a ferruginous matrix precipitated from a fluid that transports recalcitrant organic compounds like PAHs and terpenoids.

4. Fatty acids

The mass spectra of negative fragments through ToF-SIMS record a diverse set of fatty acids (FA) ranging from C_{11} to C_{30} (Fig. 9). It includes the occurrence of saturated, unsaturated, and hydroxylated compounds (Table S4), the distribution of which follow the TA fabric (Figs. 4, 6, and 7; Figs. S2 to S4). Assuming that the fatty acid fraction is dominated by aliphatic chains (Table S1), the distribution of the $[M – H]^-$ anions shows that the hexadecanoic acid ($C_{16:0}$) has

the highest intensity peak followed by tetradecanoic ($C_{14:0}$) and pentadecanoic ($C_{15:0}$) acids, respectively. A rough estimation of the FAs abundance through the total amount of the $[M - H]^-$ anion peak intensity shows a higher concentration of fatty acids in TA2 than in TA1, which agrees with a higher concentration in glycerolipids[70] (Fig. 6). The intensity distribution of the saturated and monounsaturated $[M - H]^-$ anions shows that the concentration of FA chains with less than 22 carbon atoms is higher than > *n*-$C_{21:0}$ FAs (Fig. 9). The total sum of the $[M - H]^-$ anion intensity shows that in TA1 and TA2 the saturated and monounsaturated ≤ *n*-C_{20} FAs have a higher intensity than the > *n*-C_{20} FAs, while the total intensity of even exceeds odd FAs in ≤ *n*-C_{20} chains (Fig. 9). Such distribution pattern is also observed in the spectral data obtained from mineralized fungi that is being used as a reference for FA composition[71]. The mineralized fungal reference (Fig. 9) shows the occurrence of ≤ *n*-C_{18} saturated and monounsaturated chains, while the saturated and monounsaturated FAs longer than 18 carbon atoms has a negligible concentration. In the ferruginized fungi[71] the ≤ *n*-C_{20} even fraction exceeds around three times the total intensity of ≤ *n*-C_{20} odd FAs as it is also observed in TA1 and TA2 as well (Fig. 9).

The ToF-SIMS spectral data also show the occurrence of four peaks at *m/z* 301.21, 302.21, 303.22, and 304.22 matching $C_{20}H_{29}O_2^-$, $C_{20}H_{30}O_2^-$, $C_{20}H_{31}O_2^-$, and $C_{20}H_{32}O_2^-$ (Fig. 9), respectively. They can be associated to the $[M - H]^-$ and M^- couplets of polyunsaturated fatty acids (PUFAs) as $C_{20:5}$ and $C_{20:4}$ corresponding to eicosapentaenoic and arachidonic acids. The calculation of the fatty acid abundance using the $[M - H]^-$ anion peak intensity at both $C_{20}H_{29}O_2^-$ and $C_{20}H_{31}O_2^-$ ions shows that the TA1 is ten times more enriched in $C_{20:5}$ and $C_{20:4}$ FAs than TA2. This is the opposite situation found in the saturated and monounsaturated acid chains, which show a higher concentration in TA2. Both eicosapentaenoic and arachidonic PUFAs are also recognized in the mineralized fungi sample[71] that has been used as a reference through the *m/z* peaks at 301.22, 302.22, 303.23, and 304.23 (Fig. 9; Table S4). Some potential hydroxy acids have also been recognized in TA1 as $[M - H]^-$ and $[M]^-$ ions at 383.36, 384.35, 397.36, 411.37, 412.38 (Fig. 9; Table S4). Such fragments fit well $C_{24}H_{47}O_3^-$, $C_{24}H_{48}O_3^-$, $C_{25}H_{49}O_3^-$, $C_{26}H_{51}O_3^-$, and $C_{26}H_{52}O_3^-$, respectively, which can correspond with hydroxy acids > C_{22} like tetracosanoic, pentacosanoic, and hexacosanoic. In TA2, the same three C_{24}, C_{25} and C_{26} hydroxy acids might occur though at much lower concentration as the molecular peaks are hardly recognized, while they have not been found in the spectral data obtained from the ToF-SIMS analysis of the ferruginized fungi[71].

The TA1 and TA2 fatty acid distribution suggests that the record of organic compounds in the Upper Gossan that has resulted intermixing from a heterogeneous biological source including microbial communities and higher plants. The straight-chain fatty acids with less than 22 carbon atoms and the even-over-odd predominance in fatty acids should evidence that the organic compounds in the Upper Gossan comes from the higher plants recycled by fungal and bacterial decomposers (e.g. short chained even fatty acids) and the microbial production of

chemotrophic bacteria (e.g. short-chained odd fatty acids)[72,73]. However, both even and odd fatty acids found in the sample 010109-1 are associated to fungal and non-fungal microbial structures (see Figs. 4-7), which suggests that in the Rio Tinto subsurface fatty acid are not mainly sourced from higher plants though can be a secondary source. In this regard, the occurrence of the $C_{20:5}$ and $C_{20:4}$ PUFAs has been reported in different fungi[72,74], as it has been found in different higher plants. This is the same case for the long chain hydroxylated FAs (e.g., C_{24}, C_{25} and C_{26}) that are considered molecular markers of vascular plants derived from suberin[75,76]. Alternatively, they are also found in fungi[77] that are produced through the enzymatic oxidation of alcohols (e.g. lipoxygenase) and other fatty acids[78]. The FA intensity of TA2 is up to 1.5 order of magnitude higher than TA1 (Fig. 9). As will be discussed below, TA1 shows a high concentration of sphingolipids, while glycerophopholipids are the predominant lipid structures in TA2. In this regard, the higher intensity of FAs strongly correlated with the distribution of glycerophospholipid found in TA2 (Fig. 6), while they also show certain correlation with the distribution of TA1 sphingolipids (Fig. 4).

5. **Glycerolipids and glycerophospholipids**

A diverse set of odd cations occurring in a wide range of masses > 300 Da agrees the fragmentation pattern of different glycerolipids including monoacylglicerids (MAG), diacylglicerids (DAG), and glycerophospholipids (GPL). TA1 to TA3 peaks found at *m/z* 311.28, 313.29, and 339.27 fit positive ions $C_{19}H_{35}O_3^+$, $C_{19}H_{37}O_3^+$, and $C_{21}H_{39}O_3^+$, respectively. They are associated to MAG (Fig. S8) cations like $[M + H – H_2O]^+$, which may take part in the structure of much larger diacyl and triacyl lipidic structures[60]. In TA2, some additional peaks like 353.30, 355.32, and 367.32 corresponding to $C_{22}H_{41}O_3^+$, $C_{22}H_{43}O_3^+$, and $C_{23}H_{43}O_3^+$ could also correspond to $[M + H – H_2O]^+$ MAG fragments (Fig. S9).

The TA2 and TA3 spectral data show a set of > *m/z* 400 peaks that follows a fragmentation pattern of larger acylglycerids (Fig. 8, Fig. S10a; Table S5). Such a pattern is defined by multiple odd peaks[70] found at 467.42, 479.41, 481.43, 493.43, 495.45, 507.45, 509.46, 521.47, 523.48, 533.47, 535 49, 537.47, 547.48, 549.49, 551.51, 563.52, 565.53, 575.51, 577.52, 579.54, 593.57, 605.56, 607.58, 619.59, 621.60, 633.59, 635.59, 647.63, 649.61, 661.62, 663.67, 675.64, 677.67, 689.68, 691.68, 703.69, and 705.71. They fit well the fragments like $C_{29}H_{55}O_4^+$, $C_{30}H_{55}O_4^+$, $C_{30}H_{57}O_4^+$, $C_{31}H_{57}O_4^+$, $C_{31}H_{59}O_4^+$, $C_{32}H_{59}O_4^+$, $C_{32}H_{61}O_4^+$, $C_{33}H_{61}O_4^+$, $C_{33}H_{63}O_4^+$, $C_{34}H_{61}O_4^+$, $C_{34}H_{63}O_4^+$, $C_{34}H_{65}O_4^+$, $C_{35}H_{63}O_4^+$, $C_{35}H_{65}O_4^+$, $C_{35}H_{67}O_4^+$, $C_{36}H_{67}O_4^+$, $C_{36}H_{69}O_4^+$, $C_{37}H_{67}O_4^+$, $C_{37}H_{69}O_4^+$, $C_{37}H_{71}O_4^+$, $C_{38}H_{73}O_4^+$, $C_{39}H_{73}O_4^+$, $C_{39}H_{75}O_4^+$, $C_{40}H_{75}O_4^+$, $C_{40}H_{77}O_4^+$, $C_{41}H_{77}O_4^+$, $C_{41}H_{79}O_4^+$, $C_{42}H_{79}O_4^+$, $C_{42}H_{81}O_4^+$, $C_{43}H_{81}O_4^+$, $C_{43}H_{83}O_4^+$, $C_{44}H_{83}O_4^+$, $C_{44}H_{85}O_4^+$, $C_{45}H_{85}O_4^+$, $C_{45}H_{87}O_4^+$, $C_{46}H_{87}O_4^+$, and $C_{46}H_{89}O_4^+$, respectively (Table S5; Fig. S10b). The cation composition agrees with the occurrence of diglyceride fragments that are primarily built by different chains of saturated FAs, and

monounsaturated chains as secondary components. They also occur as minor components in target area A1 at *m/z* 533.55, 535.57, 537.56, 547.56, 577.58, 593.55, 605.49, 619.53, 635.50 corresponding to some fragments listed above as $C_{34}H_{61}O_4^+$, $C_{34}H_{63}O_4^+$, $C_{34}H_{65}O_4^+$, $C_{35}H_{63}O_4^+$, $C_{37}H_{69}O_4^+$, $C_{38}H_{73}O_4^+$, $C_{39}H_{73}O_4^+$, $C_{40}H_{75}O_4^+$, $C_{41}H_{79}O_4^+$, respectively. The detection of diglyceride structures is additionally supported by the occurrence of different carbonyl bearing fragments[79] at 211.22, 239.25, 253.25, 265.26, 267.28 and, 309.31 observed in TA1 to TA3, which match $C_{14}H_{27}O^+$, $C_{16}H_{31}O^+$, $C_{17}H_{33}O^+$, $C_{18}H_{33}O^+$, $C_{18}H_{35}O^+$, and $C_{21}H_{41}O^+$ (Table S5).

Additionally, different peaks at *m/z* 30.03, 58.07, 59.07, 72.08, 86.10, 102.09, 104.11, 125.00, 166.06, 184.07 found in the three areas TA1 to TA3, match cations as CH_4N^+, $C_3H_8N^+$, $C_3H_9N^+$, $C_4H_{10}N^+$, $C_5H_{12}N^+$, $C_5H_{12}NO^+$, $C_5H_{14}NO^+$, $C_2H_6PO_4^+$, $C_5H_{13}NPO_3^+$, and $C_5H_{15}NPO_4^+$, which are well known as fragments of head groups for choline and phosphatidylcholine[79]. The co-occurrence of the three groups of fragments that originate from diacylglycerides, carbonyls and phosphatidylcholine strongly support the preservation of different glycerophosphocholines (Fig. 8) whose concentration is higher in TA2 and TA3 (Fig. S11), while in TA1 they appear as minor components. This is supported by the occurrence of a diverse set of fatty acids that could have partly sourced from the fragmentation of glycerophosphocholines. The presence of glycerophospholipids is supported by different peaks found at *m/z* 700.53, 712.46, 726.51, 730.56, and 749.46 (Fig. S11) which occur at higher intensity in TA2. Such a set of peaks meet anions as $C_{39}H_{75}NO_7P^-$, $C_{38}H_{67}NO_9P^-$, $C_{40}H_{73}NO_8P^-$, $C_{40}H_{77}NO_8P^-$, and $C_{38}H_{70}O_{12}P^-$ suggesting that the glycerophospholipids are diverse in the Upper Gossan unit[79].

6. Sphingolipids (ceramides)

The presence of different even peaks in TA1, TA2 and TA3 (Figs. 4 to 7) at *m/z* 264.27, 266.29, 282.29, 284.32, 298.28, 304.30, 308.31, and 310.33 can be assigned to fragmented sphingolipid backbone chains[80] like $C_{18}H_{34}N^+$, $C_{18}H_{36}N^+$, $C_{18}H_{36}NO^+$, $C_{18}H_{38}NO^+$, $C_{18}H_{36}NO_2^+$, $C_{21}H_{38}N^+$, $C_{20}H_{38}NO^+$, and $C_{20}H_{40}NO^+$, respectively. They can be produced through two main dehydration pathways[77] like $[M + H - H_2O]^+$ and $[M + H - 2H_2O]^+$ on sphingenine ($C_{18}H_{37}NO_2$), sphinganine ($C_{18}H_{39}NO_2$), amino-methyl-nonadece-triol ($C_{20}H_{41}NO_3$), and phytosphingosine ($C_{20}H_{43}NO_3$) (Table S6). The peaks at *m/z* 282.31 and 284.33 ($C_{18}H_{36}NO^+$ and $C_{20}H_{40}NO^+$) show a particularly high intensity above 400 cps suggesting that sphingenine and sphinganine should be the most abundant sphingolipid backbones in the sample. The relatively high peak at 310.33 corresponding to $C_{20}H_{40}NO^+$ high intensity (> 80 cps) showing that the phytosphingosine is a secondary component in the sphingolipid inventory of the Upper Gossan unit. In addition, a TA3 peak at *m/z* 368.35 (I ~ 130 cps) fits well to the $C_{24}H_{50}NO^+$fragment suggesting that in the Upper Gossan deposits there are likely larger sphingenina-type compounds[81].

TA1 shows dozens of high even peaks (> 500 Da) that fit well the fractionation of different ceramides. However, in TA2 and TA3 such a peak series occur either showing a lower intensity or disappear. Ceramide cation peaks have been found at *m/z* 534.57 ($C_{36}H_{72}NO^+$), 562.57 ($C_{37}H_{72}NO_2^+$), 564.61 ($C_{37}H_{74}NO_2^+$), 566.62 ($C_{37}H_{76}NO_2^+$), 590.59 ($C_{39}H_{76}NO_2^+$), 604.64 ($C_{40}H_{78}NO_2^+$), 612.58 ($C_{38}H_{78}NO_4^+$), 618.63 ($C_{41}H_{80}NO_2^+$), 632.63 ($C_{42}H_{82}NO_2^+$), 638.60 ($C_{40}H_{80}NO_4^+$), 640.58 ($C_{39}H_{78}NO_5^+$), 654.61 ($C_{40}H_{80}NO_5^+$), 666.62 ($C_{41}H_{80}NO_5^+$), 668.61 ($C_{41}H_{82}NO_5^+$), 682.64 ($C_{42}H_{84}NO_5^+$), 696.65 ($C_{43}H_{86}NO_5^+$), 710.63 ($C_{43}H_{84}NO_6^+$), and 714.64 ($C_{43}H_{88}NO_6^+$).

In TA2 and TA3, a similar set of ions (Table S6) are found at *m/z* 534.58, 562.58, 564.61, 590.60, 596.57,604,62, 612.52, 618.58, 624.57,632.61, 638.59, 640.58, 652.62, 654.59, 666.62, 668.62, 682.63, 696.67, 710.64, and 714.65. They correspond to the even positive ions observed in TA1 like $C_{36}H_{72}NO^+$, $C_{37}H_{72}NO_2^+$, $C_{37}H_{74}NO_2^+$, $C_{39}H_{76}NO_2^+$, $C_{40}H_{70}NO_2^+$, $C_{40}H_{78}NO_2^+$, $C_{38}H_{78}NO_4^+$, $C_{41}H_{80}NO_2^+$, $C_{39}H_{78}NO_4^+$, $C_{42}H_{82}NO_2^+$, $C_{42}H_{82}NO_2^+$, $C_{40}H_{80}NO_4^+$, $C_{39}H_{78}NO_5^+$ (640.5837), $C_{41}H_{82}NO_4^+$ (652.62), $C_{40}H_{80}NO_5^+$ (654.60), $C_{41}H_{80}NO_5^+$ (666.61), $C_{41}H_{80}NO_5^+$ (668.63), $C_{42}H_{84}NO_5^+$ (682.63), $C_{43}H_{84}NO_6^+$ (710.64) and $C_{43}H_{88}NO_6^+$ (714.65). The lower peak intensity of the spectral data of TA2 and TA3 suggests that ceramides are less abundant than in TA1.

The detection of diverse N-bearing cations with 1 to 6 oxygen atoms suggests the presence of different types of sphingolipids with a length chain higher than C_{38}. This is indirectly supported by the occurrence of sphinganine-type backbones (e.g., $C_{18}H_{36}NO^+$ $C_{20}H_{40}NO^+$, and $C_{24}H_{50}NO^+$) that bear half of the carbon atoms in the ceramide structure. The mass distribution of positive ions above 530 Da agrees with the preservation of diverse ceramides including cerebrosides, and sphingomyelins[80]. Although the identification of different ceramide fragments with the same length chain but number of oxygens varying between 1 and 6 is consistent with different dehydration pathways in the ionization process[80], it could also originate from the loss of ceramide hydroxyls during the early mineralization. Furthermore, the decrease in oxygen and carbons numbers could also result from fragmentation of the glycosidic head in cerebrosides during the ionization process[80,82] (Fig. S12). However, microbial degradation cannot be excluded as a cause for the loss of the glycosidic groups.

The ceramide *m/z* cations appear together with three sharp peaks (intensity > 230 cps) found in the three TA1 to TA3 (Figs. 4 to 7), which are found at 494.56, 522.59, and 550.62. They could be NH_4^+-bearing positive adducts like $C_{34}H_{72}N^+$ ($NH_4^+ + C_{34:1}$), $C_{36}H_{76}N^+$ ($NH_4^+ + C_{36:1}$) and $C_{38}H_{80}N^+$ ($NH_4^+ + C_{38:1}$) that correspond to the hydrocarbon fragment of the main chains of three different hydrocarbons associated to ceramides. The abundance of monounsaturated aliphatic adducts suggests that the ionization process induces the formation of secondary alkenes which combines with NH_4^+ before the adduct formation.

7. Peptide fragments and amino acids

TA1, TA2 and TA3 have provide positive and negative peaks (< 150 Da) that match well a diverse series of N-bearing fragments. They are also associated to secondary and other simpler fragments that lack nitrogen, but which come together in the same structures. The distribution of N-bearing and non-N-bearing cationic fragments outline the microstructures defined as Groups 3 and 7 (Figs. 4 to 7). They correspond to peaks (Table S8) at *m/z* 28.03 (CH_2N^+), 42.04 ($C_2H_4N^+$), 43.06 ($C_3H_7^+$), 44.05 ($C_2H_6N^+$), 56.06 ($C_3H_6N^+$), 57.04 ($CH_3N_3^+$), 84.09 ($C_5H_{10}N^+$), 86.07 ($C_3H_8N_3^+$), 86.10 ($C_5H_{12}N^+$), 110.08 ($C_6H_{10}N_2^+$), 111.09 ($C_5H_9N_3^+$), 112.09 ($C_5H_{10}N_3^+$), 120.09 ($C_8H_{10}N^+$), 122.07 ($C_6H_8N_3^+$), 123.09 ($C_7H_{11}N_2^+$), 124.09 ($C_6H_{10}N_3^+$), 125.11 ($C_7H_{13}N_2^+$), 135.09 ($C_8H_{11}N_2^+$), 136.09 ($C_7H_{10}N_3^+$), 137.11 ($C_7H_{11}N_3^+$), and 138.10 ($C_7H_{12}N_3^+$); as well as peaks at 93.04 ($C_5H_5N_2^-$), 117.03 ($C_4H_5O_4^-$), 211.06 ($C_{13}H_9NO_2^-$), 212.07 ($C_{13}H_{10}NO_2^-$), 279.16 ($C_{18}H_{19}N_2O^-$), and 280.16 ($C_{18}H_{20}N_2O^-$).

Cations like $C_3H_8N^+$, $C_4H_8N^+$, $C_5H_{12}N^+$, and $C_8H_{10}N^+$ have been associated with main fragments of proteins[83], fragments of multiple amino acids (e.g. $C_3H_6N^+$, $C_5H_{10}N^+$, $C_5H_{12}N^+$), and specific fragment for some amino acids including lysine ($C_5H_{10}N^+$), aspargarine ($C_3H_7N_2O^+$), and aspartic acid ($C_4H_5O_4^-$)[84,85]. The potential preservation of protein fragments and amino acids in the oldest deposits of the acidic Rio Tinto basin like the Upper Gossan unit is suggested by the detection of peptidic chains in the 2.1 Ma Rio Tinto Terrace[26].

8. Steroids and hopanoids

Sample 010109-1 shows *m/z* peaks that are characteristic of different cyclic triterpenoids like steroids. However, some of the diagnostic cations have either weak peaks or occur as shoulders siding larger peaks, which prevent the compound characterization at the level that have been done by GC-MS (Table S1). Although the ToF-SIMS spectral analysis shows a similar result, the steroids are identified through to observation of distinctive peaks. It is not the case for hopanoids, since the peaks that could be assigned to such lipids are absent or fit better with other compounds like sphingolipids or glycerolipids. This strongly agrees with the GC-MS results where hopanoids are absent.

The occurrence of steroids in the sample collected atop the Upper Gossan unit is suggested by the presence of different *m/z* sharp peaks that can be attributed to the steroid fragmentation. They occur in TA1 to TA3 at an average of *m/z* 147.11 ($C_{11}H_{15}^+$), 161.14 ($C_{12}H_{17}^+$), 201.17

($C_{15}H_{21}^+$), and 215.18 ($C_{16}H_{23}^+$). Diagnostic fragment masses for steroids[86] have been also found (Table S8) as weak peaks at 149.13 ($C_{11}H_{17}^+$), 191.18 ($C_{14}H_{23}^+$), 203.18 ($C_{15}H_{23}^+$), and 257.22 ($C_{19}H_{29}^+$). In addition, masses assigned to ions like 367.37, 383.34, and 402.37 fitting $C_{27}H_{43}^+$, $C_{27}H_{43}O^+$, and $C_{27}H_{48}NO^+$ suggest the presence of C_{27} steroids (e.g. $C_{27}H_{44}O$) and cholesterol derivatives. In this regard, cholesterol, and likely cholesteryl-sulfate ($C_{27}H_{46}O$ and $C_{27}H_{46}SO_4$) have been identified through its main diagnostic peaks (Table S8) occurring at 369.39 ($C_{27}H_{45}^+$), 386.36 (cholesterol molecular peak $C_{27}H_{46}O^+$); and a series of very intense (I < 500 cps) negative peaks at 465.31 ($C_{27}H_{45}SO_4^-$), 466.30 ($C_{27}H_{46}SO_4^-$), and 467.31 ($C_{27}H_{47}SO_4^-$), which match well with the cholesteryl-sulfate anion. The occurrence of such SO_4-bearing lipid is additionally supported by a set of diverse negative ions (Table S8) bearing both SO_3 and SO_4 groups as $C_8H_7SO_3^-$ (183.01), $C_{12}H_{25}SO_4^-$ (265.15), $C_{14}H_{29}SO_4^-$ (293.17), and $C_{19}H_{31}SO_3^-$ (339.19). The higher peak intensity of cholesteryl-sulfate against cholesterol suggests that the sulfate bearing lipid is more abundant in TA1, while cholesterol is relatively more abundant in TA2.

A set of weak peaks found in TA1 to TA3 at *m/z* 371.37 ($C_{27}H_{46}^+$), and *m/z* 388.37 ($C_{27}H_{48}O^+$) could correspond to the $[M – H_2O]^+$ and M^+ cholestanol or dihydrocholesterol ions (Table S8). This is supported by the observation of minor positive peaks at 406.41, and 444.29 corresponding $[M + NH_4^+]$ and $[M + Fe^+]$ adducts. Furthermore, the presence of weak peaks (I < 50 cps) at 409.35, 410.35, 411.39, 412.38, 412.39 and 430.39 in the TA1 to TA3 areas fit well with a set of ions like $C_{29}H_{45}O^+$, $C_{29}H_{46}O^+$, $C_{29}H_{47}O^+$, and $C_{29}H_{48}O^+$, corresponding to $[M – H]^+$, and $[M]^+$ of stigmasterol ($C_{29}H_{48}O$) and its unsaturated derivatives ($C_{29}H_{46}O$) (Table S8). The occurrence of peaks at *m/z* 395.33, 396.34, and 400.37 that match $C_{28}H_{43}O^+$, $C_{28}H_{44}O^+$, and $C_{28}H_{48}O^+$ cations also suggests the presence of C_{28} steroids like ergosterol and campesterol that have been detected by GC-MS analysis (Table S1).

The detection of different peaks as fragments of different sterols shows that eukaryotic organisms like fungi and plants were the main contributors to gossan´s biolipid inventory. However, the most abundant sterol in terms of intensity like cholesteryl sulfate suggests that the mineralization process driven by enriched sulfate solution produced secondary organic complexes composed of lipid structures bonded to inorganic compounds.

9. **Polyphenols, lignin derivatives and other phytochemicals**

The ion mass distribution of some distinctive peaks defining Groups 5 and 6 (Figs. 4, and 7) suggests the occurrence of compounds derived of phytochemicals like terpenoids and polyphenols. Such ions have been found at cation peaks (Table S9) like *m/z* 76.03, 77.04, 104.03, 105.03, 149.02, 149.06, 150.03, 163.04, 165.06, 166.04, 167.07, 168.04, 169.09, and 179.07 fitting $C_6H_4^+$, $C_6H_5^+$, $C_7H_4O^+$, $C_7H_5O^+$, $C_8H_5O_3^+$, $C_9H_9O_2^+$, $C_8H_6O_3^+$, $C_9H_7O_3^+$, $C_9H_9O_3^+$,

$C_5H_{10}O_6^+$, $C_8H_8O_4^+$, $C_9H_{11}O_3^+$, $C_9H_{13}O_3^+$, and $C_{10}H_{11}O_3^+$. The cation set of Groups 5 and 6 come together with a series of negative ions at 73.03, 77.04, 105.04, 120.02, 121.03, 163.05, 165.03 that match well $C_3H_5O_2^-$, $C_6H_5^-$, $C_7H_5O^-$, $C_7H_4O_2^-$, $C_7H_5O_2^-$, $C_6H_{11}O_5^-$, and $C_8H_5O_4^-$, respectively (Table S9).

The mass distribution of such collection of ions resembles a series of incomplete fragmentation of degraded oligomeric units that are differentiated by the number of oxygen atoms and the methyl groups. This is observed in the series of positive ions $C_7H_4O^+$, $C_7H_5O^+$, $C_8H_5O_3^+$, and $C_8H_6O_3^+$ occurring in G5, which likely come from the loss of methyl groups in fragments like $C_5H_{10}O_6^+$, $C_9H_{11}O_3^+$, and $C_9H_{13}O_3^+$ in G6. The occurrence of such a couple of different set of ions partly match to the fragmentation series of vanillin ($C_9H_{10}O_3$) or cinnamic acid ($C_{10}H_{12}O_3$) derivatives[87-89] that result from the decomposition of lignin by different microbial agents. The complementary occurrence of both sets of positive ions in two different microsites of TA1 (Fig. 5) could correspond with two different degradation stages from the same primary compounds. However, the peak distribution in both sets of positive and negative ions could also be interpreted as the preservation of few phytochemicals terpenoid from aromatic plants like derivatives of heliotropin or piperonal ($C_8H_6O_3$). They are phytochemicals currently metabolized and produced by soil bacteria[56]. In this instance, the distribution of the two sets of different ions could represent variation in the phytochemical composition of a plant residue.

Furthermore, the spectral analysis of sample 010109-1 has provided a set of negative ions of unknown origin occurring at *m/z* 650.07, 666.06, 668,08, 736.15, 737.15, and 738.14. They only occur in the TA1 as Group 8 (Figs. 4 and 10), suggesting the presence of compounds with a high number of O and/or N, which is compatible with the preservation of larger fragments of polysaccharides or polyketides.

Captions of Supplementary Figures

Fig. S1. Profile of the level of unsaturation of the Upper Gossan sample 010109-1 as obtained from the ESI(-)-FTICR-MS analysis. (a) van Krevelen diagram and (b) mass edited H/C ratios of all assigned molecular members (i.e., CHO, CHNO and CHOS) (see also Fig. 3). (c) Mass edited double bound equivalent (DBE) values. Color-code dots represent the assigned molecular compositions corresponding to (gray color) one phenyl ring and more, and (black color) saturated and unsaturated (no phenyl ring) compounds. Circular areas indicate relative mass peak intensity.

Fig. S2. Visible and SEM TA1 images of sample 010109-1 collected in Peña de Hierro. In addition, ToF-SIMS molecular images are cropping the SEM imaging to examine how the composition is following the TA1 microfabric. Interestingly, K^+ and Na^+ fit the distribution of silica suggesting that some clays could occur infilling porosity.

Fig. S3. Visible and SEM TA2 images of sample 010109-1 collected in Peña de Hierro. In addition, ToF-SIMS molecular images are cropping the SEM imaging to examine how the composition is following the TA1 microfabric.

Fig. S4. ToF-SIMS images of the ion inorganic distribution in TA1 and TA2. Anions like PO_2^-, PO_3^-, NO_2^-, and NO_3^- occur as circular to ovoidal tinny mineralizations (< 20 microns) suggesting a microbial origin. SO_2^- and SO_3^- outline filamentous structures and intricated networks of branched structures suggesting a selective mineralization by sulfates.

Fig. S5. ToF-SIMS images of the spatial distribution of different positive fragments including NH_4^+, $Na_3SO_4^+$, $K_2NaSO_4^+$, Na+, and K^+ that trace the same biological structure. The co-occurrence between them strongly suggests the association between certain primary biological composition like N-bearing lipids and the mineralization through different sulfate phases. The high intensity in K and Na suggests that the primary sulfate could have been a jarositic phase.

Fig. S6. Occurrence of N-bearing adducts in TA1 (a), TA2 (b), and TA3 (c) showing a strong affinity with certain biological structures which could correspond to fungi.

Fig. S7. ToF-SIMS image pattern plotting intensity at *m/z* 58.01 (a), and the anion intensity sum at *m/z* 43.02, 45.00, 53.00, 59.02, 71.02, 85.03, 87.01, 99.02, and 101.04 (b) which characterize group G2 in TA1. The white square (a) traces a microstructure pattern which is also shown in G1.

Fig. S8. ToF-SIMS mapping of propyl and butyl cations, which show a strong affinity with the occurrence of major lipidic structures recognized in TA1, TA2, and TA3.

Fig. S9. Different MAG structures found at *m/z* 311.28 (1), 313.29 (2), 339.29 (3), 355.32 (4), and 367.32 (5). MAGs in black were found in TA1 to TA3, while MAGs in blue were only found in TA2. The MAG higher diversity in TA2 agrees with a higher acylglyceride intensity.

Fig. S10. Production of cation $C_{35}H_{67}O_4^+$, which has a high intensity in (a) TA2 and TA3 (< 1800 cps) and 551.53, through the fractionation pathway of a glycerophospholipid. Such a compound could have been identified at *m/z* 730.56 (Supplementary Fig. 10). b) Occurrence of DAG fragments larger than *m/z* 660 to 700 Da; a ceramide fragment (CER) is also found at an average of *m/z* 668.63 Da.

Fig. S11. Occurrence of different anion peaks in TA2 (brown) which masses at *m/z* 700.53, 712.47, 726.51, 730.56, and 749.46 fit different glycerophospholipid ions like $C_{39}H_{75}NO_7P^-$, $C_{38}H_{67}NO_9P^-$, $C_{40}H_{73}NO_8P^-$, $C_{40}H_{77}NO_8P^-$, and $C_{38}H_{70}O_{12}P^-$, respectively. TA1 spectra is shown in blue to emphasize compositional differences with TA2 as a much lower content in acylglycerids and glycerophospholipids.

Fig. S12. Fragmentation pathway of a C_{44} glucosylceramide ($C_{44}H_{87}NO_{10}$) showing the production of the fragment at *m/z* 640.59 Da (see Supplementary Table 6).

Fig. S13. Flow diagram of sample preparation and compound extraction in GC/MS and FT-ICR MS.

Fig. S14. Core recovered in Peña de Hierro showing well preserved shales of the Carboniferous Culm group (Cg) with a high content in organics (a). Such organic compounds (Cg) materials have been strongly oxidized and leached (b) by the acidic solutions of Rio Tinto. The weathered shales are topped by the Pleistocene Intermediate Terrace (IT) of Rio Tinto. c) Show a closer detail of the shales (Cg) that have been transformed to kaolinite and lost its organic content.

Fig. S15. Image and elemental spectra obtained through a SEM-EDS analysis of sample 010109-1 collected in Peña de Hierro. It shows the alternation between goethite (Go) and hematite (He), which are the result of the final maturation of a primary oxysulfate and/or oxyhydroxide phase, where hematite is the endmember in the maturation process (Fig. 2). While goethite shows traces of S (Spectrum 3) that records the primary composition of the gossan mineralization, the hematite does not (see Spectra 1 and 2). Spectrum 4 corresponds with a quartzic element. The sample microstructure showing the typical colomorphic fabric and a composition devoid of different cations (e.g. Mg, K, Na and Ca) strongly support that the biological compounds are primary. Spectra units are in counts per second (cts).

Fig. S16. SEM-EDS analysis of another area in the sample 010109-1 showing a goethite body (Go) that is coated by a hematite lamina (He). The goethite body shows internal filamentous microstructures that are coated by hematite which is the endmember of the mineral maturation in the gossan. They are both covered by an hematite and gossan alternation (He/Go) with botroidal and colomorphic microstructure that are characteristic of a gossan fabric. Fruthermore, no traces of cations suggesting secondary weathering (Ca, Mg, K, and Na) have found but traces of S in the goethite rich areas (Spectra 1 and 3). Spectrum 2 shows the elemental composition of a hematite-rich area, where Fe show a highr intensity. Such features also support the preservation of primary structures and molecular composition with no input of secondary biological compounds resulting from secondary weathering processes. Spectra units are in counts per second (cts).

Captions of Supplementary Tables

Table S1. List of organic compounds identified in the Upper Gossan sample of Peña de Hierro through GC/MS analysis.

Table S2. Characterization of the different morphological groups through the molecular distribution in the Upper Gossan sample of Peña de Hierro that is obtained using the mapping capabilities of the ToF-SIMS.

Table S3. Positive $[M\text{-}H]^+$ and M^+ ions of different polycyclic aromatic hydrocarbons (PAHs) preserved in the Upper Gossan materials of Peña de Hierro.

Table S4. Fatty acid list identified in sample 010109-1 collected in the Upper Gossan deposits of Peña de Hierro using ToF-SIMS throught the main $[M - H]^-$ ion. Letters bolded in red mark the most abundant fatty acids in terms of intensity (cps) (See Fig. 9).

Table S5. Different positive and negative ions of acylglycerides obtained through the ToF-SIMS spectral analysis of the Upper Gossan deposits in Peña de Hierro. The acylglycerid peaks are well defined in the biological filamentous structures of Group 9 found in TA2.

Table S6. Different positive fragments of sphingolipids (upper table) and ceramides (lower table) found in sample 010109-1 through ToF-SIMS spectral analysis.

Table S7. List of positive and negative ions produced from the fragmentation of peptidic chains and/or amino acids that have been obtained by the ToF-SIMS spectral analysis of sample 010109-1 collected in the Upper Gossan of Pena de Hierro.

Table S8. List of fragments attributed to different steroids that have been recognized in the Upper Gossan sample 010109-1 through the ToF-SIMS spectral analysis.

Table S9. List of positive and negative ions from the fragmentation of polyphenolic and phytochemical compounds obtained by the ToF-SIMS spectral analysis of sample 010109-1 collected in the Upper Gossan of Peña de Hierro.

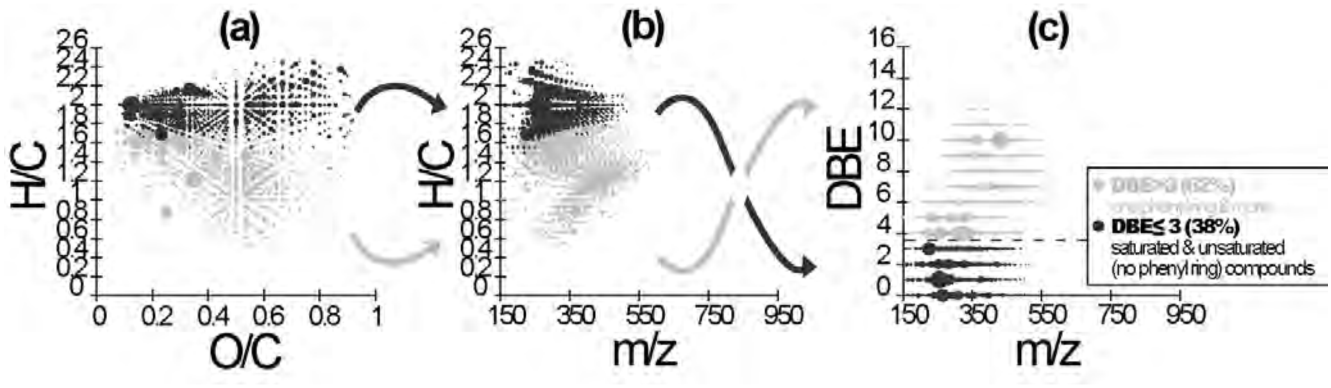
(a)
(b)
(c)
H/C
O/C
m/z
DBE
DBE≤ 3 (38%)
saturated & unsaturated
(no phenyl ring) compounds

Fig. S1

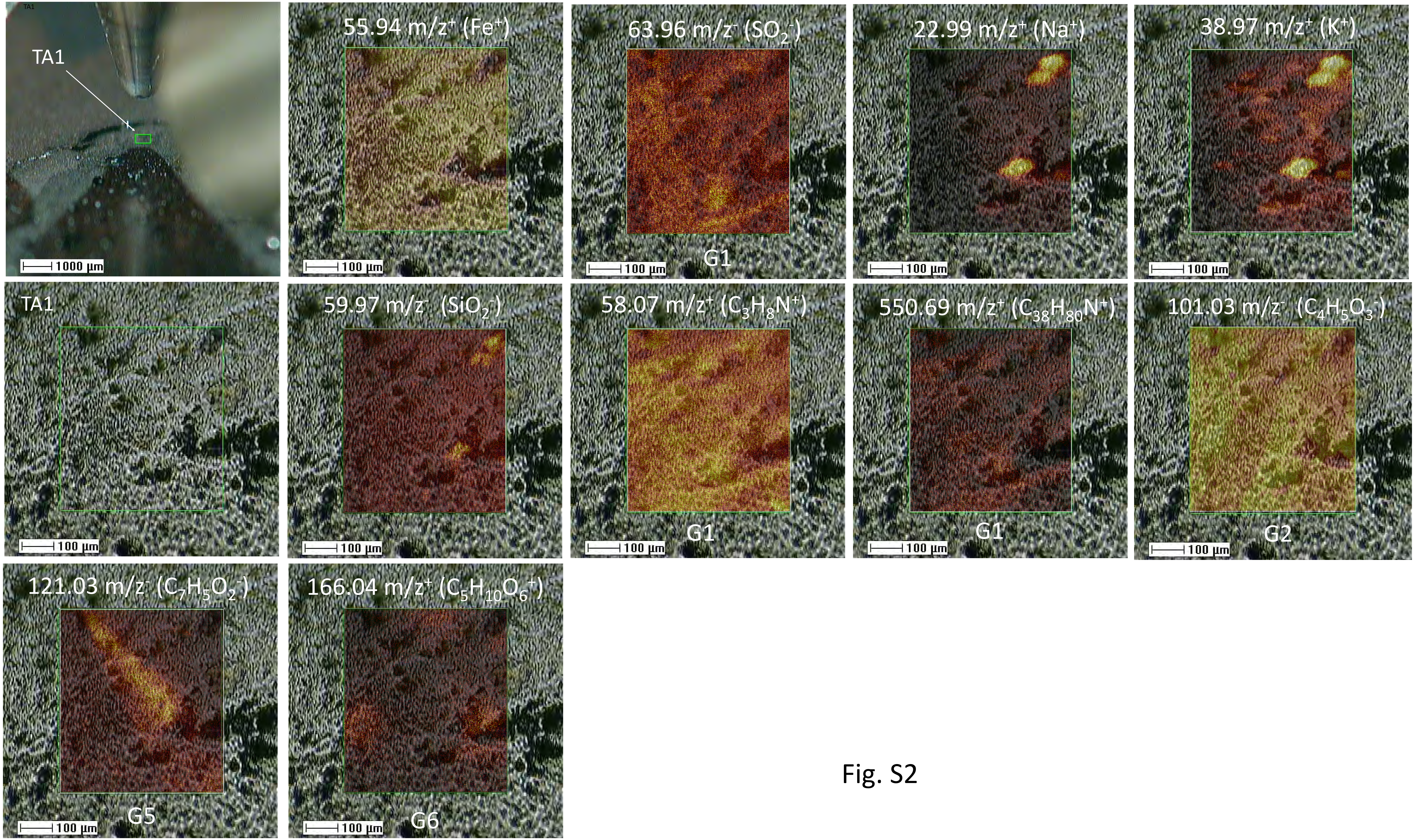
TA1
1000 µm
55.94 m/z+ (Fe+)
100 µm
63.96 m/z- (SO2-)
G1
100 µm
22.99 m/z+ (Na+)
100 µm
38.97 m/z+ (K+)
100 µm
TA1
100 µm
59.97 m/z- (SiO2-)
100 µm
58.07 m/z+ (C3H8N+)
G1
100 µm
550.69 m/z+ (C38H80N+)
G1
100 µm
101.03 m/z- (C4H5O3-)
G2
100 µm
121.03 m/z- (C7H5O2-)
G5
100 µm
166.04 m/z+ (C5H10O6+)
G6
100 µm

Fig. S2

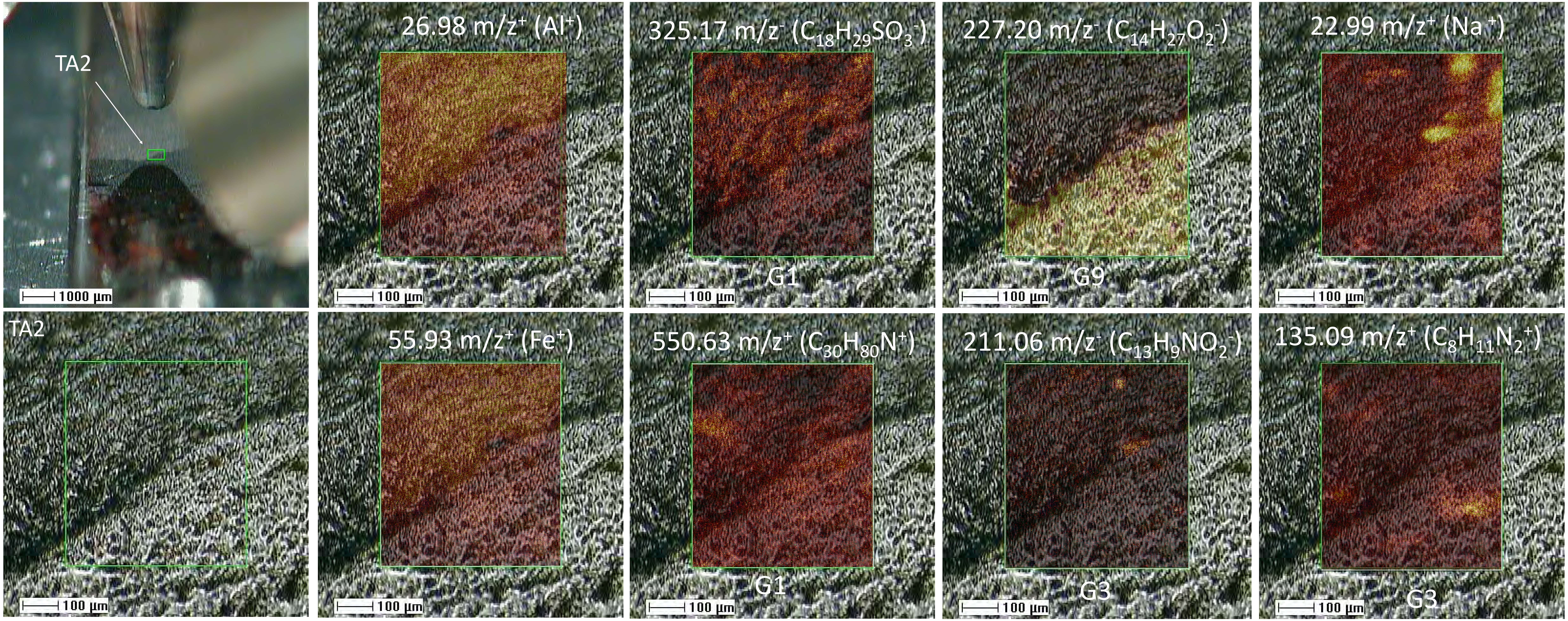
TA2
1000 µm
26.98 m/z+ (Al+)
100 µm
325.17 m/z- (C18H29SO3-)
G1
100 µm
227.20 m/z- (C14H27O2-)
G9
100 µm
22.99 m/z+ (Na+)
100 µm
TA2
100 µm
55.93 m/z+ (Fe+)
100 µm
550.63 m/z+ (C30H80N+)
G1
100 µm
211.06 m/z- (C13H9NO2-)
G3
100 µm
135.09 m/z+ (C8H11N2+)
G3
100 µm

Fig. S3

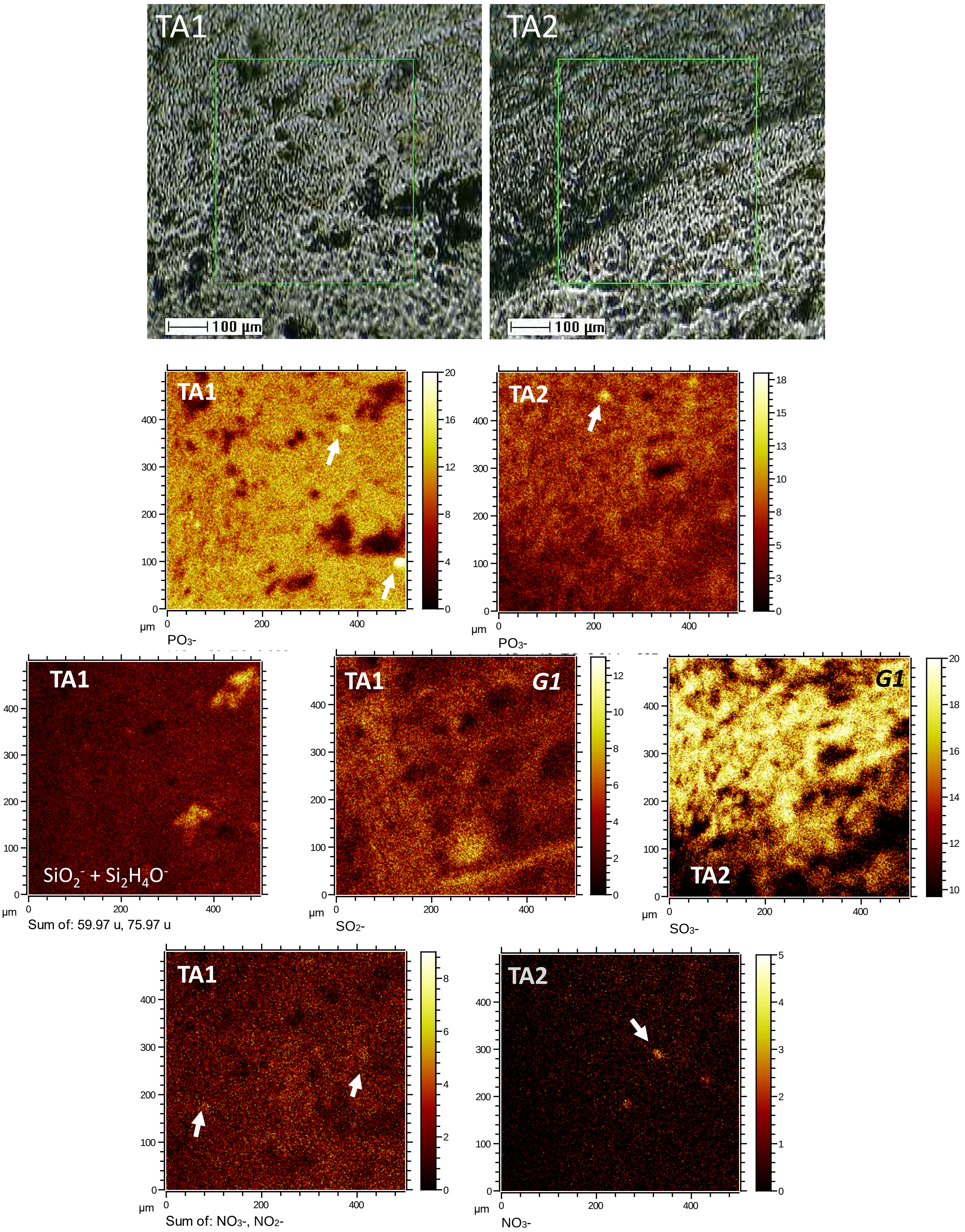
TA1
TA2
100 µm
100 µm
PO3-
PO3-
SiO$_2^-$ + Si$_2$H$_4$O$^-$
Sum of: 59.97 u, 75.97 u
G1
SO2-
SO3-
Sum of: NO3-, NO2-
NO3-

Fig. S4

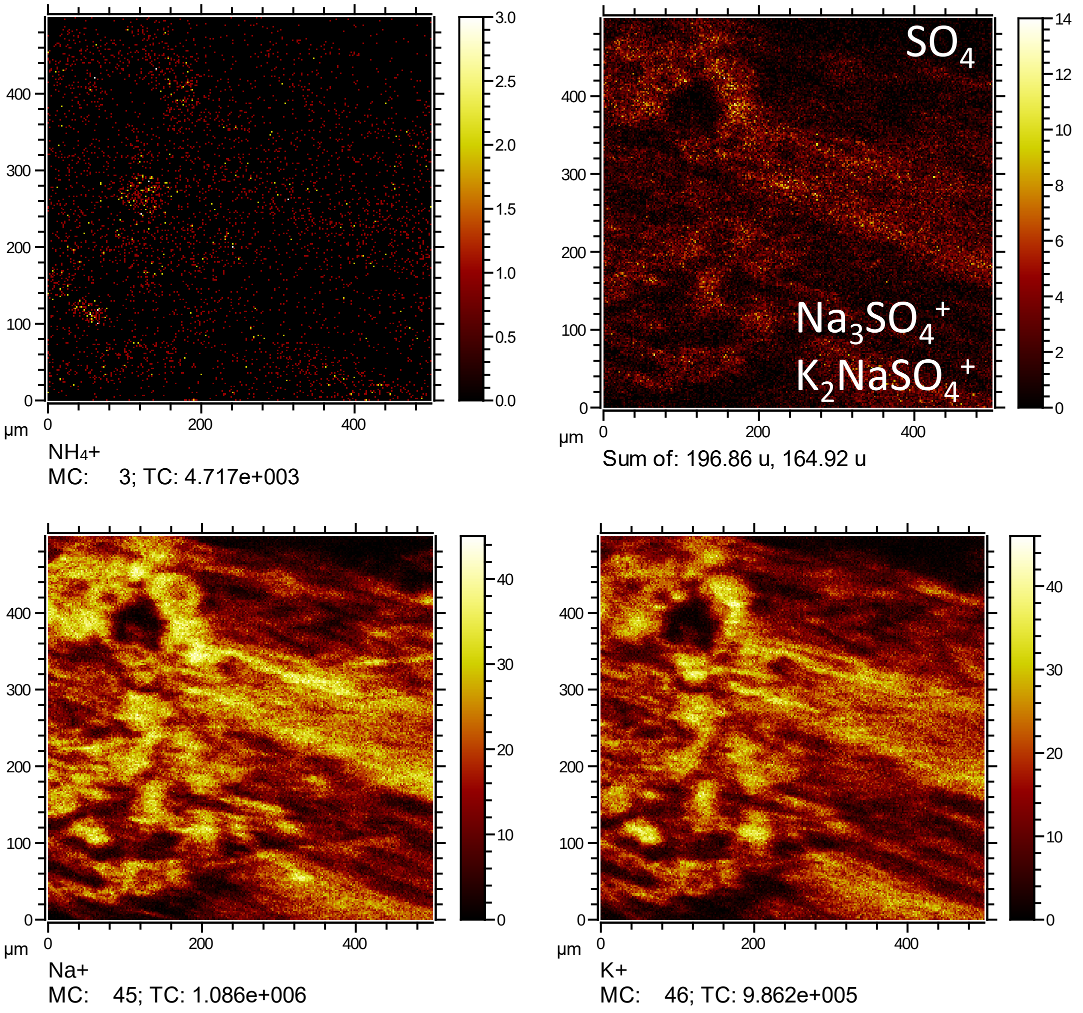
SO4
Na3SO4+
K2NaSO4+
NH4+
MC: 3; TC: 4.717e+003
Sum of: 196.86 u, 164.92 u
Na+
MC: 45; TC: 1.086e+006
K+
MC: 46; TC: 9.862e+005
µm

Fig. S5

Distribution of NH4+ adducts and sphingolipid fragments in TA1, TA2 and TA3

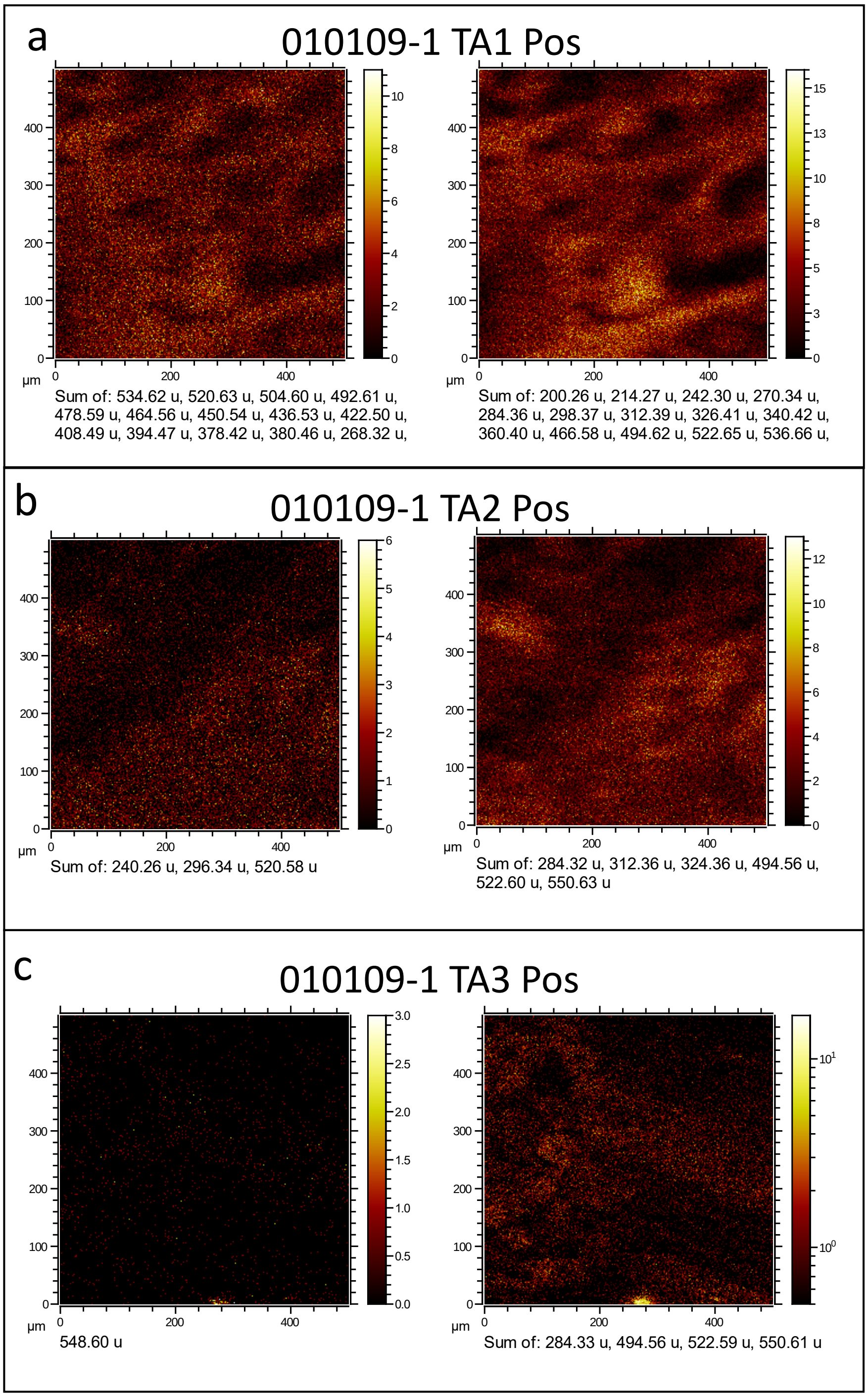

Fig. S6

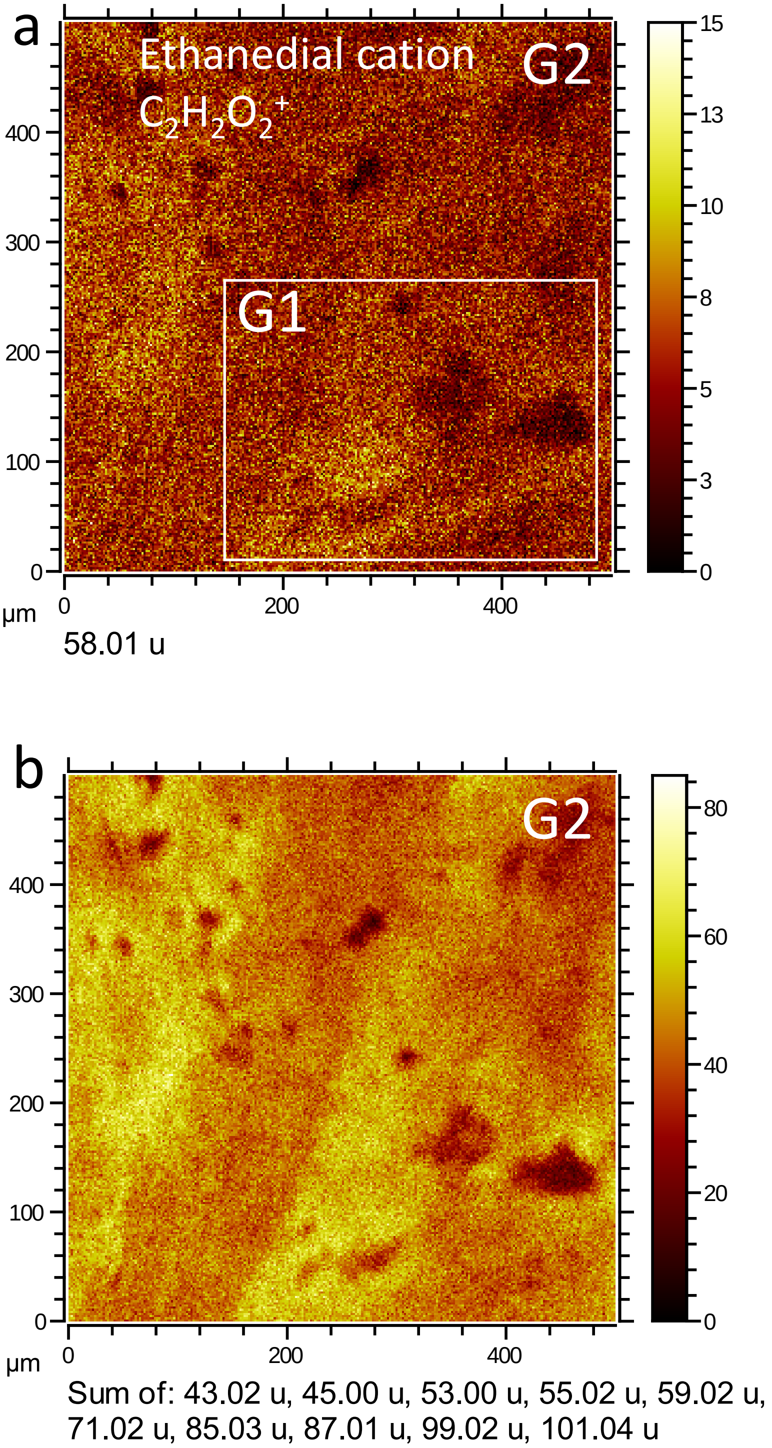
a
Ethanedial cation
$C_2H_2O_2^+$
G2
G1
400
300
200
100
0
µm
0
200
400
15
13
10
8
5
3
0
58.01 u
b
G2
400
300
200
100
0
µm
0
200
400
80
60
40
20
0
Sum of: 43.02 u, 45.00 u, 53.00 u, 55.02 u, 59.02 u, 71.02 u, 85.03 u, 87.01 u, 99.02 u, 101.04 u

Fig. S7

Supplementary Fig. distribution of propyl and butyl fragments in samples 010109-1 and BH-24c

Propyl/isopropyl ($C_3H_7^+$ peak at 43.06)

010109-1 TA1 010109-1 TA2 010109-1 TA3

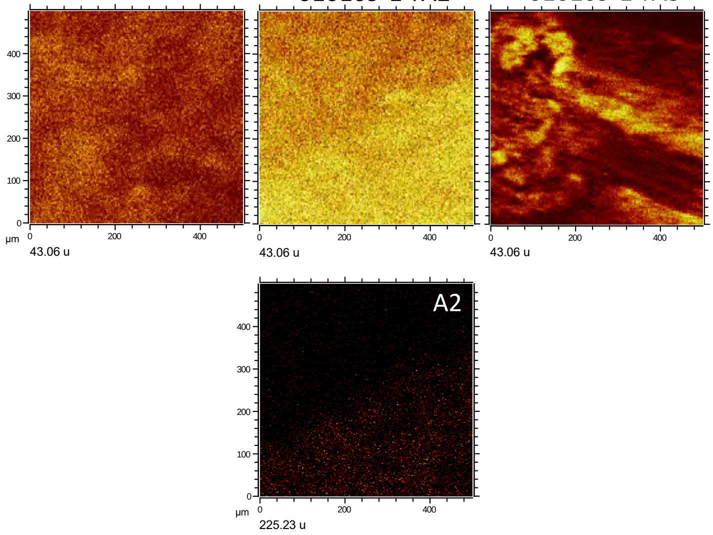

Butyl/isobutyl ($C_4H_9^+$ peak at 57.07)

010109-1 TA1 010109-1 TA2 010109-1 TA3

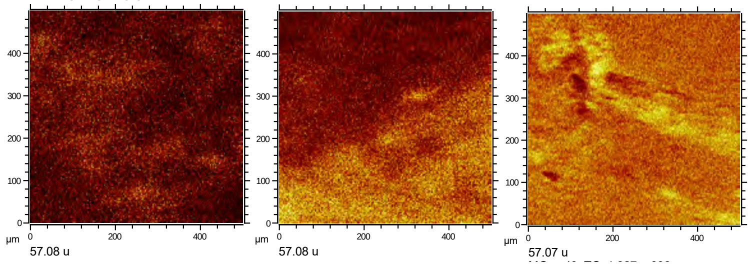

Fig. S8

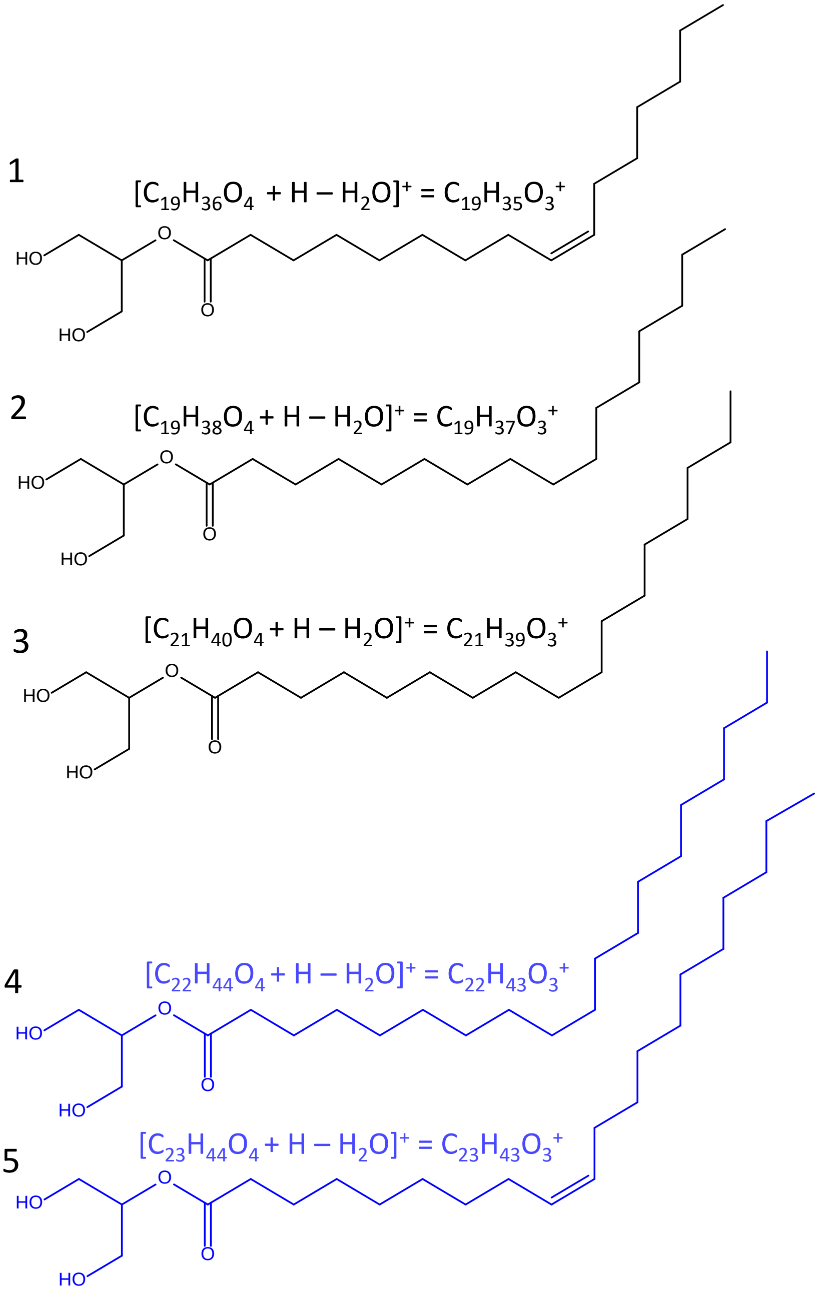

1

$[C_{19}H_{36}O_4 + H - H_2O]^+ = C_{19}H_{35}O_3^+$

2

$[C_{19}H_{38}O_4 + H - H_2O]^+ = C_{19}H_{37}O_3^+$

3

$[C_{21}H_{40}O_4 + H - H_2O]^+ = C_{21}H_{39}O_3^+$

4

$[C_{22}H_{44}O_4 + H - H_2O]^+ = C_{22}H_{43}O_3^+$

5

$[C_{23}H_{44}O_4 + H - H_2O]^+ = C_{23}H_{43}O_3^+$

Fig. S9

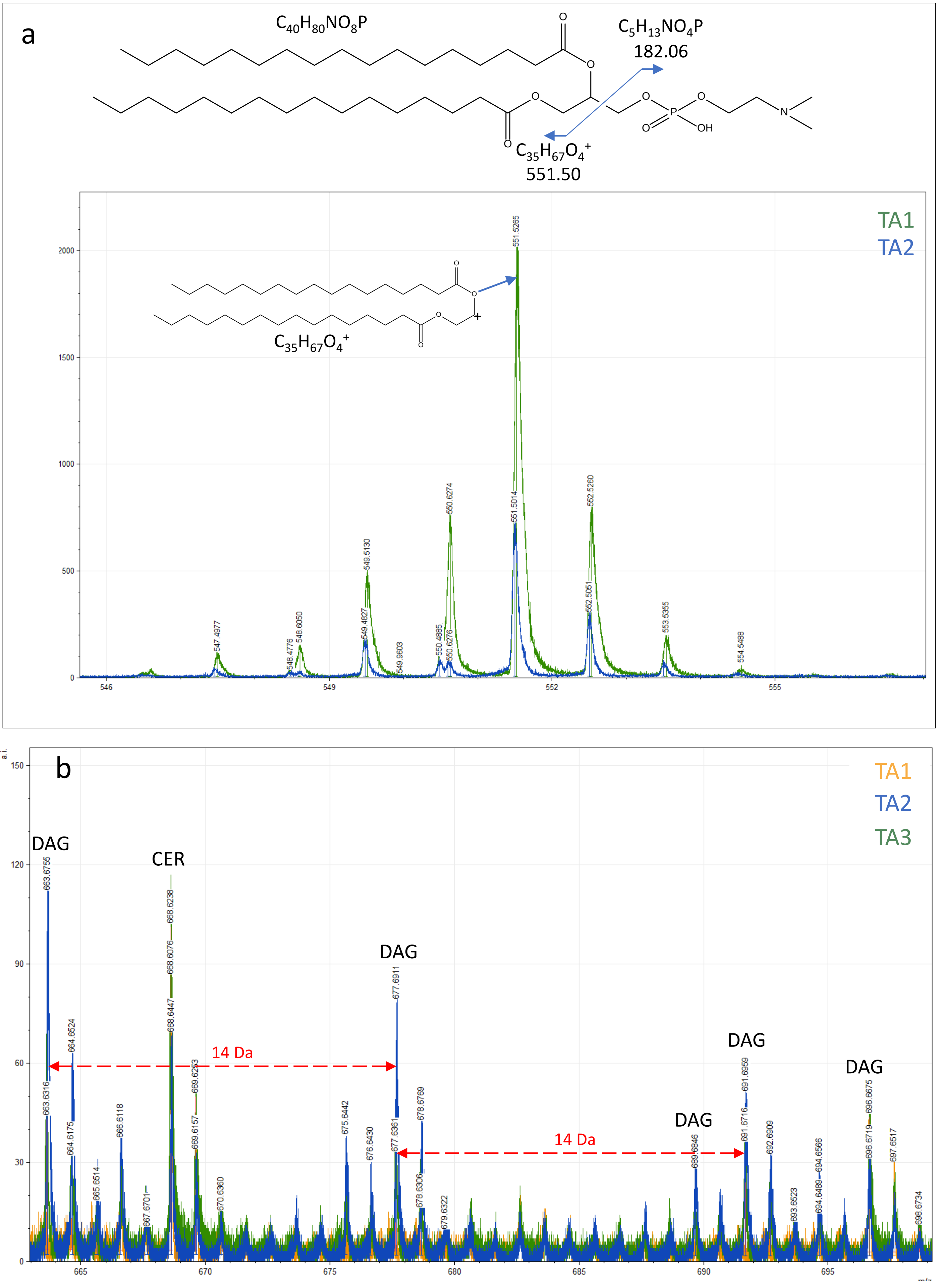
a
$C_{40}H_{80}NO_8P$
$C_5H_{13}NO_4P$
182.06
$C_{35}H_{67}O_4^+$
551.50
TA1
TA2
551.5265
550.6274
549.5130
552.5260
552.5051
551.5014
553.5355
547.4977
548.6050
548.4776
549.4827
549.9603
550.4885
550.6276
554.5488
0
500
1000
1500
2000
546
549
552
555
b
TA1
TA2
TA3
a.i.
DAG
CER
14 Da
663.6755
663.6316
664.6524
664.6175
665.6514
666.6118
667.6701
668.6238
668.6076
668.6447
669.6253
669.6157
670.6360
675.6442
676.6430
677.6911
677.6361
678.6769
678.6306
679.6322
689.6846
691.6959
691.6716
692.6909
693.6523
694.6566
694.6489
696.6675
696.6719
697.6517
698.6734
0
30
60
90
120
150
665
670
675
680
685
690
695

Fig. S10

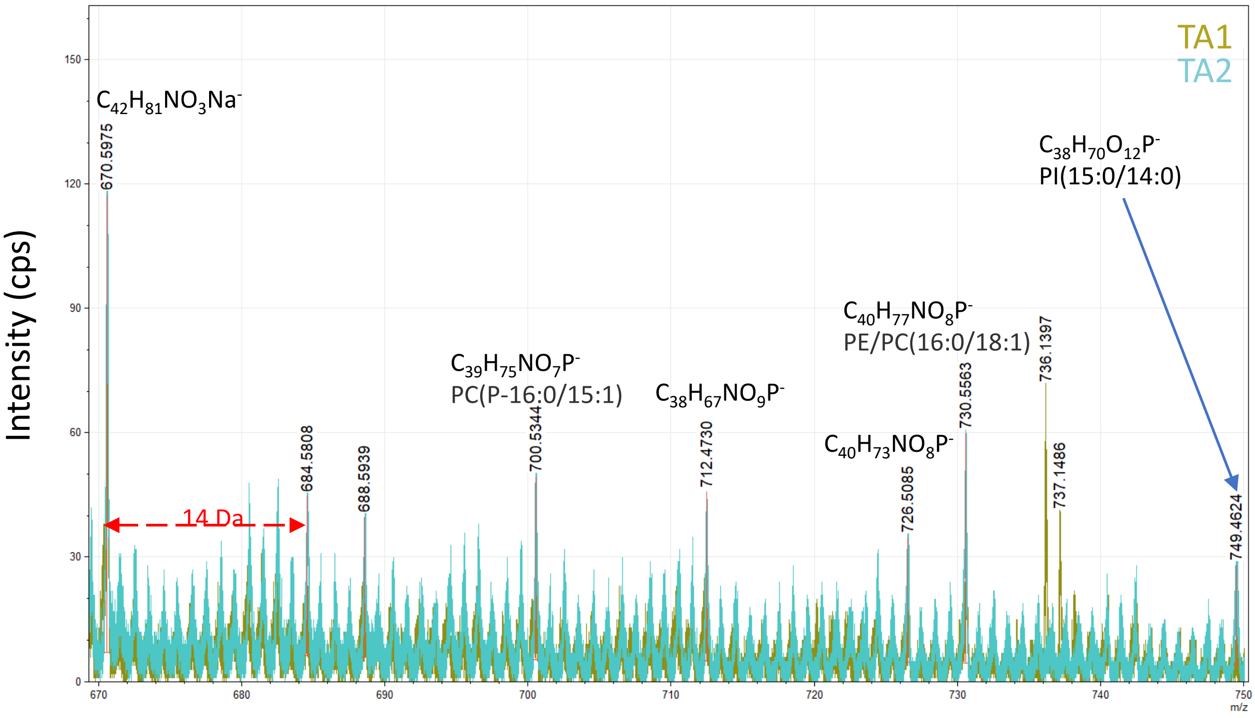
TA1
TA2
Intensity (cps)
m/z
$C_{42}H_{81}NO_3Na^-$
670.5975
14 Da
684.5808
688.5939
$C_{39}H_{75}NO_7P^-$
PC(P-16:0/15:1)
700.5344
$C_{38}H_{67}NO_9P^-$
712.4730
$C_{40}H_{73}NO_8P^-$
726.5085
$C_{40}H_{77}NO_8P^-$
PE/PC(16:0/18:1)
730.5563
736.1397
737.1486
$C_{38}H_{70}O_{12}P^-$
PI(15:0/14:0)
749.4624

Fig. S11

$C_{39}H_{78}NO_5^+$
640.59 m/z⁺

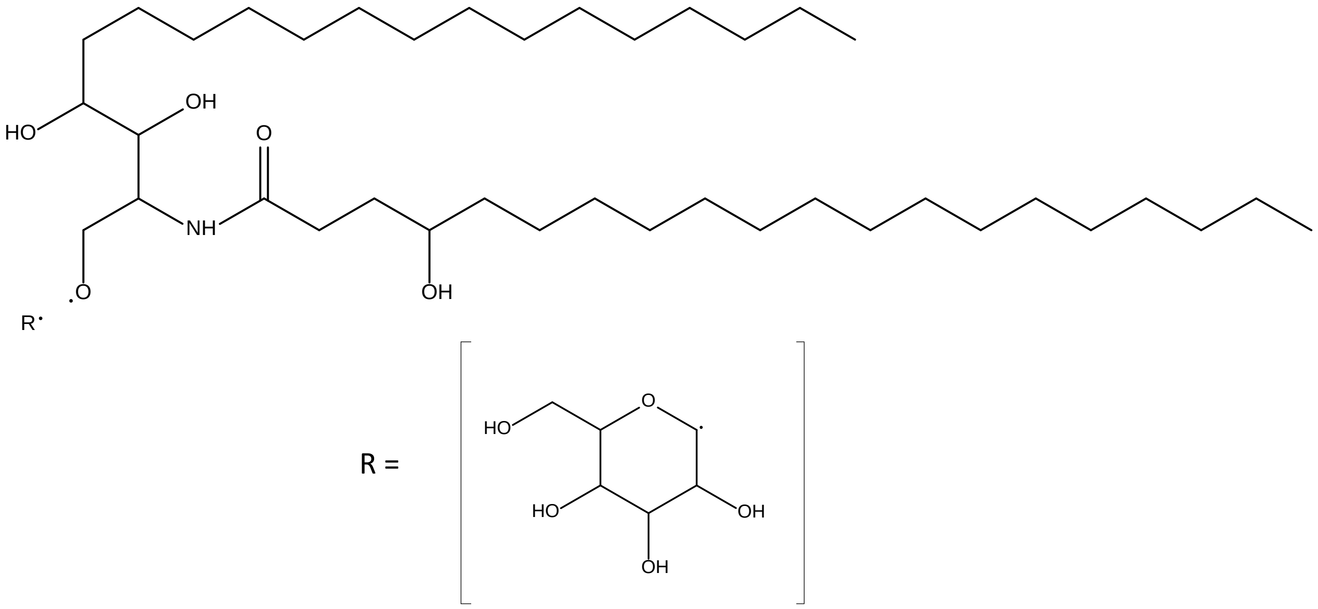

Fig. S12

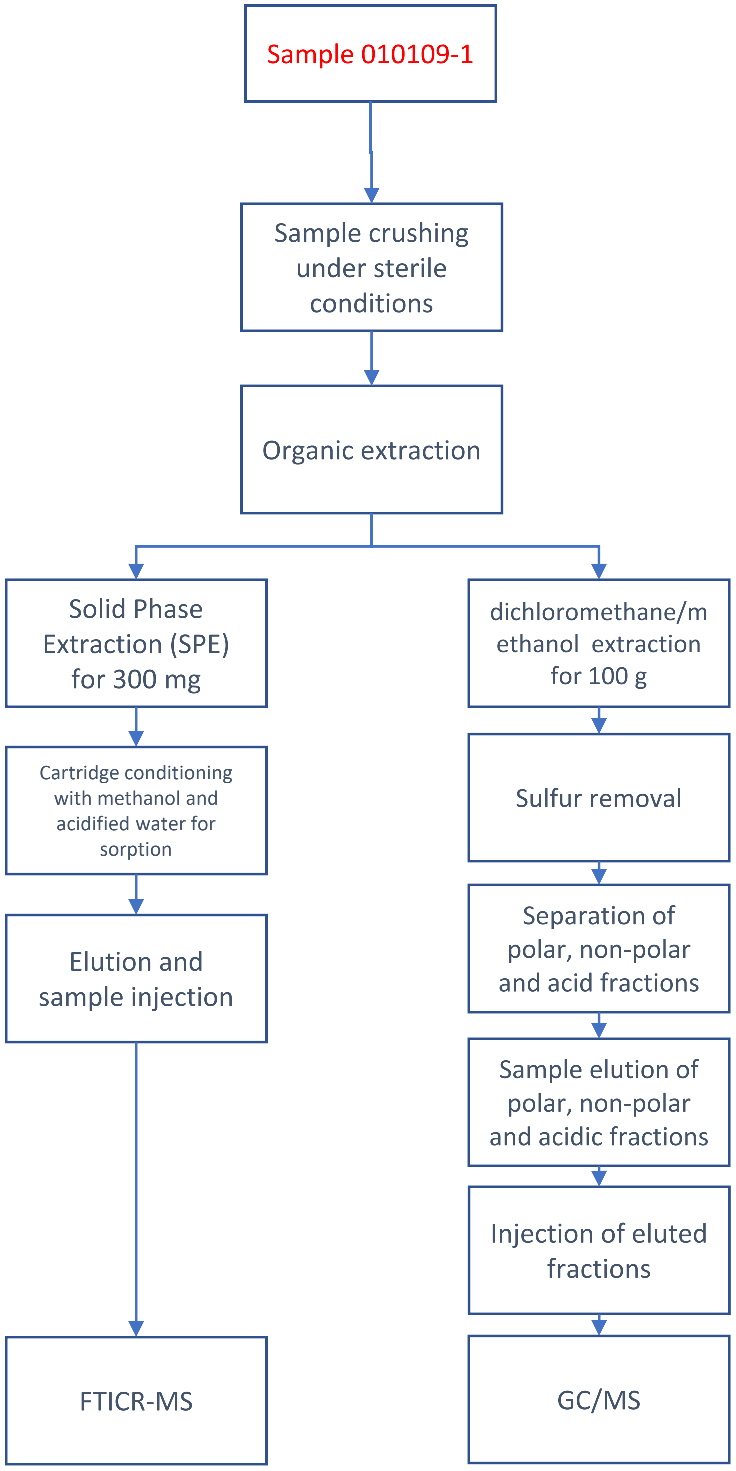
Sample 010109-1
Sample crushing under sterile conditions
Organic extraction
Solid Phase Extraction (SPE) for 300 mg
Cartridge conditioning with methanol and acidified water for sorption
Elution and sample injection
FTICR-MS
dichloromethane/methanol extraction for 100 g
Sulfur removal
Separation of polar, non-polar and acid fractions
Sample elution of polar, non-polar and acidic fractions
Injection of eluted fractions
GC/MS

Fig. S13

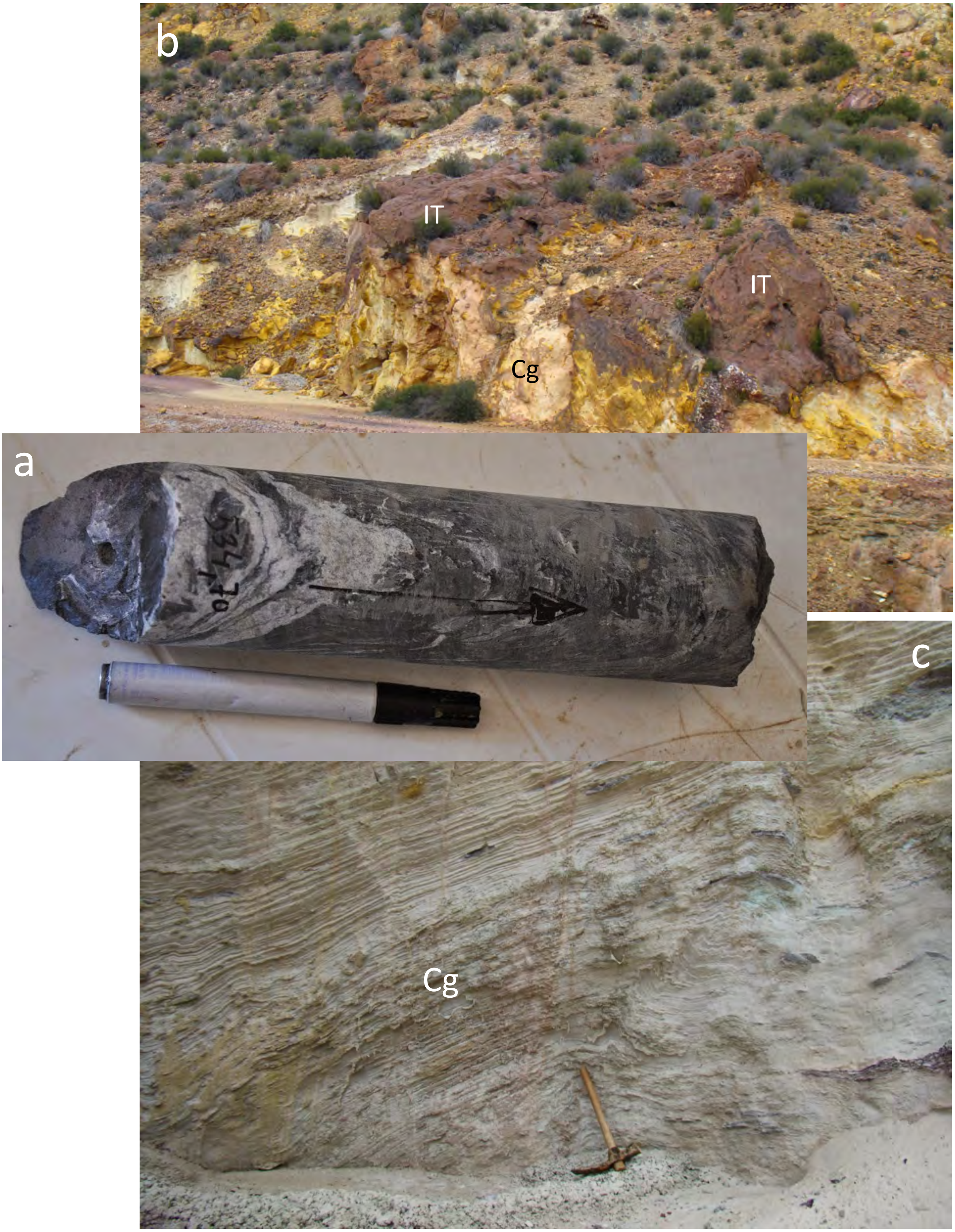

Fig. S14

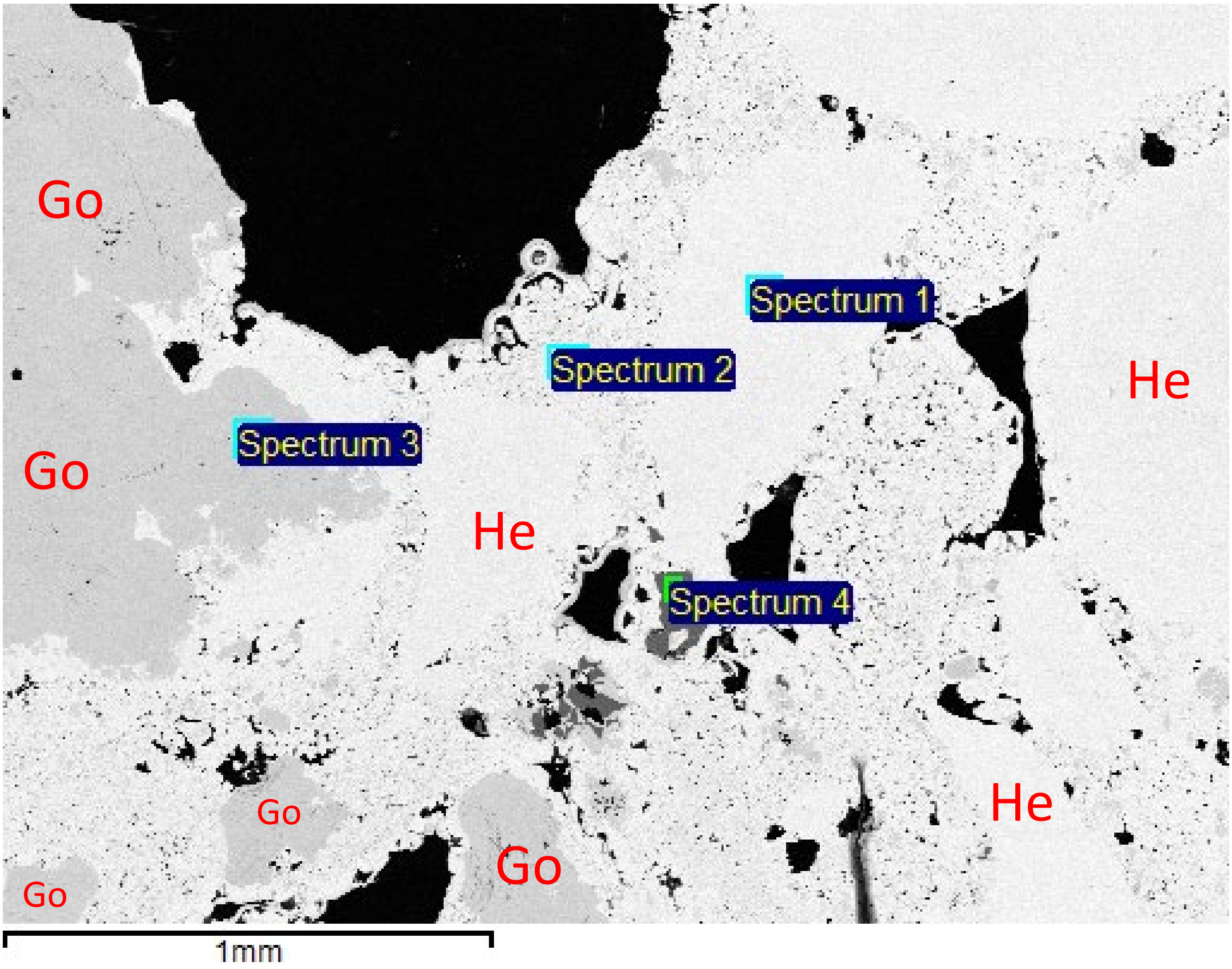
Go
Go
Go
Go
Go
Go
He
He
He
Spectrum 1
Spectrum 2
Spectrum 3
Spectrum 4
1mm

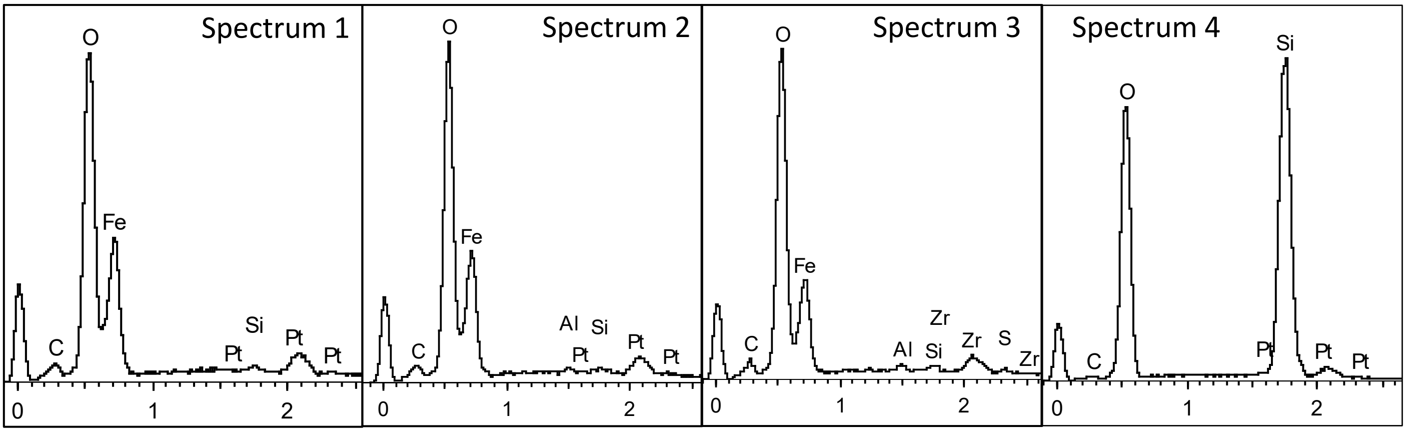
Spectrum 1
O
Fe
C
Si
Pt
Pt
Pt
0
1
2
Spectrum 2
O
Fe
C
Al
Si
Pt
Pt
Pt
0
1
2
Spectrum 3
O
Fe
C
Zr
Al
Si
Zr
S
Zr
0
1
2
Spectrum 4
Si
O
C
Pt
Pt
Pt
0
1
2

Fig. S15

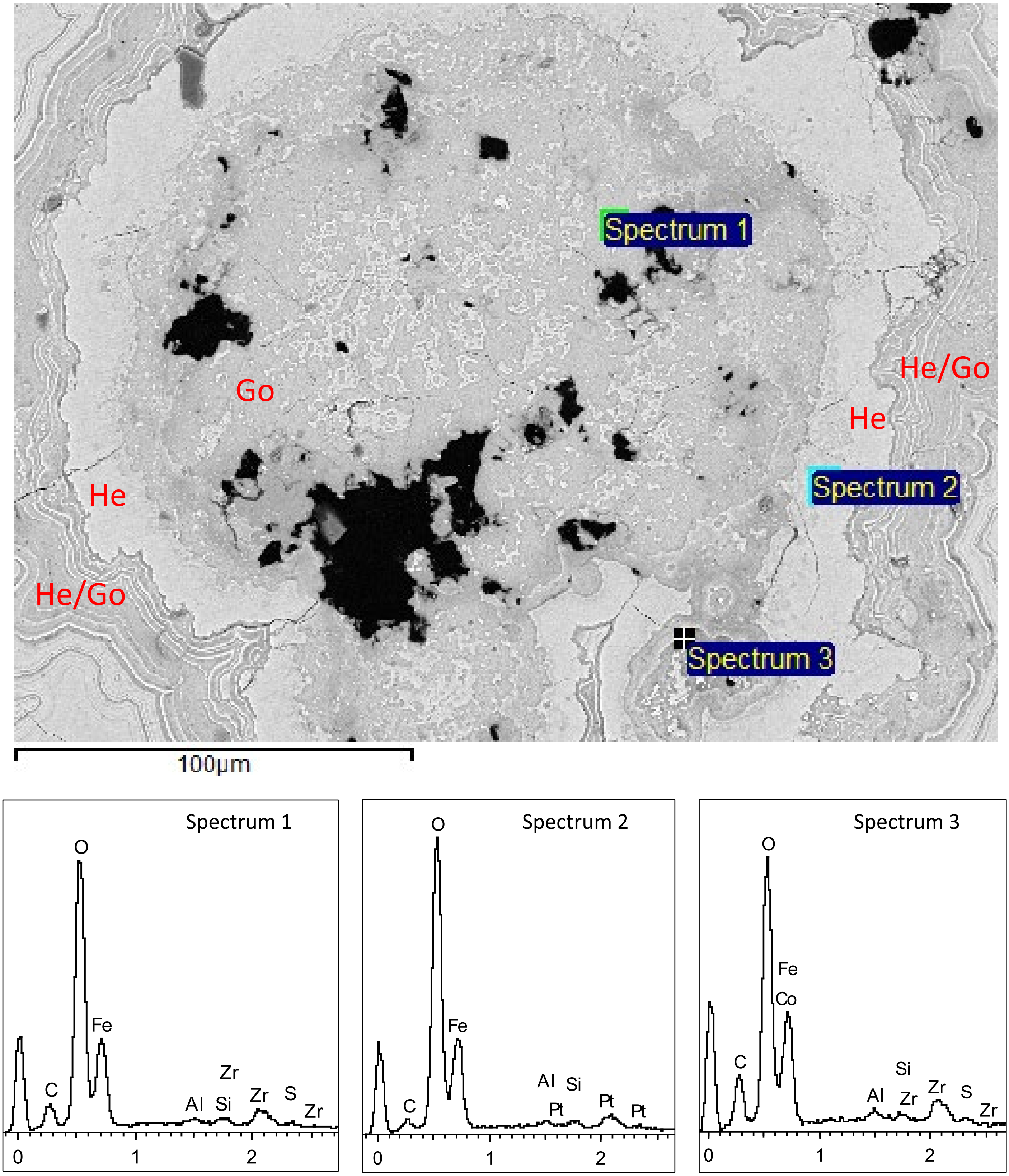
Spectrum 1
Go
He
He/Go
He/Go
He
Spectrum 2
Spectrum 3
100µm
Spectrum 1
O
Fe
C
Zr
Al
Si
Zr
S
Zr
0
1
2
Spectrum 2
O
Fe
C
Al
Si
Pt
Pt
Pt
0
1
2
Spectrum 3
O
Fe
Co
C
Si
Al
Zr
Zr
S
Zr
0
1
2

Fig. S16

NON-POLAR FRACTION

Straight chain or *normal* alkanes	Compound formula
n-Undecane	$C_{11}H_{24}$
n-Dodecane	$C_{12}H_{26}$
n-Tridecane	$C_{13}H_{28}$
n-Tetradecane	$C_{14}H_{30}$
n-Pentadecane	$C_{15}H_{32}$
n-Hexadecane	$C_{16}H_{34}$
n-Heptadecane	$C_{17}H_{36}$
n-Octadecane	$C_{18}H_{38}$
n-Nonadecane	$C_{19}H_{40}$
n-Eicosane	$C_{20}H_{42}$
n-Henicosane	$C_{21}H_{44}$
n-Docosane	$C_{22}H_{46}$
n-Tricosane	$C_{23}H_{48}$
n-Tetracosane	$C_{24}H_{50}$
n-Pentacosane	$C_{25}H_{52}$
n-Hexacosane	$C_{26}H_{54}$
n-Heptacosane	$C_{27}H_{56}$
n-Octacosane	$C_{28}H_{58}$
n-Nonacosane	$C_{29}H_{60}$
n-Triacontane	$C_{30}H_{62}$
n-Hentriacontane	$C_{31}H_{64}$

Alkenes and branched alkanes	Compound formula
Undecane, 4-methyl-	$C_{12}H_{26}$
Tridecene	$C_{13}H_{26}$
Tetradecene	$C_{14}H_{28}$
Tridecane, 3-methyl-	$C_{14}H_{30}$
Dodecane, 2,6,11-trimethyl-	$C_{15}H_{32}$
Hexadecene	$C_{16}H_{32}$
5,5-Diethyltridecane	$C_{17}H_{36}$
Heptadecane, 2-methyl-	$C_{18}H_{38}$
Heptadecane, 4-methyl-	$C_{18}H_{38}$
Pentadecane, 2,6,10,14-tetramethyl- (Pristane)	$C_{19}H_{40}$
Hexadecane, 2,6,10,14-tetramethyl (Phytane)	$C_{20}H_{42}$
Hexadecane, 2,6,11,15-tetramethyl-	$C_{20}H_{42}$

POLAR FRACTION

Linear alcohols	TMS Derivative formula	Compound formula
1-Undecanol, TMS derivative	$C_{14}H_{32}OSi$	$C_{11}H_{24}O$
1-Dodecanol, TMS derivative	$C_{15}H_{34}OSi$	$C_{12}H_{26}O$
3-Dodecanol, 3,7,11-trimethyl-	-	$C_{15}H_{32}O$
1-Tridecanol, TMS derivative	$C_{16}H_{36}OSi$	$C_{13}H_{28}O$
1-Tetradecanol, TMS derivative	$C_{17}H_{38}OSi$	$C_{14}H_{30}O$
1-Pentadecanol, TMS derivative	$C_{18}H_{40}OSi$	$C_{15}H_{32}O$
1-Hexadecanol, TMS derivative	$C_{19}H_{42}OSi$	$C_{16}H_{34}O$
1-Heptadecanol, TMS derivative	$C_{20}H_{44}OSi$	$C_{17}H_{36}O$
1-Octadecanol, TMS derivative	$C_{21}H_{46}OSi$	$C_{18}H_{38}O$
18-Methyl-nonadecanol, trimethylsilyl ether	$C_{23}H_{50}OSi$	$C_{20}H_{42}O$
Docosanol, TMS derivative	$C_{25}H_{54}OSi$	$C_{22}H_{46}O$
1-Tetracosanol, TMS derivative	$C_{27}H_{58}OSi$	$C_{24}H_{50}O$
1-O-hexadecylglycerol - bis-trimethylsilyl ether derivative	$C_{25}H_{56}O_3Si_2$	$C_{19}H_{40}O_3$
1-O-Heptadecylglycerol, bis-trimethylsilyl ether	$C_{26}H_{58}O_3Si_2$	$C_{20}H_{42}O_3$
Glycerol monostearate, 2TMS derivative	$C_{27}H_{58}O_4Si_2$	$C_{21}H_{42}O_4$

POLAR FRACTION (cont)

Branched Alkanols	TMS Derivative formula	Compound formula
3-Dodecanol, 3,7,11-trimethyl-	-	$C_{15}H_{32}O$
18-Methyl-nonadecanol, TMS derivative	$C_{23}H_{50}OSi$	$C_{20}H_{42}O$
Rhodopin*	-	$C_{40}H_{58}O$

*Main carotenoid pigment in phototrophic bacteria

Steroids and hopanoids	TMS Derivative formula	Compound formula
Stigmasta-3,5-dien-7-one	-	$C_{29}H_{46}O$
24,25-Dihydroxyvitamin D/24,25-Dihydroxycholecalciferol	-	$C_{27}H_{44}O_3$
(Dihydro)Cholesterol, TMS derivative	$C_{30}H_{54}OSi$	$C_{27}H_{46}O$
Cholestan-3-ol, TMS derivative	$C_{30}H_{56}OSi$	$C_{27}H_{48}O$
β-Sitosterol TMS derivate	$C_{32}H_{58}OSi$	$C_{29}H_{50}O$
Stigmastanol, TMS derivative	$C_{32}H_{60}OSi$	$C_{29}H_{52}O$
7,8-Epoxylanostan-11-ol, 3-acetoxy-	-	$C_{32}H_{54}O_4$
25-Hydroxycholesterol TMS derivative	$C_{33}H_{62}O_2Si_2$	$C_{27}H_{46}O_2$
Ergost-5-ene-3,25-diol TMS derivative	$C_{34}H_{64}O_2Si_2$	$C_{28}H_{48}O_2$
Stigmasterol, TMS derivate	$C_{32}H_{56}OSi$	$C_{29}H_{48}O$
Lupan-3-ol, acetate	-	$C_{30}H_{52}O$

ACIDIC FRACTION

Fatty acids as methyl esters	Methyl ester/TMS derivative formula	Compound formula
Undecanoic acid, methyl ester	$C_{12}H_{24}O_2$	$C_{11}H_{22}O_2$
Dodecanoic acid, methyl ester	$C_{13}H_{26}O_2$	$C_{12}H_{24}O_2$
Tridecanoic acid, methyl ester	$C_{14}H_{28}O_2$	$C_{13}H_{26}O_2$
Tetradecanoic acid, methyl ester	$C_{15}H_{30}O_2$	$C_{14}H_{28}O_2$
Pentadecanoic acid, methyl ester	$C_{16}H_{32}O_2$	$C_{15}H_{30}O_2$
Hexadecanoic acid, methyl ester	$C_{17}H_{34}O_2$	$C_{16}H_{32}O_2$
10-Heptadecenoic acid (Z)-, methyl ester	$C_{18}H_{34}O_2$	$C_{17}H_{32}O_2$
Heptadecanoic acid, methyl ester	$C_{18}H_{36}O_2$	$C_{17}H_{34}O_2$
Octadecanoic acid, methyl ester	$C_{19}H_{38}O_2$	$C_{18}H_{36}O_2$
Nonadecanoic acid, methyl ester	$C_{20}H_{40}O_2$	$C_{19}H_{38}O_2$
Eicosanoic acid, methyl ester	$C_{21}H42O_2$	$C_{20}H_{40}O_2$
Heneicosanoic acid, methyl ester	$C_{22}H_{44}O_2$	$C_{21}H_{42}O_2$
Docosanoic acid, methyl ester	$C_{23}H_{46}O_2$	$C_{22}H_{44}O_2$
Tricosanoic acid, methyl ester	$C_{24}H_{48}O_2$	$C_{23}H_{46}O_2$
Tetracosanoic acid, methyl ester	$C_{25}H_{50}O_2$	$C_{24}H_{48}O_2$
Pentacosanoic acid, methyl ester	$C_{26}H_{52}O_2$	$C_{25}H_{50}O_2$
Hexacosanoic acid, methyl ester	$C_{27}H_{54}O_2$	$C_{26}H_{52}O_2$
Heptacosanoic acid, methyl ester	$C_{28}H_{56}O_2$	$C_{27}H_{54}O_2$
Octacosanoic acid, methyl ester	$C_{29}H_{58}O_2$	$C_{28}H_{56}O_2$
Triacontanoic acid, methyl ester	$C_{31}H_{62}O_2$	$C_{30}H_{60}O_2$
**Monounsaturated fatty acids as methyl esters**	**Methyl ester/TMS derivative formula**	**Compound formula**
9-Tetradecenoic acid (Z) acid methyl ester	$C_{15}H_{28}O_2$	$C_{14}H_{26}O_2$
9-Hexadecenoic acid, methyl ester (a.k.a. 16:1 ω7)	$C_{17}H_{32}O_2$	$C_{16}H_{30}O_2$
9-Octadecenoic acid (Z)-, methyl ester (a.k.a. 18:1 ω9)	$C_{19}H_{36}O_2$	$C_{18}H_{34}O_2$
8-Octadecenoic acid, methyl ester (a.k.a. 18:1 ω10)	$C_{19}H_{36}O_2$	$C_{18}H_{34}O_2$
11-Eicosenoic acid, methyl ester (a.k.a. 20:1 ω8)	$C_{21}H_{40}O_2$	$C_{20}H_{38}O_2$
**Dicarboxylic fatty acids as methyl esters**	**Methyl ester/TMS derivative formula**	**Compound formula**
Octanedioic acid, dimethyl ester	$C_{10}H_{18}O_4$	$C_8H_{14}O_4$
Decanedioic acid, dimethyl ester	$C_{12}H_{22}O_4$	$C_{10}H_{20}O_3$

Branched acids as methyl esters	Methyl ester derivative formula	Compound formula
11-methyl-dodecanoic acid, methyl ester (a.k.a. *i*-13:0)	$C_{14}H_{28}O_2$	$C_{13}H_{26}O_2$
Dodecanoic acid, 10-methyl-, methyl ester (a.k.a. *a*-13:0)	$C_{14}H_{28}O_2$	$C_{13}H_{26}O_2$
Tridecanoic acid, 12-methyl-, methyl ester (a.k.a. *i*-14:0)	$C_{15}H_{30}O_2$	$C_{14}H_{28}O_2$
13-Methyltetradec-9-enoic acid, methyl ester (a.k.a. *i*-15:1)	$C_{16}H_{30}O_2$	$C_{15}H_{28}O_2$
Tetradecanoic acid, 12-methyl-, methyl ester (a.k.a. a-15:0)	$C_{16}H_{32}O_2$	$C_{15}H_{30}O_2$
11-cyclohexylundecanoic acid, methyl ester	$C_{18}H_{34}O_2$	$C_{17}H_{32}O_2$
Hexadecanoic acid, 15-methyl-, methyl ester (a.k.a. *i*-17:0)	$C_{18}H_{36}O_2$	$C_{17}H_{34}O_2$
Cyclopropaneoctanoic acid, 2-hexyl-, methyl ester	$C_{20}H_{38}O_2$	$C_{19}H_{38}O_2$
22-methyl-tetracosanoic acid, methyl ester (a.k.a. *a*-25:0)	$C_{26}H_{52}O_2$	$C_{25}H_{50}O_2$

Table S1. List of organic compounds (lipids) identified in the Upper Gossan sample of Peña de Hierro through GC/MS analysis. TMS means trimethylsylil. *i-/a-* refer to *iso-/anteiso-* configuration of branched fatty acids (i.e. fatty acids with methyl groups at ultimate or penultimate position in the acid chain, respectively). Z stands for cis configuration.

Sample	Group	m/z^+	m/z^-	Potential compounds	
				m/z^+	m/z^-
010109-1	Group 1	NH_4^+-bearing positive adducts which have maximum at 494.56, 522.59 and 550.62 corresponding to $C_{34}H_{72}N^+$ ($C_{34}H_{68}$), $C_{36}H_{76}N^+$ ($C_{36}H_{72}$), and $C_{38}H_{80}N^+$ ($C_{38}H_{74}$)	Fatty acid [M - H]$^-$ ions like 197.15, 199.17, 227.20, or 255.23, corresponding to $C_{12}H_{21}O_2^-$, $C_{12}H_{23}O_2^-$, $C_{14}H_{27}O_2^-$, and $C_{16}H_{31}O_2^-$	Lineal hydrocarbons fragmented and ionized from long lipids which form adducts with NH_4^+ likely sphingolipids	Fatty acids coming from lipidic structures like ceramides
		Inorganic salts bearing sulfate groups like $Na_3SO_4^+$, and $K_2NaSO_3^+$	fragments bearing SO_3 and SO_4 groups as $C_8H_7SO_3^-$ (183.01), $C_{12}H_{25}SO_4^-$ (265.15), $C_{14}H_{29}SO_4^-$ (293.17), $C_{19}H_{31}SO_3^-$ (339.18), and $C_{27}H_{45}SO_4^-$ (465.30)	Salt composition of mineralized structures	SOx-bearing adducts of cholesterol-like sterols and lipids coming from the mineralized surface of fungal structures
	Group 2	31.02, 47.01, 61.03, 69.03, 85.03, 87.05 corresponding to cations CH_3O^+, $CH_3O_2^+$, $C_2H_5O_2^+$, $C_4H_5O^+$, $C_4H_5O_2^+$, and $C_4H_7O_2^+$	43.02, 53.00, 55.02, 58.01, 59.02, 67.02, 69.03, 71.02, 83.01, 85.03, 87.01, 99.01, and 101.03, which meet anions at $C_2H_3O^-$, C_3HO^-, $C_3H_3O^-$, $C_2H_2O_2^-$, $C_2H_3O_2^-$, $C_4H_3O^-$, $C_4H_5O^-$, $C_3H_3O_2^-$, $C_4H_3O_2^-$, $C_4H_5O_2^-$, $C_3H_3O_3^-$, $C_4H_3O_3^-$, and $C_4H_5O_3^-$	Likely terpenoid fragments sourced in plants	
	Group 3	m/z^+ peaks at 28.03, 42.04, 43.06, 44.05, 56.06, 84.08, 86.07, 86.10, 110.08, 111.09, 112.09, 122.08, 123.09, 124.09, 125.11, 126.11, 135.09, 136.09, 137.11, 138.11, 308.31, and 310.33, which match CH_2N^+, $C_2H_4N^+$, $C_3H_6^+$, $C_2H_6N^+$, $C_4H_8^+$/$C_3H_6N^+$, $C_5H_{10}N^+$, $C_3H_8N_3^+$, $C_5H_{12}N^+$, $C_6H_{10}N_2^+$, $C_6H_{11}N_2^+$, $C_5H_{10}N_3^+$, $C_6H_8N_3^+$, $C_7H_{11}N_2^+$, $C_6H_{10}N_3^+$, $C_7H_{13}N_2^+$, $C_7H_{14}N_2^+$, $C_8H_{11}N_2^+$, $C_7H_{10}N_3^+$, $C_8H_{13}N_2^+$, and $C_8H_{14}N_2^+$, $C_{20}H_{38}NO^+$, and $C_{20}H_{40}NO^+$.	91.02, 92.02, 93.04, 94.03, 117.03, 211.06, and 212.07, which meet well molecular fragments like $C_4HN_3^-$, $C_4H_2N_3^-$, $C_5H_5N_2^-$, $C_4H_4N_3^-$, $C_4H_5O_4^-$, $C_{13}H_9NO_2^-$, and $C_{13}H_{10}NO_2^-$,	Fragments of amino acids and peptides	
	Group 4	positive ions at 30.04, 178.05, 179.07, 180.05, 209.08, 211.09, and 219.18, which fit the m/z^+ fragments CH_4N^+, $C_9H_8NO_3^+$, $C_8H_9N_3O_2^+$, $C_{12}H_6NO^+$, $C_{14}H_{11}NO^+$, $C_{13}H_{11}N_2O^+$, and $C_{11}H_{25}NO_3^+$.	n/a	Unknown origin	n/a
	Group 5	m/z^+ ions at 76.03, 77.04, 104.03, 105.03, 149.02, and 150.03 meeting $C_6H_4^+$, $C_6H_5^+$, $C_7H_4O^+$, $C_7H_5O^+$, $C_8H_5O_3^+$, and $C_8H_6O_3^+$	negative ions at 73.03, 77.04, 105.04, 120.02, and 121.03 ($C_3H_5O_2^-$, $C_6H_5^-$, $C_7H_5O^-$, $C_7H_4O_2^-$, and $C_7H_5O_2^-$)	Plant terpenoids like piperonal derivatives	
	Group 6	m/z^+ at 166.04, and 168.04 matching $C_5H_{10}O_6^+$, and $C_8H_8O_4^+$	n/a	Vanillic acid derivatives	n/a
	Group 7	Unique positive fragment at 57.03 ($C_3H_5O^+$)	Negative fragments at 279.16, and 280.16 ($C_{16}H_{23}O_4^-$, and $C_{16}H_{24}O_4^-$)	Plant terpenoids	
	Group 8	n/a	m/z^- sets at 666.06, and 736.14	n/a	Tannins, or some polysaccharides
	Group 9	Positive ions with mass higher than 500 Da like 239.25, 281.07, 313.30, 367.32, 495.47, 523.50, 537.52, 565.53, 579.56, 593.58, and 649.63, which are assigned to $C_{16}H_{31}O^+$, $C_{17}H_{13}O_4^+$, $C_{19}H_{37}O_3^+$, $C_{23}H_{43}O_3^+$, $C_{31}H_{59}O_4^+$, $C_{33}H_{63}O_4^+$, $C_{35}H_{67}O_4^+$, $C_{37}H_{71}O_4^+$, $C_{38}H_{73}O_4^+$, and $C_{42}H_{81}O_4^+$	m/z^- ions like 227.19, 241.22, 253.21, 255.22, and 269.23 matching well fragments of fatty acids and/or ester carboxylates as $C_{14}H_{27}O_2^-$, $C_{15}H_{29}O_2^-$, $C_{16}H_{29}O_2^-$, $C_{16}H_{31}O_2^-$, and $C_{17}H_{33}O_2^-$	Glycerolipids and glycerophospholipids with possible occurrence of glycerophosphocholines. Fatty acids fragmented from the lipidic structure. Abundant in TA2 and TA3, and secondary in TA1	

Table S2. Characterization of the different morphological groups through the molecular distribution in the Upper Gossan sample of Peña de Hierro that is obtained using the mapping capabilities of the ToF-SIMS.

PAH name	Formula	TA1		TA2		TA3	
		Measured (m/z^+)	Intensity	Measured (m/z^+)	Intensity	Measured (m/z^+)	Intensity
dihydropentalene	C8H7+	103.05	1455	103.05	1010	103.05	1276
indene	C9H7+	115.05	3665	115.05	2013	115.05	1552
naphtalene	C10H8+	128.06	2618	128.06	1350	128.05	1850
tetrahydronaphthalene	C10H11+	131.08	925	131.09	609	131.08	1131
methylnaphtalene	C11H9+	141.06	1414	141.06	726	141.06	2148
acenaphthylene	C12H8+	152.05	999	-	-	152.05	423
fluorene	C13H9+	165.06	1301	165.07	452	165.06	639
phenanthrene/anthracene	C14H10+	178.05	1050	-	-	178.07	215
methylpyrene	C17H11+	215.07	88	215.07	69	215.07	156
cyclopenta-phenanthrene	C19H11+	-	-	-	-	239.08	100
benzoperylene	C22H12+	276.08	30	-	-	276.08	46

Table S3. Positive $[M\text{-}H]^+$ and M^+ ions of different polycyclic aromatic hydrocarbons (PAHs) preserved in the Upper Gossan materials of Peña de Hierro.

Identification of fatty acids by GS/MS as methyl esters	Generic fatty acid (TOF-SIMS)	Calculated [M-H]$^-$	010109-1 - TA1 - Neg			010109-1 - TA2 - Neg			010109-1 - TA3 - Neg		
			Measured (m/z$^+$)/Error/Intensity			Measured (m/z$^+$)/Error/Intensity			Measured (m/z$^+$)/Error/Intensity		
Undecanoic acid, methyl ester*	Undecanoic acid $C_{11:0}$ ($C_{11}H_{22}O_2$)	185.15	-	-	-	-	-	-	185.15	-15.12	543
Dodecanoic acid, methyl ester*	Dodecanoic acid $C_{12:0}$ ($C_{12}H_{24}O_2$)	199.17	199.17	-20.59	168	199.17	-20.08	1074	199.17	-21.09	156
Methyl 11-methyl-dodecanoate*, Dodecanoic acid, 10-methyl-, methyl ester; Tridecanoic acid, methyl ester	Tridecanoic acid $C_{13:0}$ ($C_{13}H_{26}O_2$)	213.19	213.18	-36.12	83	213.18	-42.68	751	213.18	-37.53	101
	Myristoleic acid $C_{14:1}$ ($C_{14}H_{26}O_2$)	225.19	225.18	-36.20	95	225.17	-88.81	1604	225.18	-42.63	194
Myristoleic acid methyl ester; Tridecanoic acid, 12-methyl-, methyl ester	Myristic acid $C_{14:0}$ ($C_{14}H_{28}O_2$)	227.20	**227.19**	**-37.70**	**465**	**227.19**	**-47.10**	**8194**	**227.20**	-18.93	**767**
	Pentadecenoic acid $C_{15:1}$ ($C_{15}H_{28}O_2$)	239.20	239.20	-37.70	80	239.20	-8.36	1350	239.20	10.87	126
13-Methyltetradec-9-enoic acid; Pentadecanoic acid; 12-methyltetradecanoic acid	Pentadecanoic/methyl myristic acid $C_{15:0}$ ($C_{15}H_{30}O_2$)	241.22	**241.22**	**2.07**	**420**	**241.22**	**-5.39**	**6154**	**241.21**	-22.80	**283**
Methyl hexadec-9-enoate	Palmitoleic acid $C_{16:1}$ ($C_{16}H_{30}O_2$)	253.22	**253.20**	**-78.98**	**272**	**253.21**	**-48.97**	**4709**	**253.21**	-26.06	**271**
Hexadecanoic acid, methyl ester; Methyl hexadec-9-enoate	Palmitic acid $C_{16:0}$ ($C_{16}H_{32}O_2$)	255.23	**255.23**	**3.13**	**900**	**255.22**	**-44.27**	**15668**	**255.23**	-11.75	**1250**
	Methyl palmytic/Heptadecenoic acid $C_{17:1}$ ($C_{17}H_{32}O_2$)	267.23	267.23	-1.50	60	267.22	-30.31	1040	267.23	-11.60	113
Cyclopropaneoctanoic acid, 2-hexyl-, methyl ester; Hexadecanoic acid, 15-methyl-, methyl ester	Heptadecanoic acid $C_{17:0}$ ($C_{17}H_{34}O_2$)	269.25	269.25	4.09	199	269.23	-65.00	3090	269.24	-18.94	257
8-/9-Octadecenoid acid	Oleic acid $C_{18:1}$ ($C_{18}H_{34}O_2$)	281.25	281.24	-29.70	71	281.24	-52.27	2241	281.24	-22.04	100
Octadecanoic acid, methyl ester	Stearic acid $C_{18:0}$ ($C_{18}H_{36}O_2$)	283.26	283.27	18.30	149	283.25	-41.30	3567	283.26	0.35	225
Nonadecanoic acid, methyl ester	Nonadecanoic acid $C_{19:1}$ ($C_{19}H_{36}O_2$)	295.27	-	-	-	295.25	-60.96	267	295.26	-19.30	48
	Nonadecyl acid/methyl stearate; nonadecanoic acid $C_{19:0}$ ($C_{19}H_{38}O_2$)	297.28	297.26	-54.83	43	297.26	-74.68	411	297.28	-10.09	37
	Eicosapentanoic acid $C_{20:5}$ ($C_{20}H_{30}O_2$)	301.22	301.20	-46.20	269	301.21	-19.59	121	-	-	-
	Arachinodic acid $C_{20:4}$ ($C_{20}H_{32}O_2$)	303.23	303.21	-75.70	156	303.21	-75.85	124	-	-	-
11-Eicosenoic acid, methyl ester	Gondoic acid $C_{20:1}$ ($C_{20}H_{38}O_2$)	309.27	309.28	38.48	33	309.27	-6.79	210	309.27	12.29	61
Eicosanoic acid, methyl ester	Arachidic acid $C_{20:0}$ ($C_{20}H_{40}O_2$)	311.30	311.30	-9.64	47	311.28	-65.53	567	311.29	-28.59	54
	Heneicosenoic acid $C_{21:1}$ ($C_{21}H_{40}O_2$)	323.30	-	-	-	323.27	-104.55	146	323.29	-44.85	37
Heneicosanoic acid, methyl ester	Heneicosanoic acid $C_{21:0}$ ($C_{21}H_{42}O_2$)	325.31	-	-	-	325.29	-61.48	224	325.31	-12.60	42
Docosanoic acid, methyl ester	Docosanoic acid $C_{22:0}$ ($C_{22}H_{44}O_2$)	339.33	-	-	-	339.32	-43.03	367	339.32	-23.87	52
Tricosanoic acid, methyl ester	Tricosanoic/Tricosylic acid/methyl behenate $C_{23:0}$ ($C_{23}H_{46}O_2$)	353.33	353.34	24.06	29	353.33	-0.85	246	353.34	20.38	41
Tetracosanoic acid, methyl ester	Lignoceric/tetracosanoic acid $C_{24:0}$ ($C_{24}H_{48}O_2$)	367.37	367.37	4.08	30	367.36	-27.22	676	367.36	-38.38	125
	Pentacosenoic acid $C_{25:1}$ ($C_{25}H_{48}O_2$)	379.36	-	-	-	379.36	-12.39	122	379.36	-4.48	33
Methyl 22-methyl-tetracosanoate; Pentacosanoic acid, methyl ester	Pentacosanoic acid $C_{25:0}$ ($C_{25}H_{50}O_2$)	381.37	381.37	8.13	26	381.37	8.65	382	381.37	-6.56	80
	Ximenic acid $C_{26:1}$ ($C_{26}H_{50}O_2$)	393.37	-	-	-	393.38	15.51	111	393.37	-0.76	35
Hexacosanoic acid, methyl ester	Hexacosanoic acid $C_{26:0}$ ($C_{26}H_{52}O_2$)	395.39	-	-	-	395.39	-11.63	298	395.38	-14.92	80
Heptacosanoic acid, methyl ester	Heptacosanoic acid $C_{27:0}$ ($C_{27}H_{54}O_2$)	409.40	-	-	-	409.40	-7.82	99	409.40	0.24	31
	Octacosanoic acid $C_{28:1}$ ($C_{28}H_{54}O_2$)	421.41	-	-	-	421.41	11.63	86	421.40	-27.53	23
Octacosanoic acid, methyl ester	Octacosanoic acid $C_{28:0}$ ($C_{28}H_{56}O_2$)	423.43	-	-	-	423.41	-45.34	71	423.41	-49.36	26
	Noacosanoic acid $C_{29:0}$ ($C_{29}H_{58}O_2$)	437.44	-	-	-	437.41	-60.12	45	437.44	-2.97	12
Triacontanoic acid, methyl ester	Triacontanoic acid $C_{30:0}$ ($C_{30}H_{60}O_2$)	451.46	-	-	-	451.45	-25.69	43	451.45	-22.59	15
Methyl 2-hydroxytetracosanoate, TMS derivative (C28H58O3Si)	Hydroxy tetracosanoic acid ($C_{24}H_{48}O_3$)	383.35	383.36	16.17	18	383.36	16.17	69	383.38	73.82	15
Methyl 2-hydroxypentacosanoate, TMS derivative (C29H60O3Si)	Hydroxy pentacosanoic acid ($C_{25}H_{50}O_3$)	397.37	397.36	-17.62	19	397.37	2.01	60	397.41	90.60	7
Methyl 2-hydroxy-hexacosanoate, trimethylsilyl ether (C30H62O3Si)	Hydroxy hexacosanoic acid ($C_{26}H_{52}O_3$)	411.38	411.37	-30.87	14	411.38	11.91	31	411.40	57.85	14

Table S4. Fatty acid list identified in sample 010109-1 collected in the Upper Gossan deposits of Peña de Hierro using ToF-SIMS throught the main [M - H]$^-$ ion. Letters bolded in red mark the most abundant fatty acids in terms of intensity (cps) (See Fig. 9).

Head groups (TA1 to TA3 average mass)			
Measured (m/z+)	Calculated (m/z+)	Error (ppm)	Fragment formula
30.04	30.03	-213.12	CH4N+
58.07	58.07	22.39	C3H8N+
59.07	59.07	-22.01	C3H9N+
72.08	72.08	-33.30	C4H10N+
86.10	86.10	31.36	C5H12N+
102.10	102.09	-64.65	C5H12NO+
104.11	104.11	19.21	C5H14NO+
125.00	125.00	32.80	C2H6PO4+
166.05	166.06	71.06	C5H13NPO3+
184.09	184.07	-119.52	C5H15NPO4+

Carbonyl fragments (TA1 to TA3 average mass)			
Measured (m/z+)	Calculated (m/z+)	Error (ppm)	Fragment formula
211.22	211.21	-47.35	C14H27O+
239.25	239.24	-41.80	C16H31O+
253.25	253.25	-9.08	C17H33O+
265.26	265.25	-37.70	C18H33O+
267.28	267.27	-37.42	C18H35O+
309.31	309.32	32.33	C21H41O+

Monoacylglyceride fragments in TA2			
Measured (m/z+)	Calculated (m/z+)	Error (ppm)	Fragment formula
311.28	311.26	-64.25	C19H35O3+
313.29	313.27	-63.84	C19H37O3+
339.27	339.29	58.95	C21H39O3+
353.30	353.31	28.30	C22H41O3+
355.31	355.32	23.36	C22H43O3+
367.33	367.32	-22.05	C23H43O3+

Dyacylglyceride fragments in TA2			
Measured (m/z+)	Calculated (m/z+)	Error (ppm)	Fragment formula
467.42	467.41	-21.39	C29H55O4+
479.41	479.41	0.63	C30H55O4+
481.45	481.43	-35.31	C30H57O4+
493.43	493.43	5.27	C31H57O4+
495.45	495.44	-20.18	C31H59O4+
507.45	507.44	-19.71	C32H59O4+
509.47	509.46	-13.94	C32H61O4+
521.47	521.46	-19.18	C33H61O4+
523.48	523.47	-19.10	C33H63O4+
533.47	533.46	-18.75	C34H61O4+
535.49	535.47	-37.35	C34H63O4+
537.47	537.49	37.21	C34H65O4+
547.48	547.47	-18.27	C35H63O4+
549.50	549.49	-13.10	C35H65O4+
551.51	551.50	-18.13	C35H67O4+
563.52	563.50	-35.49	C36H67O4+
565.53	565.52	-17.68	C36H69O4+
575.51	575.50	-17.38	C37H67O4+
577.54	577.52	-26.49	C37H69O4+
579.55	579.54	-11.04	C37H71O4+
593.57	593.55	-33.70	C38H73O4+
605.56	605.55	-16.51	C39H73O4+
607.58	607.57	-16.46	C39H75O4+
619.59	619.57	-32.28	C40H75O4+
621.60	621.58	-32.18	C40H77O4+
633.59	633.58	-15.78	C41H77O4+
635.59	635.60	15.73	C41H79O4+
647.63	647.60	-46.32	C42H79O4+
649.61	649.61	-1.54	C42H81O4+
661.62	661.61	-14.36	C43H81O4+
663.67	663.63	-60.27	C43H83O4+
675.64	675.63	-14.80	C44H83O4+
677.69	677.64	-73.79	C44H85O4+
689.68	689.64	-58.00	C45H85O4+
691.68	691.66	-28.92	C45H87O4+
703.69	703.66	-42.63	C46H87O4+
705.71	705.68	-42.51	C46H89O4+

Major glycerophospholipid anions in TA2			
Measured (m/z-)	Calculated (m/z-)	Error (ppm)	Fragment formula
700.53	700.53	-6.28	$C_{39}H_{75}NO_7P^-$
712.47	712.46	-18.25	$C_{38}H_{67}NO_9P^-$
726.51	726.51	2.06	$C_{40}H_{73}NO_8P^-$
730.56	730.54	-22.31	$C_{40}H_{77}NO_8P$-
749.46	749.46	-3.20	$C_{38}H_{70}O_{12}P^-$

Table S5. Different positive and negative ions of acylglycerides obtained through the ToF-SIMS spectral analysis of the Upper Gossan deposits in Peña de Hierro. The acylglycerid peaks are well defined in the biological filamentous structures of Group 9 found in TA2.

Measured (m/z⁺)	Calculated (m/z)⁺	Error (ppm)	Formula	Compound	Fragment
264.27	264.27	18.92	C18H34N+	sphingosine/sphingenine	$[M + H - 2H_2O]^+$
266.29	266.28	-37.55	C18H36N+	sphinganine	$[M + H - 2H_2O]^+$
282.29	282.28	-47.47	C18H36NO+	sphingosine/sphingenine	$[M + H - H_2O]^+$
284.32	284.30	-87.58	C18H38NO+	sphinganine	$[M + H - H_2O]^+$
298.28	298.27	-25.48	C18H36NO2+	sphingosine/sphingenine	$[M - H]^+$
308.31	308.30	-41.19	C20H38NO+	amino-methyl-nonadece-triol	$[M + H - 2H_2O]^+$
310.33	310.31	-64.45	C20H40NO+	phytosphingosine	$[M + H - 2H_2O]^+$

Calculated (m/z)⁺	TA1		TA2 & TA3 average		Fragment formula
	Measured (m/z⁺)	Error (ppm)	Measured (m/z⁺)	Error (ppm)	
534.56	534.57	-18.71	534.58	-37.41	C36H72NO+
562.56	562.57	-17.78	562.58	-35.55	C37H72NO2+
564.57	564.61	-70.85	564.61	-70.85	C37H74NO2+
566.59	566.62	-52.95	-	-	C37H76NO2+
568.63	568.65	-35.17	568.63	-6.16	C39H80NO+
590.59	590.59	-5.59	590.60	-21.84	C39H76NO2+
596.54	-	-	596.57	-50.29	C40H70NO2+
604.60	604.64	-68.14	604.62	-30.93	C40H78NO2+
612.59	612.57	26.12	612.58	14.20	C38H78NO4+
618.62	618.63	-22.63	618.58	62.72	C41H80NO2+
624.59	624.61	-39.55	624.60	-9.61	C39H78NO4+
632.63	632.63	-5.37	632.61	31.61	C42H82NO2+
638.61	638.60	15.66	638.59	31.32	C40H80NO4+
640.59	640.58	17.80	640.58	9.83	C39H78NO5+
652.62	-	-	652.61	20.38	C41H82NO4+
654.60	654.61	-19.25	654.60	-5.81	C40H80NO5+
666.60	666.61	-21.90	666.61	-17.70	C41H80NO5+
668.62	668.61	18.55	668.63	-21.99	C41H82NO5+
682.63	682.64	-7.91	682.62	9.67	C42H84NO5+
696.65	696.65	-2.58	696.67	-25.12	C43H86NO5+
710.63	710.63	1.55	710.64	-19.56	C43H84NO6+.
714.66	714.64	25.19	714.65	16.23	C43H88NO6+

Table S6. Different positive fragments of sphingolipids (upper table) and ceramides (lower table) found in sample 010109-1 through ToF-SIMS spectral analysis.

Fragment	TA1		TA2		TA3	
	m/z^+	Intensity (%)	m/z^+	Intensity (%)	m/z^+	Intensity (%)
CH2N+	28.03	7.50	28.03	3.51	28.03	2.95
C2H4N+	42.04	14.80	42.04	9.50	42.04	5.97
C3H7+	43.06	57.80	43.06	69.00	43.06	39.98
C2H6N+	44.06	17.30	44.05	7.50	44.05	2.50
C3H6N+	56.06	12.00	56.06	8.50	56.06	7.10
CH3N3+	57.04	7.00	57.03	5.00	57.03	4.00
C5H10N+	84.09	2.46	84.09	1.50	84.09	0.91
C3H8N3+	86.06	0.90	86.06	0.66	86.06	0.60
C5H12N+	86.10	0.87	86.10	0.55	86.10	0.65
C6H10N2+	110.09	0.95	110.08	0.32	110.09	0.46
C5H10N3+	111.10	1.00	111.09	0.72	111.10	0.80
C5H10N3+	112.10	0.48	112.09	0.66	112.10	1.00
C8H10N+	120.09	0.30	120.09	1.15	-	-
C6H8N3+	122.08	0.15	122.08	0.18	122.08	0.25
C7H11N2+	123.10	0.64	123.09	0.43	-	-
C6H10N3+	124.10	0.38	124.09	0.25	124.09	0.60
C7H13N2+	125.12	0.95	125.11	0.65	-	-
C8H11N2+	135.10	0.70	135.09	0.30	135.12	2.08
C7H10N3+	136.10	0.22	136.09	0.16	136.09	1.51
C7H11N3+	137.11	0.27	137.11	0.22	137.10	1.65
C7H12N3+	138.12	0.26	138.10	0.20	138.10	0.31

Fragment	TA1		TA2		TA3	
	m/z^-	Intensity (%)	m/z^-	Intensity (%)	m/z^-	Intensity (%)
C5H5N-	93.04	0.15	93.04	0.15	93.04	0.90
C4H5O4-	117.03	0.07	117.03	0.06	117.03	0.15
C13H9NO2-	211.06	0.09	211.06	0.07	-	-
C13H10NO2-	221.07	0.02	212.06	0.02	-	-
C18H19N2O-	279.16	0.17	279.15	0.09	217.16	0.05
C18H20N2O-	280.16	0.03	280.16	0.02	280.16	0.01

Table S7. List of positive and negative ions produced from the fragmentation of peptidic chains and/or amino acids that have been obtained by the ToF-SIMS spectral analysis of sample 010109-1 collected in the Upper Gossan of Pena de Hierro.

Compound	Measured (m/z)	Molecular formula	Ion fragment	Intensity (cps)	Target area
Steroid fragments	147.11	$C_{11}H_{15}^+$	-	< 1975	TA3, TA1, TA2
	161.14	$C_{12}H_{17}^+$	-	< 1070	TA3, TA2, TA1
	201.16	$C_{15}H_{21}^+$	-	< 235	TA3, TA1, TA2
	215.18	$C_{16}H_{23}^+$	-	< 150	TA3, TA2, TA1
	149.13	$C_{11}H_{17}^+$	-	< 990	TA3, TA2, TA1
	191.18	$C_{14}H_{23}^+$	-	< 140	TA3, TA2, TA1
	203.18	$C_{15}H_{23}^+$	-	< 140	TA3, TA1, TA2
	257.22	$C_{19}H_{29}^+$	-	< 60	TA1, TA2
Cholesterol	369.39	$C_{27}H_{45}^+$	$[M - OH]^+$	< 238	TA2, TA3
	386.33	$C_{27}H_{46}O^+$	M^+	< 55	TA3, TA2, TA1
Cholesterol sulfate	465.30	$C_{27}H_{45}SO_4^-$	$[M + SO_3^- H]^+$	< 1050	TA2, TA1
	466.30	$C_{27}H_{46}SO_4^-$	$[M + SO_3]^+$	< 330	TA2, TA1
	467.30	$C_{27}H_{47}SO_4^-$	$[M + SO_3^+ H]^+$	< 145	TA2, TA1
Stigmasterol ($C_{29}H_{48}O$)	411.39	$C_{29}H_{47}O^+$	$[M - H]^+$	< 31	TA2
	412.38	$C_{29}H_{48}O^+$	M^+	< 25	TA3, TA2
Stigmasterol derivative ($C_{29}H_{46}O$)	409.35	$C_{29}H_{45}O^+$	$[M - H]^+$	< 60	TA2
	410.35	$C_{29}H_{46}O^+$	M^+	< 25	TA2
Ergosterol ($C_{28}H_{44}O$)	395.33	$C_{28}H_{43}O^+$	$[M - H]^+$	< 71	TA2, TA3
	396.34	$C_{28}H_{44}O^+$	M^+	< 25	TA2
Campesterol ($C_{28}H_{48}O$)	400.37	$C_{28}H_{48}O^+$	M^+	< 25	TA1, TA2, TA3

Table S8. List of fragments attributed to different steroids that have been recognized in the Upper Gossan sample 010109-1 through the ToF-SIMS spectral analysis.

Measured (m/z^+)	Calculated (m/z^+)	Error (ppm)	Formula	Intensity (cps)	Target area
76.03	76.03	19.73	C6H4+	< 1500	TA1, TA2/TA3
77.04	77.04	55.82	C6H5+	< 8836	TA1, TA2, TA3
104.03	104.03	45.18	C7H4O+	< 823	TA1, TA3
105.03	105.03	-38.08	C7H5O+	< 1500	TA3/TA1, TA2
149.02	149.02	3.94	C8H5O3+	< 1969	TA3, TA1, TA2
149.06	149.06	6.71	C9H9O2+	< 870	TA3, TA1, TA2
150.03	150.03	33.33	C8H6O3+	< 256	TA3/TA1, TA2
163.04	163.04	-12.27	C9H7O3+	< 367	TA3, TA1, TA2
165.06	165.06	-4.24	C9H9O3+	< 1250	TA1, TA3, TA2
166.04	166.05	57.21	C5H10O6+	< 1143	TA1, TA2, TA3
168.04	167.03	-61.67	C8H8O4+	< 532	TA1, TA3, TA2
169.09	169.09	-11.24	C9H13O3+	< 434	TA3, TA1, TA2
179.07	179.07	-25.13	C10H11O3+	< 658	TA3, TA1, TA2

Measured (m/z^-)	Calculated (m/z^-)	Error (ppm)	Formula	Intensity (cps)	Target area
73.03	73.03	41.08	C3H5O2-	< 1972	TA2, TA3, TA1
77.04	77.04	-24.66	C6H5-	< 2697	TA3, TA1, TA2
105.04	105.03	-57.13	C7H5O-	< 898	TA3, TA1, TA2
120.02	120.02	14.16	C7H4O2-	< 436	TA1, TA3, TA2
121.03	121.03	-2.48	C7H5O2-	< 3347	TA3, TA1, TA2
163.05	163.06	37.41	C6H11O5-	< 1475	TA2, TA3, TA1
165.03	165.04	85.43	C8H5O4-	< 860	TA3, TA2, TA1

Table S9. List of positive and negative ions from the fragmentation of polyphenolic and phytochemical compounds obtained by the ToF-SIMS spectral analysis of sample 010109-1 collected in the Upper Gossan of Peña de Hierro.
